# E3 SUMO ligase SIZ1 splicing variants localize and function according to external conditions

**DOI:** 10.1093/plphys/kiae108

**Published:** 2024-03-18

**Authors:** Jun Soo Kwak, Jong Tae Song, Hak Soo Seo

**Affiliations:** Department of Agriculture, Forestry and Bioresources, Research Institute of Agriculture and Life Sciences, Seoul National University, Seoul 08826, Korea; Department of Applied Biosciences, Kyungpook National University, Daegu 41566, Korea; Department of Agriculture, Forestry and Bioresources, Research Institute of Agriculture and Life Sciences, Seoul National University, Seoul 08826, Korea; Bio-MAX Institute, Seoul National University, Seoul 08826, Korea

## Abstract

SIZ1 (SAP and MIZ1) is a member of the Siz/PIAS-type RING family of E3 SUMO (small ubiquitin-related modifier) ligases that play key roles in growth, development, and stress responses in plant and animal systems. Nevertheless, splicing variants of *SIZ1* have not yet been characterized. Here, we identified four splicing variants of Arabidopsis (*Arabidopsis thaliana*) *SIZ1*, which encode three different protein isoforms. The *SIZ1* gene encodes an 873-amino acid (aa) protein. Among the four *SIZ1* splicing variants (*SSV*s), *SSV1* and *SSV4* encode identical 885 aa proteins; *SSV2* encodes an 832 aa protein; and *SSV3* encodes an 884 aa protein. SSV2 mainly localized to the plasma membrane, whereas SIZ1, SSV1/SSV4, and SSV3 localized to the nucleus. Interestingly, SIZ1 and all SSVs exhibited similar E3 SUMO ligase activities and preferred SUMO1 and SUMO2 for their E3 ligase activity. Transcript levels of *SSV2* were substantially increased by heat treatment, while those of *SSV1*, *SSV3*, and *SSV4* transcripts were unaffected by various abiotic stresses. SSV2 directly interacted with and sumoylated cyclic nucleotide-gated ion channel 6 (CNGC6), a positive thermotolerance regulator, enhancing the stability of CNGC6. Notably, transgenic *siz1-2* mutants expressing *SSV2* exhibited greater heat stress tolerance than wild-type plants, whereas those expressing *SIZ1* were sensitive to heat stress. Furthermore, transgenic *cngc6* plants overaccumulating a mutated mCNGC6 protein (K347R, a mutation at the sumoylation site) were sensitive to heat stress, similar to the *cngc6* mutants, while transgenic *cngc6* plants overaccumulating CNGC6 exhibited restored heat tolerance. Together, we propose that alternative splicing is an important mechanism that regulates the function of SSVs during development or under adverse conditions, including heat stress.

## Teaser

Splicing variants of an E3 SUMO ligase gene specifically accumulate under certain growth conditions, and proteins encoded by these splicing variants function in different subcellular compartments.

## Introduction

Sumoylation is a reversible ubiquitin (Ub)-like protein conjugation process that catalyzes the attachment of SUMO to a target substrate in the nucleus or cytoplasm (Kerscher et al. 2006). Sumoylation regulates protein activity, stability, and localization as well as protein–protein interactions (Celen and Sahin 2020). SUMOs are small polypeptides (10 to 15 kDa) that exhibit structural resemblance to Ub with limited sequence similarity (Cappadocia and Lima 2018). In plants, sumoylation is rapidly triggered by multiple abiotic stresses such as heat, drought, and salt stress (Kurepa et al. 2003; Augustine and Vierstra 2018; Benlloch and Lois 2018). Sumoylation involves three steps that occur in the following sequence: SUMO E1 activation, E2 conjugation, and E3 ligation (Kerscher et al. 2006). SIZ1 is a Siz/PIAS (SP)-REAL INTERESTING NEW GENE (RING) finger protein containing the SAP and MIZ domains. In plants, SIZ1 acts as an E3 SUMO ligase to mediate stress response and plant development. Studies show that SIZ1 is involved in gibberellin (GA) and cytokinin signaling (Kim et al. 2015a; Coleman et al. 2020) and the response to heat stress (Mishra et al. 2017; Zhang et al. 2018) and drought stress (Mishra et al. 2017; Zhang et al. 2017). Overexpression of SIZ1 has been shown to increase stress tolerance in rice (*Oryza sativa*) (Li et al. 2013; Mishra et al. 2017), tobacco (*Nicotiana benthamiana*) (Zhang et al. 2017), and tomato (*Solanum lycopersicum*) (Zhang et al. 2018). A number of SIZ1 target proteins have been isolated and characterized to date. For example, PHOSPHATE STARVATION RESPONSE 1 (PHR1) is involved in the phosphate starvation response (Miura et al. 2005); INDUCER OF C-REPEAT BINDING FACTOR (CBF) EXPRESSION 1 (ICE1) in freezing tolerance (Miura et al. 2007); NITRATE REDUCTASE 1 (NIA1) and NIA2 in nitrogen assimilation (Park et al. 2011); ABSCISIC ACID (ABA)-INSENSITIVE 5 (ABI5) and R2R3-type transcription factor MYB30 in ABA signaling (Miura et al. 2009; Zheng et al. 2012); FLOWERING LOCUS C (FLC) in flowering repression (Son et al. 2014); FLOWERING LOCUS D (FLD) in histone demethylation (He et al. 2003; Jin et al. 2008); CHROMOMETHYLASE 3 (CMT3) in DNA methylation (Kim et al. 2015b); SLEEPY 1 (SLY1) in GA signaling (Kim et al. 2015a); and DEMETER (DME) and REPRESSOR OF SILENCING 1 (ROS1) in DNA demethylation (Kong et al. 2020). Recently, proteomics analysis showed that SIZ1 directly affects the sumoylation of over 100 proteins including chromatin remodeling enzymes, transcription factors, and heat shock proteins (Rytz et al. 2018). The stability of SIZ1 is controlled by its polyubiquitination through the E3 Ub ligase activity of CONSTITUTIVE PHOTOMORPHOGENIC 1 (COP1) and the 26S proteasome complex (Kim et al. 2016).

Alternative splicing is a process in which different combinations of exons, parts of exons, and/or parts of introns are included in the mature mRNA, resulting in more than one unique mRNA species from a single gene (Kelemen et al. 2013; Baralle and Giudice 2017; Furlanis and Scheiffele 2018; Bowler and Oltean 2019). Alternative splicing can affect mRNA stability, localization, or translation (Baralle and Giudice 2017). In addition, proteins translated from alternatively spliced mRNA isoforms are distinct from the authentic protein in structure, function, and consequently physiological properties such as intracellular localization and enzymatic activity (Kelemen et al. 2013), which indicates that alternative splicing generates different protein isoforms with diverse functions and/or localization patterns. Alternative splicing can create not only a functionally active variant but also an inactive one, which can repress the function of the active variant (so-called dominant negative effect) (Kelemen et al. 2013). Alternative splicing can also result in the synthesis of noncoding mRNAs, which can modulate the function of protein-coding mRNAs by competing with them to bind to their regulators (Baralle and Giudice 2017).

Increasing evidence shows that SIZ1 regulates plant growth and stress response by modulating the function and stability of target proteins through its E3 ligase activity. Database predicted by computer programs suggests the possibility that SIZ1 participates in various signal transduction pathways through its spliced isoforms. Here, we report, that a splicing variant of SIZ/PIAS-type E3 SUMO ligases, SSV2, is responsible for stress-specific response. Our results revealed the accumulation of *SSV2* transcripts in response to heat stress. SSV2 protein sumoylated the cyclic nucleotide-gated ion channel 6 (CNGC6) protein through their E3 ligase activity, and CNGC6 was stabilized in *SSV2*-overexpressing transgenic plants. In addition, *SSV2*-expressing transgenic *siz1-2* mutants were more tolerant to heat stress than WT plants, whereas *SIZ1*-expressing transgenic *siz1-2* mutants did not exhibit heat tolerance. Moreover, transgenic *cngc6* plants expressing a mutant protein at sumoylation site, mCNGC6, were sensitive to heat stress, similar to *cngc6* mutants, indicating that, among SIZ1 and its splicing variants, SSV2 is the primary regulator of heat tolerance in Arabidopsis (*Arabidopsis thaliana*).

## Results

### Predicted alternatively spliced variants of Arabidopsis *SIZ1* gene

**Figure 1.**
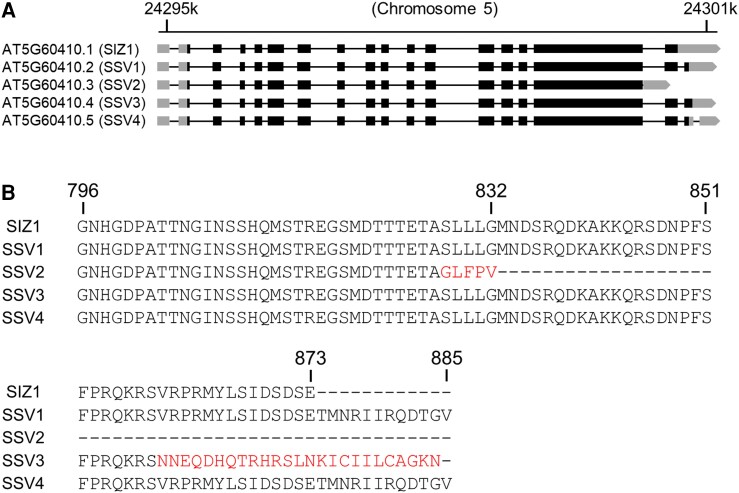
Comparison of the gene structures of *SIZ1* and its splicing variants (*SSV1* to *SSV4*) and their C-terminal amino acid sequences. **A)** Schematic representation of the genomic regions of *SIZ1* (AT5G60410.1) and its splicing variants SSV1 (AT5G60410.2), SSV2 (AT5G60410.3), SSV3 (AT5G60410.4), and SSV4 (AT5G60410.5), as reported in NCBI and TAIR databases. Black and gray boxes represent exons and untranslated regions (UTRs), respectively, and black lines represent introns. **B)** Comparison of the deduced amino acid sequences of the C-terminal regions of SIZ1 and SSVs.

Four alternatively spliced variants of Arabidopsis *SIZ1* were identified in the NCBI (https://www.ncbi.nlm.nih.gov/) and TAIR (http://www.arabidopsis.org/) databases: *SSV1*, *SSV2*, *SSV3*, and *SSV4* (Fig. 1A). The gene structure of *SIZ1* shows that *SSV1*, *SSV2*, *SSV3*, and *SSV4* are produced by alternative splicing at the last exon and intron. All splicing variants encode E3 SUMO ligases but with different molecular weights and C-terminal regions. SIZ1 is composed of 873 aa, while SSV1 and SSV4 are identical with 885 aa (Fig. 1B). SSV2 is composed of 832 aa with five specific amino acids at C-terminal end, whereas SSV3 is composed of 884 aa with 25 specific amino acids at the C-terminal end (Fig. 1B).

### Subcellular localization of SIZ1 and SSVs

**Figure 2.**
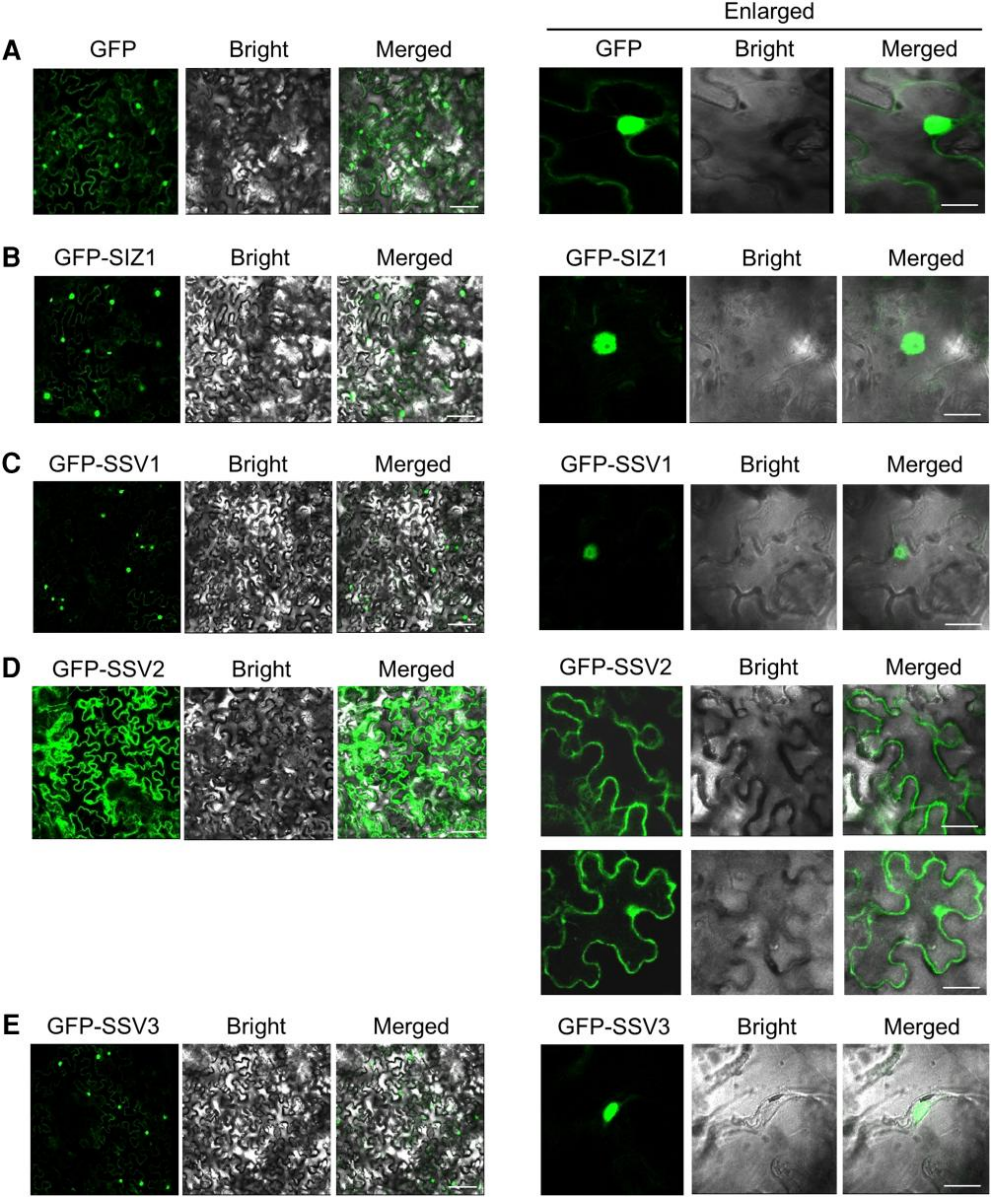
Analysis of the subcellular localization of SIZ1, SSV1, SSV2, and SSV3. (**A** to **E**) Subcelluar location analysis of GFP, SIZ1, SSV1, SSV2, and SSV3. The *35S-GFP* (control), *35S-GFP-SIZ1*, *35S-GFP-SSV1*, *35S-GFP-SSV2*, and *35S-GFP-SSV3* constructs were introduced into *N. benthamiana* leaves by agroinfiltration. After incubation at 25 °C for 2 d, GFP signal was detected by CLSM. DIC, differential interference contrast. Scale bar: 20 *μ*m (left), 10 *µ*m (right).

To determine the role of the C-terminal end of SIZ1 in its function, we first examined the subcellular localization patterns of SIZ1 and SSVs. The *35S-GFP* (control), *35S-GFP-SIZ1*, *35S-GFP-SSV1*, *35S-GFP-SSV2*, and *35S-GFP-SSV3* constructs were agroinfiltrated into *N. benthamiana* leaves, and GFP signal was detected by CLSM. Because the amino acid sequence of SSV4 was identical to that of SSV1, the subcellular localization of SSV4 was not examined. Fluorescence signals of GFP-SIZ1, GFP-SSV1, and GFP-SSV3 were detected in the nucleus (Fig. 2, A to C and E), whereas that of GFP-SSV2 was predominantly detected in the plasma membranes and to some extent in the nucleus (Fig. 2D).

**Figure 3.**
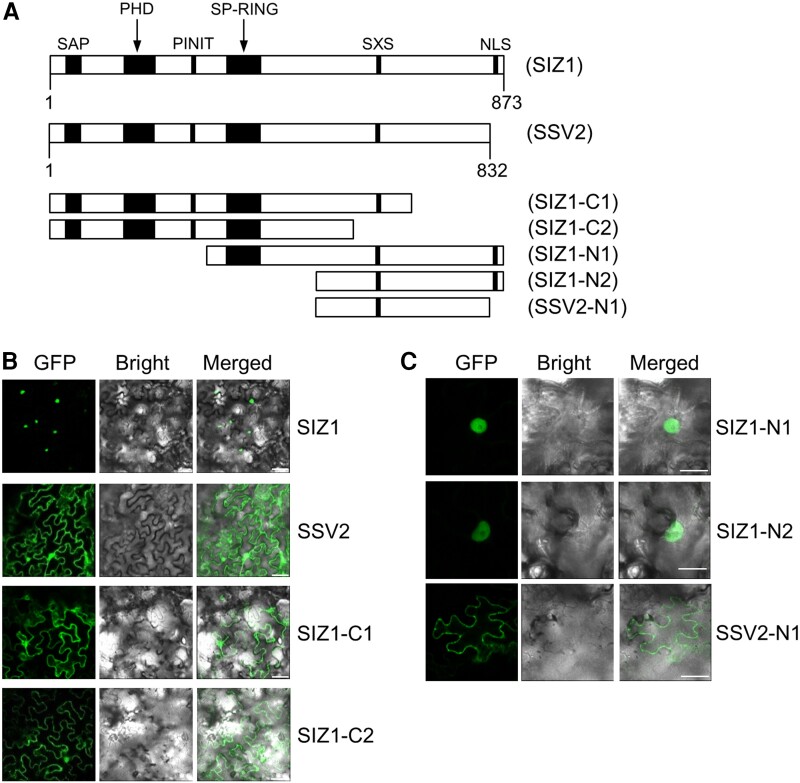
Determination of the conserved domains of SIZ1 and SSV2 responsible for their subcellular localization. **A)** Schematic representation of full-length SIZ1 and SSV2 proteins, N-terminal deletion mutant proteins of SIZ1 and SSV2, and C-terminal deletion mutant proteins of SIZ1. **B, C)** Subcellular location analysis of SIZ1, SSV2, and the C-terminal deletion mutant proteins of SIZ1 **(B)**, and that of the N-terminal deletion mutant proteins of SIZ1 and SSV2 **(C)**. The *35S-GFP-SIZ1*, *35S-GFP-SSV2*, *35S-GFP-SIZ1-C1*, *35S-GFP-SIZ1-C2*, *35S-GFP-SIZ1-N1*, *35S-GFP-SIZ1-N2*, and *35S-GFP-SSV2-N1* constructs were separately introduced into *N. benthamiana* leaves by agroinfiltration. After incubation at 25 °C for 2 d, GFP signal was detected by CLSM. Scale bars: 20 *µ*m **(B)**, 10 *µ*m **(C)**.

The SIZ1 protein contains various functional motifs including a putative nuclear localization signal (NLS) at the C-terminal end. To examine their effect on the subcellular localization of SIZ1, we generated two C-terminal deletion mutant proteins of SIZ1 (SIZ1-C1 and SIZ1-C2) (Fig. 3A). The resulting constructs, *35S-GFP-SIZ1*, *35S-GFP-SSV2*, *35S-GFP-SIZ1-C1*, and *35S-GFP-SIZ1-C2*, were agroinfiltrated into *N. benthamiana* leaves, and GFP signals were detected by CLSM. Deletion mutant proteins were mainly detected in the plasma membranes, like SSV2, whereas GFP-SIZ1 was detected in the nucleus (Fig. 3B). We also generated two N-terminal deletion mutant proteins of SIZ1 (SIZ1-N1 and SIZ1-N2), and one N-terminal deletion mutant protein of SSV2 (SSV2-N1) (Fig. 3A). Each of the *35S-GFP-SIZ1-N1*, *35S-GFP-SIZ1-N2*, and *35S-GFP-SSV2-N1* constructs was agroinfiltrated into *N. benthamiana* leaves. Fluorescence signals of GFP-SIZ1-N1 and GFP-SIZ1-N2 were detected in the nucleus, while that of GFP-SSV2-N1 was detected in the plasma membrane (Fig. 3C).

### NLS determines the subcellular localization of SIZ1

The above results strongly indicate that the NLS motif located at the C-terminal end of SIZ1 plays a crucial role in the nuclear localization of SIZ1. To verify this result, we produced two specific C-terminal deletion mutant proteins of SIZ1 (SIZ1-CC1 and SIZ1-CC2), based on the NLS amino acid sequence (Supplementary Fig. S1A). We also generated an in-frame fusion of calmodulin-like protein 35 (CML35), a cytosolic protein (Dell’Aglio et al. 2016), with the NLS peptide (from 835th to 873rd) of SIZ1 (CML35-NLS) (Supplementary Fig. S1B). First, *35S-GFP-SIZ1-CC1* and *35S-GFP-SIZ1-CC2* constructs were separately introduced into *N. benthamiana* leaves, and GFP signals were detected by CLSM. While GFP-SIZ1-CC1 was detected in the nucleus, GFP-SIZ1-CC2 was detected in the cytoplasmic membrane (Supplementary Fig. S1C). Next, the *35S-GFP-CML35-NLS* construct was introduced into *N. benthamiana* leaves, and GFP signal was detected by CLSM. The results showed that GFP-CML35-NLS localized to the nucleus, like SIZ1 (Supplementary Fig. S1D).

SSV2 contains the GLFPV peptide at its C-terminal end. To determine the effect of the SSV2-specific peptide on its subcellular localization, we fused the DNA sequence encoding the GLFPV peptide to the 3′-end of *SIZ1* (*SIZ1-A1*) (Supplementary Fig. S1A). The *35S-GFP-SIZ1* and *35S-GFP-SIZ1-A1* constructs were individually infiltrated into *N. benthamiana* leaves, and GFP signals were detected by CLSM. Both GFP-SIZ1 and GFP-SIZ1-A1 localized to the nucleus (Supplementary Fig. S1E). This suggests that SSV2-specific GLFPV peptide has no effect on its subcellular localization.

### SSV2 associates with the cell membrane but not with the cell wall

Next, we examined the subcellular localization pattern of GFP-SSV2 in further detail. *N. benthamiana* leaves were coinfiltrated either with *35S-GFP-SSV2* and *35S-RFP-PIP2* or with *35S-GFP-SSV2* and *35S-RFP-SYP122*, and were then floated on 1 M NaCl at 2 d post-infiltration. Plasma membrane intrinsic protein 2 (PIP2), an aquaporin, serves as a plasma membrane marker protein (Hachez et al. 2013), and SYNTAXIN OF PLANTS 122 (SYP122) is stably located at the plasma membrane (Nielsen and Thordal-Christensen 2012). Fluorescence analysis of the transformed cells showed that the GFP signal, like the RFP signal, remained associated with the plasma membrane in leaves coinfiltrated with *35S-GFP-SSV2* and *35S-RFP-PIP2* (Supplementary Fig. S2A) as well as in leaves coinfiltrated with *35S-GFP-SSV2* and *35S-RFP-SYP122* (Supplementary Fig. S2B), indicating that the SSV2 protein is associated with the cell membrane, not with the cell wall.

We further sought to confirm the localization of SSV2 in the cell membrane. For this purpose, we isolated nuclear, cytosolic, and plasma membrane fractions from transgenic *siz1-2* plants containing the *35S-HA_3_-SSV2* construct and extracted total proteins from these fractions. HA_3_-SSV2 was assessed through immunoblotting using an anti-HA antibody. The results demonstrated that HA_3_-SSV2 was primarily detected in the plasma membrane fraction and, to a lesser extent, in the nuclear fraction, but it was not present in the cytosolic fraction, consistent with the findings depicted in Fig. 2D (Supplementary Fig. S2C). Histone 3, a nuclear marker protein, was exclusively detected in the nuclear fraction, whereas tubulin, a cytosol marker protein, was solely detected in the cytosolic fraction. Additionally, H^+^-ATPase, a plasma membrane marker protein, was exclusively detected in the plasma membrane fraction. Therefore, these data (Supplementary Fig. S2A-, S to C) indicate that SSV2 mainly localizes in the cell membrane but not in the cytosol and cell wall.

### Self-sumoylation ability of SIZ1 and SSVs

Arabidopsis SIZ1 is an E3 SUMO ligase that can sumoylate itself as well as its target proteins. To explore whether SIZ1 and SSVs exhibit any differences in their sumoylation abilities, we first examined the interaction between Arabidopsis SUMO proteins and SSVs by yeast two-hybrid (Y2H) assays. Interactions of SIZ1 and SSV1 to SSV3 with four Arabidopsis SUMO proteins (SUMO1 to SUMO3 and SUMO5) were tested by coexpressing different combinations of constructs in yeast (*Saccharomyces cerevisiae*). The results showed that SIZ1 and SSV2 interacted with SUMO1, SUMO2, SUMO3, and SUMO5 with similar strengths; however, the interactions of SSV1 or SSV3 with SUMO1, SUMO2, SUMO3, and SUMO5 proteins were relatively weak compared with those of SIZ1 (Supplementary Fig. S3, A and B).

Next, we examined the interaction of SIZ1 and SSV1 to SSV3 with SUMO-conjugating E2 enzyme SCE1 by Y2H assays. The results showed that SIZ1 and all three SSVs interacted with SCE1 with similar strengths (Supplementary Fig. S3, C to E). We also examined the effect of SSV3-specific amino acid residues on its interaction with SUMO1 and SCE1 by generating a deletion mutant protein, SSV3-D1, lacking the SSV3-specific 25 aa at its C-terminal end. In Y2H assays, SSV3 and SSV3-D1 showed no difference in their interactions with SCE1 and SUMO1 (Supplementary Fig. S3, C to E), indicating that SSV3-specific 25 aa do not affect the interaction of SSV3 with SUMO proteins and SCE1.

**Figure 4.**
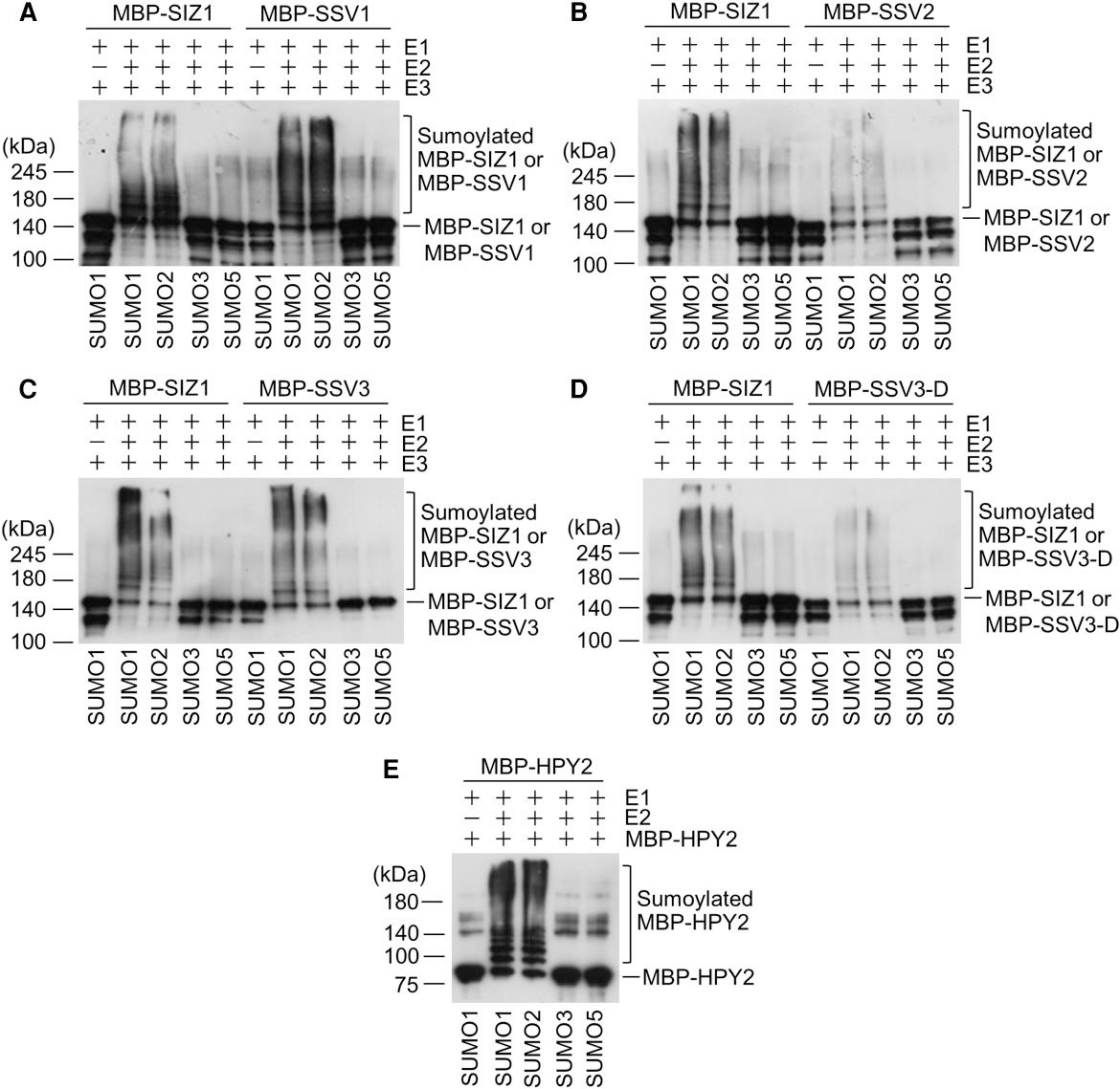
Analysis of the self-sumoylation activity of SIZ1 and SSVs. Constructs expressing His_6_-AtSAE1b, His_6_-AtSAE2, His_6_-AtSCE1, His_6_-tagged SUMO proteins and MBP-tagged SIZ1 variants were introduced into *E. coli*, and recombinant proteins were purified with Ni^2+^-NTA and amylose affinity columns. **A to D)** Self-sumoylation assay of MBP-SIZ1, MBP-SSV1, MBP-SSV2, SSV3, and SSV3-D in the presence of E1 (His_6_-AtSAE1b and His_6_-AtSAE2), E2 (His_6_-AtSCE1), and His_6_-AtSUMO1 (or AtSUMO1 homologs). After the reaction, sumoylated SIZ1, SSV1, SSV2, SSV3, and SSV3-D proteins were detected by immunoblot analysis with anti-MBP antibody. **E)** Self-sumoylation assay of MBP-HPY2. The *MBP-HPY2* construct was introduced into *E. coli*, overexpressed and the recombinant protein was purified with amylose affinity columns, and then used for the sumoylation assay. Self-sumoylated HPY2 was detected by immunoblot analysis with anti-MBP antibody.

Finally, we examined the self-sumoylation activities of SIZ1 and SSVs. In this experiment, we used E3 SUMO ligases (SIZ1, SSV1, SSV2, SSV3, and SSV3-D1) and Arabidopsis SUMO proteins (SUMO1-3 and SUMO5). In vitro sumoylation reactions were performed using His_6_-AtSAE1b (E1), His_6_-AtSAE2 (E1), His_6_-AtSCE1 (E2), His_6_-SUMO1, and MBP-SIZ1 (E3). For other reactions, SUMO1 or maltose-binding protein (MBP)-SIZ1 were replaced by SUMO2, SUMO3, SUMO5 or SSVs. SIZ1, SSV1, SSV2, SSV3, and SSV3-D exhibited self-sumoylation activity in reactions containing SUMO1 or SUMO2, not in those containing SUMO3 or SUMO5 (Fig. 4, A to D). We also analyzed the self-sumoylation activity of MBP-tagged HPY2, which is also an E3 SUMO ligase but much smaller than AtSIZ1, with only 249 aa and only a single domain (SP-RING zinc finger domain) (Ishida et al. 2012; Kwak et al. 2016). Under the same reaction conditions as those used for SIZ1 and SSVs, HPY2 exhibited self-sumoylation activity in the presence of SUMO1 or SUMO2 but not in the presence of SUMO3 or SUMO5 (Fig. 4E).

### Expression of *SIZ1* and *SSVs*

The different localization patterns of SIZ1 and SSV2 imply that the amounts of these proteins are regulated by growth conditions or environmental factors. Therefore, we investigated the relative transcript levels of *SIZ1* and *SSVs* at different developmental stages and under different abiotic stresses and growth conditions. To analyze *SIZ1* and *SSV* transcript levels, we first designed two primer pairs for RT-PCR: 2500F/3013R and 2700F/3280R (Supplementary Fig. S4). The 2500F/3013R primer pair was expected to amplify a 265 bp fragment from *SIZ1*, *SSV1*, *SSV3*, and *SSV4*, and a 513 bp fragment from *SSV2*, while the 2700F/3280R primer pair was predicted to amplify 581, 497, 455, and 412 bp PCR products from *SIZ1*, *SSV1*, *SSV3*, and *SSV4*, respectively, and no PCR product from *SSV2* (Supplementary Fig. S5A).

Total RNA was isolated from samples treated with high and low temperatures, drought stress, high salt concentration, various hormones (α-naphthaleneacetic acid (NAA), GA, ABA, 1-N-naphthylphthalamic acid (NPA), and methyl jasmonate (MeJA)), and two different nitrogen sources including KNO_3_ and (NH_4_)_2_SO_4_. The 2500F/3013R primer pair amplified the 265 and 513 bp PCR products. However, the 513 bp PCR product was amplified only under the high temperature condition (Supplementary Fig. S5B, upper panel), suggesting that heat stress increases the accumulation of *SSV2* transcripts. On the other hand, the 2700F/3280R primer pair amplified the 581 bp PCR product under all conditions but not the 497, 455, and 412 bp fragments (Supplementary Fig. S5B, lower panel). Instead, a novel 828 bp PCR product was amplified specifically under high temperature (Supplementary Fig. S5B, lower panel). This shows that *SSV1*, *SSV3*, and *SSV4* transcript levels are very low under all conditions, and *SSV2* transcripts and 828 bp PCR product are highly accumulated under heat stress.

**Figure 5.**
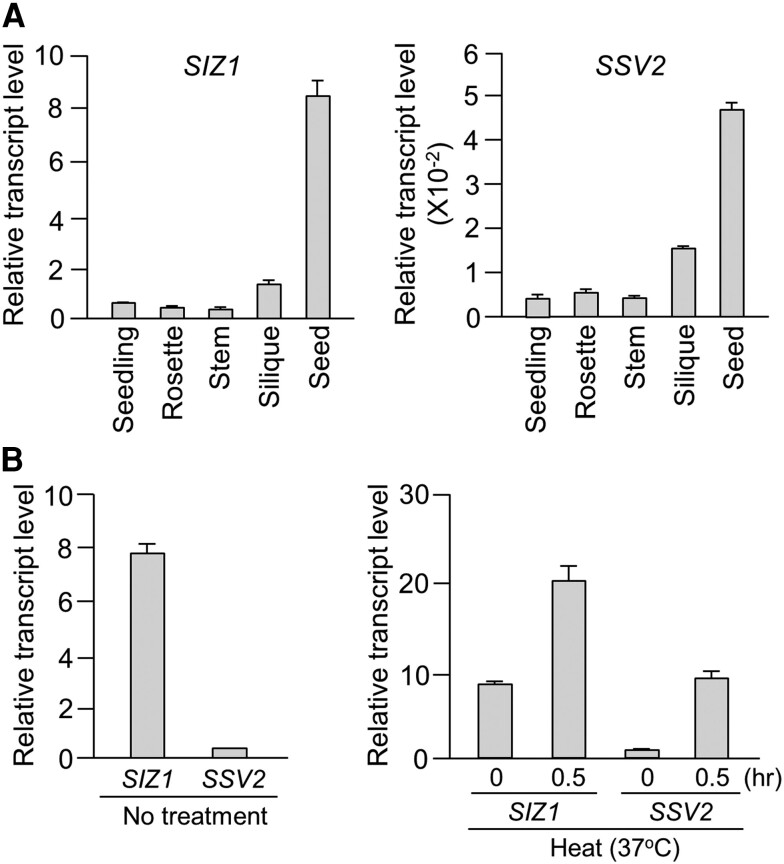
Analysis of *SIZ1* and *SSV2* transcript levels. **A)** RT-qPCR analysis of *SIZ1* and *SSV2* transcripts in whole seedling, rosette leaf, stem, silique, and seed. Error bars indicate standard deviations (Sd, *n* = 3). **B)** Analysis of the transcript levels of *SIZ1* and *SSV2* in leaf samples treated with heat stress for the indicated time periods by RT-qPCR. Error bars indicate standard deviations (Sd, *n* = 3).

Because the accumulation pattern of *SSV2* transcripts differed from those of *SIZ1*, *SSV1*, *SSV3*, and *SSV4*, we further examined the accumulation patterns of *SIZ1* and *SSV2* transcripts in different tissue types including whole seedling, rosette leaf, stem, immature silique, and mature seed. Transcript levels of both *SIZ1* and *SSV2* were the highest in the seed; relatively high in silique; and low in seedling, rosette leaf, and stem (Fig. 5A). Notably, the transcript level of *SIZ1* was approximately 15- to 20-fold higher than that of *SSV2* in all tissues (Fig. 5A). Next, transcript levels of *SIZ1* and *SSV2* were also evaluated under heat stress. Transcript levels of both *SIZ1* and *SSV2* were increased by heat treatment; however, while the transcript level of *SSV2* was increased by approximately 20-fold, that of *SIZ1* was increased by only 2-fold (Fig. 5B, right). Under normal (no stress) condition, *SIZ1* transcript level was approximately 20-fold higher than *SSV2* transcript level (Fig. 5B, left); however, after the heat stress treatment, *SIZ1* transcript level was only approximately 2-fold higher than *SSV2* transcript level (Fig. 5B, right). These results suggest that, under heat stress conditions, SSV2 is the main SIZ1 variant that functions as an E3 SUMO ligase in the plasma membrane, whereas SIZ1 mainly functions in the nucleus.

### Production of *SSV5* under heat stress

**Figure 6.**
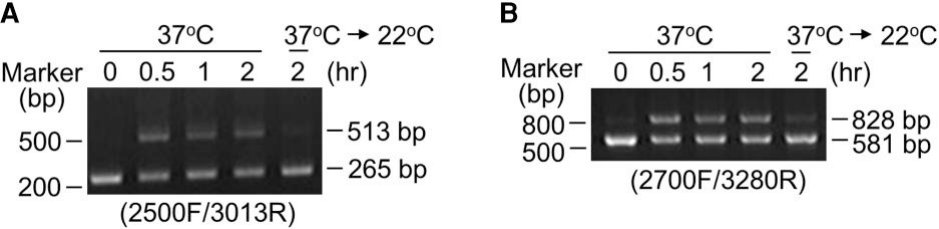
Induction of *SSV2* and *SSV5* expression by heat stress. Total RNAs were isolated from Arabidopsis plants treated with heat stress for the indicated time periods. **A, B)** Analysis of *SSV2* and *SSV5* transcript levels by RT-PCR using the 2500F/3013R primer pair **(A)** and 2700F/3280R primer pair **(B)**. PCR products were analyzed by agarose gel electrophoresis.

Under heat stress, RT-PCR using the 2500F/3013R and 2700F/3280R primer pairs amplified *SSV2* transcripts (513 bp) and a novel splicing variant of *SIZ1* (828 bp), respectively (Supplementary Fig. S5B). To verify this result, we repeated the RT-PCR analyses using samples exposed to heat stress for different durations (0, 0.5, 1, and 2 h). The results of RT-PCR analysis showed that both 513 and 828 bp PCR products were highly accumulated at 0.5 h and were maintained until the 2 h time point; however, the amounts of these PCR products decreased dramatically after the transfer of plants to normal conditions (Fig. 6, A and B). These results indicate that transcripts of *SSV2* and the novel splicing variant are specifically accumulated under heat stress. We named the new splicing variant as *SSV5*.

*SSV5* has not been yet analyzed or documented in NCBI and TAIR database. Therefore, we cloned and sequenced the *SSV5* cDNA. The nucleotide sequence of *SSV5* was identical to that of *SSV2* but contained an additional 613 bp at the 3′ end (Supplementary Fig. S6), indicating that *SSV5* is a novel splicing variant of *SIZ1*. Next, we analyzed the deduced amino acid sequence of SSV5. The deduced amino acid sequence of SSV5 was identical to that of SSV2 (Supplementary Fig. S7), as expected. Like SSV2, SSV5 was composed of 832 aa. Overall, the findings that both *SSV2* and *SSV5* transcripts are highly upregulated under heat stress, and both encode identical proteins strongly suggest that SSV2 protein plays important roles in the heat stress response in Arabidopsis.

### SSV2 physically interacts with CNGC6

**Figure 7.**
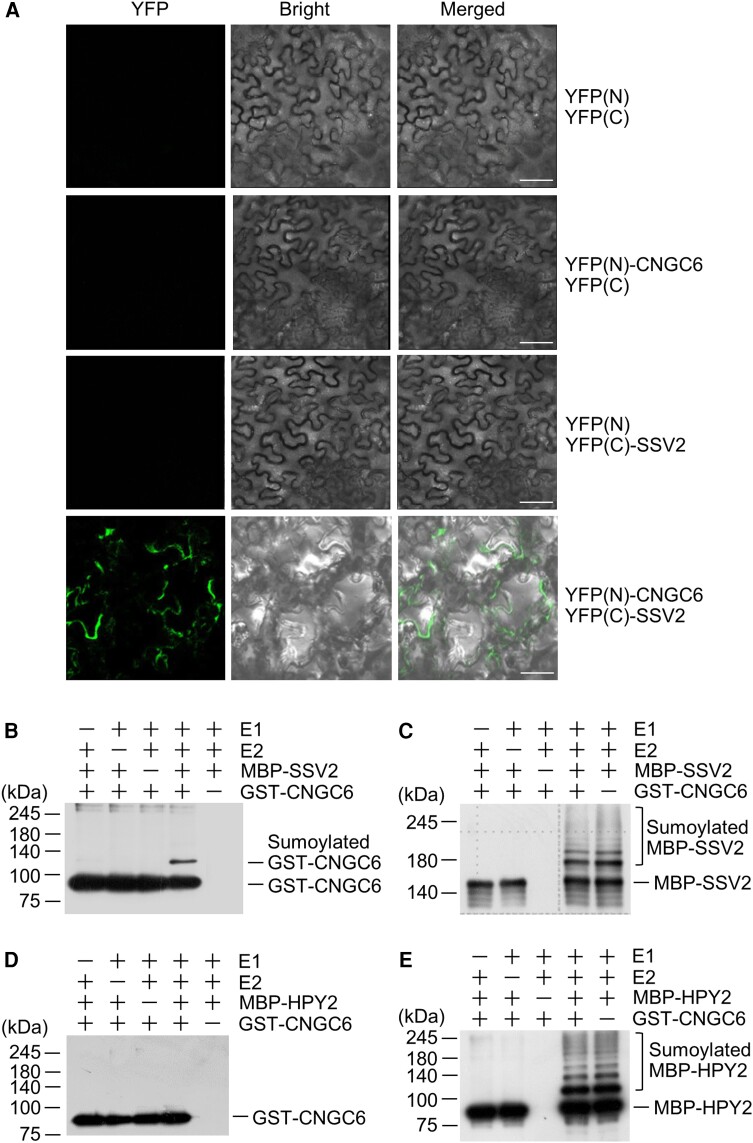
SSV2 physically interacts with sumoylates CNGC6. **A)** Analysis of the interaction between SSV2 and CNGC6 by BiFC assay. *CNGC6* and *CCV2* cDNAs were fused with the N-terminal (N) and C-terminal (C) ends of YFP, respectively. Four constructs in different pairwise combinations (*35S-YFP(N)* and *35S-YFP(C)*, *35S-YFP(N)-CNGC6* and *35S-YFP(C)*, *35S-YFP(N)* and *35S-YFP(C)-SSV2*, and *35S-YFP(N)-CNGC6* and *35S-YFP(C)-SSV2*) were introduced into *N. benthamiana* leaves by agroinfiltration, and fluorescence signals were detected by CLSM. Scale bar, 20 *µ*m. **B, C)** Analysis of the E3 ligase activity of SSV2 against CNGC6 in the presence or absence of His_6_-AtSAE1 + 2, His_6_-AtUBC9, MBP-SSV2, His_6_-AtSUMO1-GG, and GST-CNGC6. After the reaction, sumoylated CNGC6 **(B)** and self-sumoylated SSV2 **(C)** proteins were detected by immunoblot analysis with anti-GST and anti-MBP antibodies, respectively. **D, E)** Analysis of the E3 ligase activity of HPY2 against CNGC6 in the presence or absence of His_6_-AtSAE1 + 2, His_6_-AtUBC9, MBP-HPY2, His_6_-AtSUMO1-GG and GST-CNGC6. After reaction, sumoylated CNGC6 **(D)** and self-sumoylated HPY2 **(E)** were examined by immunoblot analysis with anti-GST and anti-MBP antibodies, respectively.

Accumulation of *SSV2* transcripts under heat stress and localization of the encoded protein to cytoplasmic membranes strongly suggest that SSV2 mediates the sumoylation of cytoplasmic membrane proteins or membrane-linked proteins under heat stress, thus regulating their stability or function. To test this possibility, we chose one of the cytoplasmic membrane proteins, CNGC6, which has been reported to activate the expression of *HSP* genes to improve thermotolerance in Arabidopsis (Gao et al. 2012). To determine whether SSV2 affects CNGC6 protein stability, we first examined potential interaction between SSV2 and CNGC6 by performing the Bimolecular fluorescence complementation (BiFC) assay. *N. benthamiana* leaves were coinfiltrated with four different construct combinations (*35S-YFP(N)* and *35S-YFP(C)*; *35S-YFP(N)-CNGC6* and *35S-YFP(C)*; *35S-YFP(N)* and *35S-YFP(C)-SSV2*; and *35S-YFP(N)-CNGC6* and *35S-YFP(C)-SSV2*), and then subjected to CLSM analysis. Only the leaves coinfiltrated with *35S-YFP(N)-CNGC6* and *35S-YFP(C)-SSV2* showed the YFP signal in the cytoplasmic membranes (Fig. 7A), indicating direct interaction between CNGC6 and SSV2.

### CNGC6 is sumoylated by E3 ligase activity of SSV2

The direct interaction between CNGC6 and SSV2 suggests that SSV2 acts as an E3 SUMO ligase for CNGC6. To determine whether this notion is correct, we produced the GST-CNGC6 recombinant protein. In in vitro sumoylation assays, GST-CNGC6 was sumoylated by SSV2, depending on E1 and E2 activities (Fig. 7B). SSV2 self-sumoylation was also detected in the reaction products (Fig. 7C). However, sumoylated GST-CNGC6 was not detected in the reaction containing HPY2 instead of SSV2, while HPY2 self-sumoylation was clearly detected in the reaction products (Fig. 7, D and E), indicating that CNGC6 was specifically modified through the addition of the SUMO moiety by the E3 SUMO ligase activity of SSV2.

**Figure 8.**
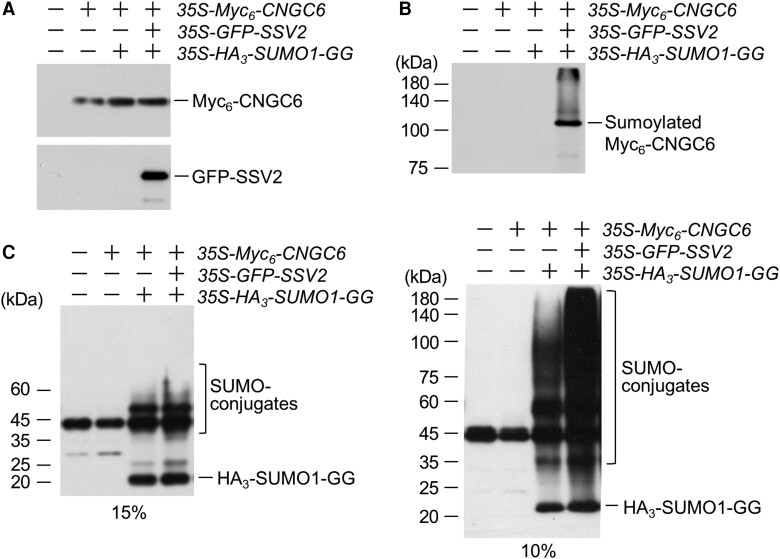
SSV2 sumoylates CNGC6 in vivo. Sumoylation of CNGC6 was analyzed in plants. *N. benthamiana* was infiltrated with or without *35S-Myc_6_-CNGC6*, *35S-GFP-SSV2*, and *35S-HA_3_-SUMO1-GG* as indicated. After incubation for 3 d, Myc_6_-CNGC6 and GFP-SSV2 were detected by immunoblot analysis with anti-Myc and anti-AtSIZ1 antibodies, respectively **(A)**. Sumoylated Myc_6_-CNGC6 was detected by immunoblot analysis with anti-Myc antibody following IP with anti-HA antibody **(B)**. For the detection of HA_3_-SUMO1-GG and its conjugates, immunoprecipitated samples were separated by 15% (left) or 10% (right) SDS–PAGE and then detected by western blot analysis with anti-HA antibody **(C)**.

To test whether SSV2 can function as an E3 SUMO ligase for CNGC6 in plants, we coinfiltrated *35S-Myc_6_-CNGC6*, *35S-GFP-SSV2*, and *35S-HA_3_-AtSUMO1-GG* constructs into the leaves of *N. benthamiana*. The expression of the recombinant proteins, Myc_6_-CNGC6 and GFP-SSV2, was detected by immunoblotting using anti-Myc and anti-AtSIZ1 antibodies, respectively (Fig. 8A). We examined the sumoylation of CNGC6 by immunoblotting with anti-Myc antibody following immunoprecipitation (IP) with anti-HA antibody. Importantly, we observed sumoylated CNGC6 only in the sample infiltrated with *35S-GFP-SSV2* (Fig. 8B). We also examined *HA_3_-AtSUMO1-GG* and its conjugates by western blotting using anti-HA antibody after IP. They were detected in the samples infiltrated with either the combination of *35S-Myc_6_-CNGC6* and *35S-HA_3_-AtSUMO1-GG* or *35S-Myc_6_-CNGC6*, *35S-GFP-SSV2*, and *35S-HA_3_-AtSUMO1-GG* constructs (Fig. 8C). The level of SUMO conjugates was relatively higher in the sample infiltrated with *35S-Myc_6_-CNGC6*, *35S-GFP-SSV2*, and *35S-HA_3_-AtSUMO1-GG* constructs compared to the sample infiltrated with *35S-Myc_6_-CNGC6* and *35S-HA_3_-AtSUMO1-GG* constructs (Fig. 8C).

### CNGC6 is stabilized by SSV2

**Figure 9.**
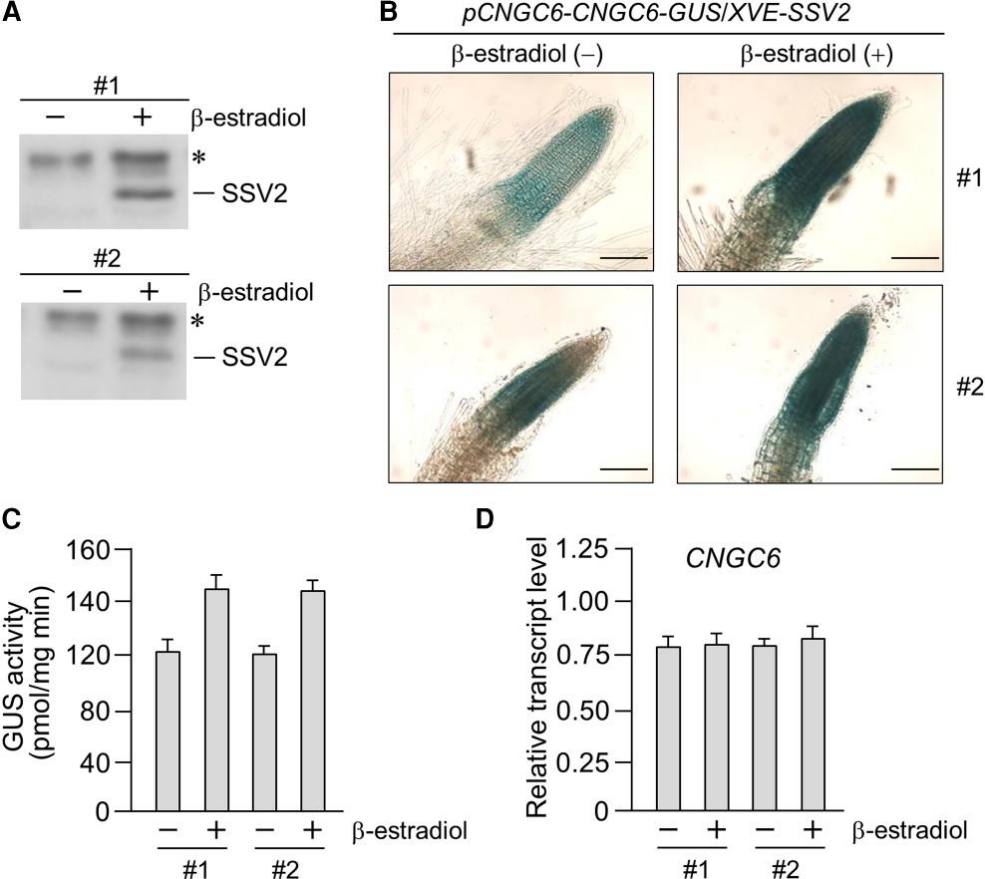
CNGC6 is stabilized by SSV2 in vivo. **A)** Transgenic plants harboring *pCNGC6-CNGC6-GUS*/*XVE-SSV2* were treated with β-estradiol for the induction of *SSV2* expression. After incubation for 15 h, total protein was isolated from transgenic plants, and the level of SSV2 was evaluated by immunoblot analysis with anti-SIZ1 antibody. Asterisks indicate nonspecific bands. **B)** GUS staining assay of transgenic plants treated with β-estradiol for 15 h. Scale bar1: 100 *µ*m. **C)** GUS enzyme activity was determined by a fluorescent method after β-estradiol treatment. Total proteins extracted from the roots of transgenic plants treated with β-estradiol were used for determination of enzyme activity. Error bars indicate standard deviations (Sd, *n* = 3). **D)** Analysis of the transcript level of *CNGC6-GUS* in β-estradiol-treated transgenic plants by RT-qPCR. Error bars indicate standard deviations (Sd, *n* = 3).

The direct binding of CNGC6 to SSV2 and the sumoylation of CNGC6 by SSV2 suggest that SSV2 regulates the stability or activity of CNGC6. To test whether SSV2 functions as an E3 SUMO ligase for CNGC6 in plants, we evaluated the effect of SSV2 on CNGC6 stability in vivo. The *pCNGC6-CNGC6-GUS* and *XVE-SSV2* plasmids were cointroduced into Arabidopsis plants, and two independent lines (#1 and #2) of the transgenic plants were analyzed. The level of CNGC6 was examined in transgenic lines after treatment with β-estradiol to induce the expression of *SSV2*, which was driven by *XVE*, a β-estradiol-inducible promoter (Zuo et al. 2000). Histochemical GUS staining and enzyme assays showed an increase in GUS activity in both transgenic lines following the induction of *SSV2* expression (Fig. 9, A to C). Because transcript levels of *CNGC6-GUS* can potentially affect the level of the CNGC6-GUS recombinant protein, we examined the *CNGC6* transcript levels in β-estradiol-treated transgenic plants by RT-qPCR. The results showed that transcript levels of *CNGC6* were comparable under these conditions (Fig. 9D), suggesting that the increase in GUS activity was caused by the SSV2-mediated stabilization of CNGC6.

To further validate the role of SSV2 in stabilizing CNGC6, we conducted an experiment to examine the impact of SSV2 on the decay of CNGC6. To do this, we generated double transgenic plants expressing either *35S-Myc_6_-CNGC6* and *XVE-HA_3_-SSV2* or *35S-Myc_6_-CNGC6* and *XVE-HA_3_-mSSV2*. Following the induction of SSV2 or mSSV2 expression through β-estradiol treatment, the plants were subsequently treated with cycloheximide (CHX) to inhibit new protein synthesis, and we then investigated the decay rate of Myc_6_-CNGC6. The results revealed that the degradation of Myc_6_-CNGC6 was delayed in SSV-expressing plants compared to nontreated plants (Supplementary Fig. S8A). However, the degradation rate of Myc_6_-CNGC6 remained similar in both nontreated and mSSV2-expressing plants (Supplementary Fig. S8B).

### *SSV2*-expressing transgenic *siz1-2* plants are tolerant to heat stress

**Figure 10.**
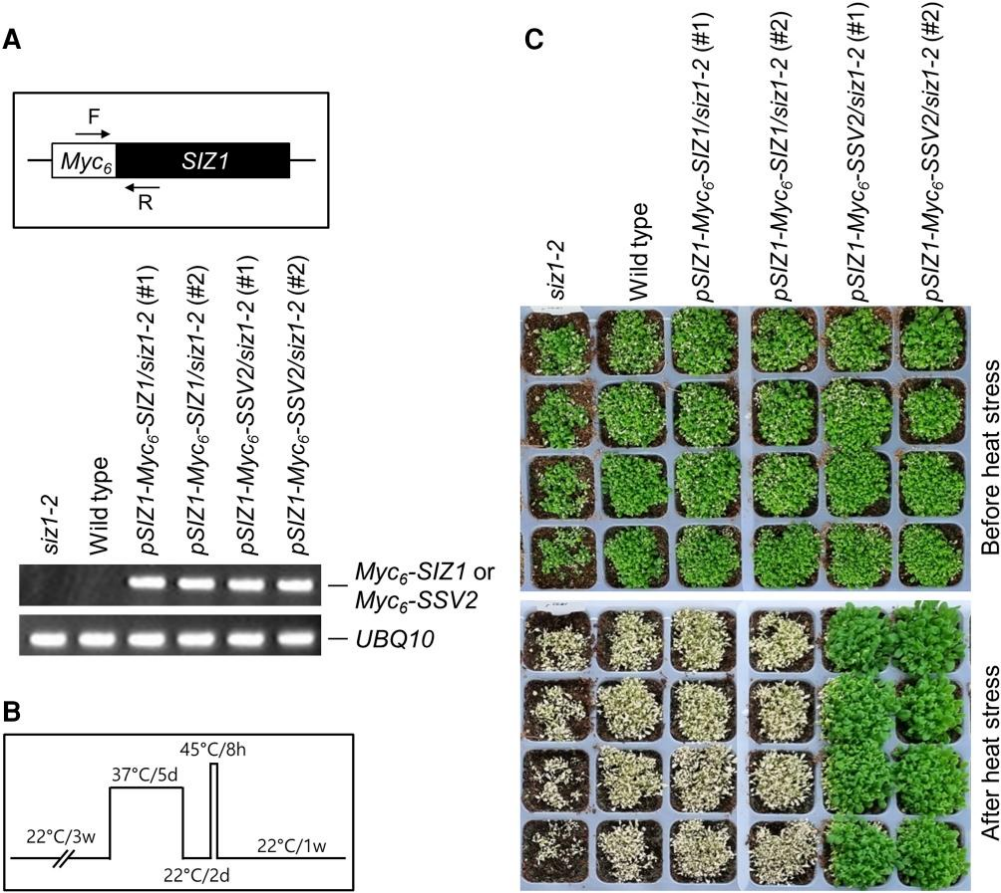
Transgenic *siz1-2* plants expressing *SSV2* under the control of the *SIZ1* promoter are tolerant to heat stress. Three-week-old WT, *siz1-2*, and *pSIZ1-Myc_6_-SIZ1*/*siz1-2*, and *pSIZ1-Myc_6_-SSV2*/*siz1-2* plants germinated and grown in soil were subjected to heat shock. **A)** Examination of *Myc_6_-SIZ1* and *Myc_6_-SSV2* expression in *pSIZ1-Myc_6_-SIZ1*/*siz1-2* and *pSIZ1-Myc_6_-SSV2*/*siz1-2* plants. Total RNA was extracted from the leaves of 3-wk-old WT, *siz1-2*, *pSIZ1-Myc_6_-SIZ1*/*siz1-2*, and *pSIZ1-Myc_6_-SSV2*/*siz1-2* plants. *Myc_6_-SIZ1* and *Myc_6_-SSV2* transcripts were examined by RT-PCR using *Myc*- and *SIZ1*-specific primers. F, *Myc*-specific forward primer; R, *SIZ1*-specific reverse primer. The *UBQ10* gene was used as a control. **B)** Schematic representation of the heat shock treatment used in this study. d, day; w, week. **C)** Photographs of plants taken 7 d after the heat treatment.

The accumulation of *SSV2/SSV5* transcripts under heat stress conditions and the stabilization of CNGC6 in the presence of SSV2 strongly indicate the involvement of SSV2 in the heat stress response. To examine whether SSV2 contributes to heat stress tolerance, we first produced transgenic *siz1-2* mutants expressing *SIZ1* or *SSV2* under the control of the *SIZ1* promoter. The *pSIZ1-Myc_6_-SIZ1* and *pSIZ1-Myc_6_-SSV2* constructs were separately introduced into the *siz1-2* mutant, and two independent lines were recovered from the transformation of each construct by RT-PCR (Fig. 10A). Then, we subjected 3-wk-old WT, *siz1-2*, *pSIZ1-Myc_6_-SIZ1/siz1-2*, and *pSIZ1-Myc_6_-SSV2/siz1-2* plants to heat stress (Fig. 10B). Results showed that *pSIZ1-Myc_6_-SSV2/siz1-2* plants were more tolerant to heat stress than WT, *siz1-2*, and *pSIZ1-Myc_6_-SIZ1/siz1-2* plants (Fig. 10C).

We also produced transgenic *siz1-2* mutants expressing *SIZ1* or *SSV2* under control of the *35S* promoter by transforming *siz1-2* mutants with *35S-Myc_6_-SIZ1* or *35S-Myc_6_-SSV2*, respectively. Two independent lines of transgenic *siz1-2* plants expressing the *Myc_6_-SIZ1* transgene, *35S-Myc_6_-SIZ1/siz1-2* (#1) and *35S-Myc_6_-SIZ1/siz1-2* (#2), and those of transgenic *siz1-2* plants expressing the *Myc_6_-SSV2* transgene, *35S-Myc_6_-SSV2/siz1-2* (#1) and *35S-Myc_6_-SSV2/siz1-2* (#2), were identified by immunoblotting analysis (Supplementary Fig. S9A). Next, we performed heat stress tolerance assays by exposing 3*-*wk-old WT, *siz1-2*, *35S-Myc_6_-SIZ1/siz1-2*, and *35S-Myc_6_-SSV2/siz1-2* plants to heat stress (Supplementary Fig. S9B). Results showed that *35S-Myc_6_-SSV2/siz1-2* plants were more tolerant to heat stress than WT, *siz1-2*, and *35S-Myc_6_-SIZ1/siz1-2* plants (Supplementary Fig. S9C).

**Figure 11.**
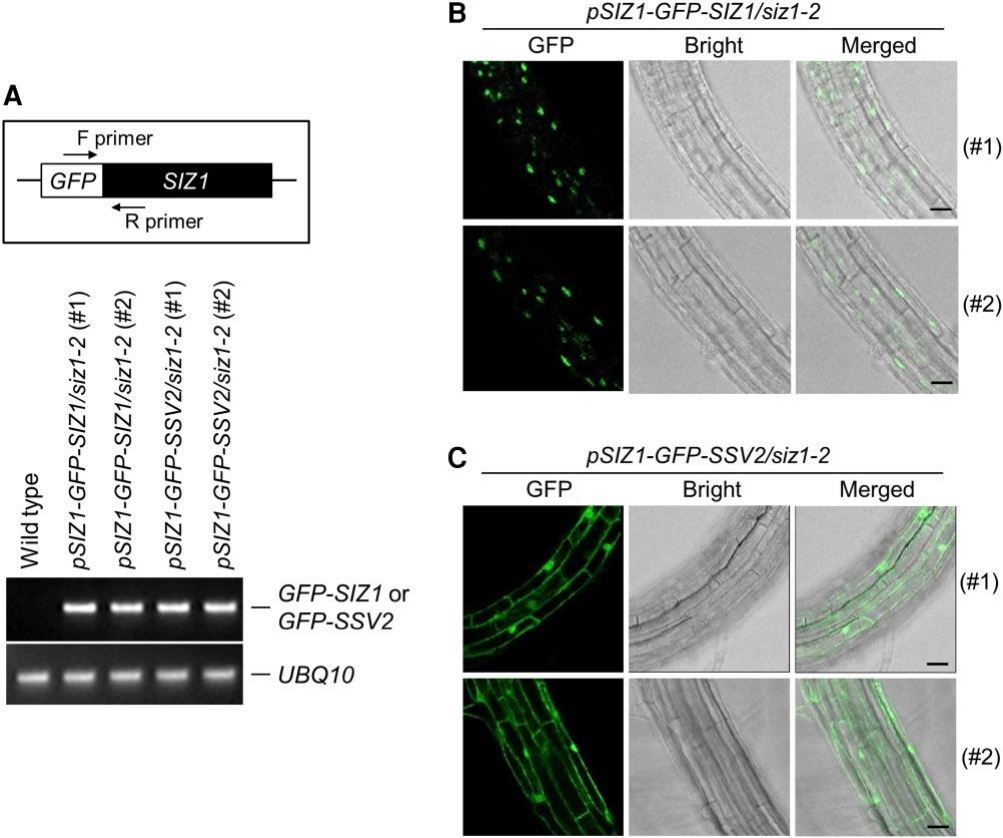
Generation of transgenic *siz1-2* plants expressing *SIZ1* promoter-driven *GFP-SSV2* and subcellular localization of GFP-SSV2 in Arabidopsis. **A)** Examination of *GFP-SIZ1* and *GFP-SSV2* expression in *pSIZ1-GFP-SIZ1*/*siz1-2* and *pSIZ1-GFP-SSV2*/*siz1-2* plants. Total RNA was extracted from the leaves of 3-wk-old WT, *pSIZ1-GFP-SIZ1*/*siz1-2*, and *pSIZ1-GFP-SSV2*/*siz1-2* plants. *GFP-SIZ1* and *GFP-SSV2* transcripts were examined by RT-PCR using *GFP*- and *SIZ1*-specific primers. F, *GFP*-specific forward primer; R, *SIZ1*-specific reverse primer. *UBQ10* was used as the control gene. **B, C)** Analysis of the subcellular localization of SIZ1 and SSV2 in Arabidopsis. Seeds of *pSIZ1-GFP-SIZ1*/*siz1-2* and *pSIZ1-GFP-SSV2*/*siz1-2* plants were germinated on MS media. After 7 d, the GFP signal was detected in roots by CLSM. Scale bars = 10 *µ*m.

Additionally, we analyzed the subcellular localization of SIZ1 and SSV2 in Arabidopsis. The *siz1-2* mutants were transformed with *pSIZ1-GFP-SIZ1* or *pSIZ1-GFP-SSV2*, and two independent transgenic lines expressing the *GFP-SIZ1* transgene, *pSIZ1-GFP-SIZ1*/*siz1-2* (#1) and *pSIZ1-GFP-SIZ1*/*siz1-2* (#2), and those expressing the *GFP-SSV2* transgene, *pSIZ1-GFP-SSV2/siz1-2* (#1) and *pSIZ1-GFP-SSV2/siz1-2* (#2), were identified by RT-PCR (Fig. 11A). Then, CLSM analysis was performed to detect GFP signals in the roots of transgenic plants. The fluorescence signal of GFP-SIZ1 was detected in the nucleus (Fig. 11B), while that of GFP-SSV2 was detected in both the cytoplasmic membrane and nucleus (Fig. 11C). Finally, to rule out the possibility of the effect of the Myc-tag on the heat stress tolerance of transgenic *siz1-2* plants, we applied heat stress to 3*-*wk-old WT, *pSIZ1-GFP-SIZ1*/*siz1-2*, and *pSIZ1-GFP-SSV2/siz1-2* transgenic plants. Expectedly, *pSIZ1-GFP-SSV2/siz1-2* plants were more tolerant to heat stress than WT and *pSIZ1-GFP-SIZ1*/*siz1-2* plants (Supplementary Fig. S10).

Increased heat tolerance of SSV2-expressing transgenic *siz1-2* plants suggests that the levels of SUMO-conjugates are increased or decreased in SSV2-expressing transgenic *siz1-2* plants compared to WT or *35S-Myc_6_-SIZ1/siz1-2* transgenic plants. Thus, we examined their levels in WT, *35S-Myc_6_-SIZ1/siz1-2* and *35S-Myc_6_-SSV2/siz1-2* transgenic plants under heat stress condition, as SUMO-conjugates were reported to accumulate under such condition (Saracco et al. 2007). After exposure of 3-wk-old WT, *35S-Myc_6_-SIZ1/siz1-2* and *35S-Myc_6_-SSV2/siz1-2* transgenic plants to heat stress, the levels of SUMO-conjugates were analyzed using immunoblot analysis. Results indicated that the levels of SUMO-conjugates were similar in WT, *35S-Myc_6_-SIZ1/siz1-2*, and *35S-Myc_6_-SSV2/siz1-2* transgenic plants, regardless of whether they were subjected to heat treatment or not (Supplementary Fig. S11).

### Identification of sumoylation site on CNGC6

Next, we attempted to identify the sumoylation site of CNGC6. The predicted amino acid sequences of CNGC6 revealed the presence of five potential sumoylation sites (ΨKXE) located at lysine 66 (K66), lysine 347 (K347), lysine 578 (K578), lysine 669 (K669), and lysine 675 (K675; Supplementary Fig. S12, A and B). To determine the specific sumoylation sites on the CNGC6 protein, we generated single mutant derivatives with the mutations K66R, K347R, K578R, K669R, and K675R. These mutant proteins were overexpressed in *Escherichia coli*, purified using glutathione affinity columns, and utilized for in vitro sumoylation assays. The results of the in vitro sumoylation assays demonstrated that GST-mCNGC6-HA (K66R), GST-mCNGC6-HA (K578R), GST-mCNGC6-HA (K669R), and GST-mCNGC6-HA (K675R) underwent sumoylation, while GST-mCNGC6-HA (K347R) did not (Supplementary Fig. S12C). Therefore, these findings suggest that K347 is the principal site of SUMO conjugation on CNGC6.

### CNGC6 modification by SUMO is necessary for heat tolerance

**Figure 12.**
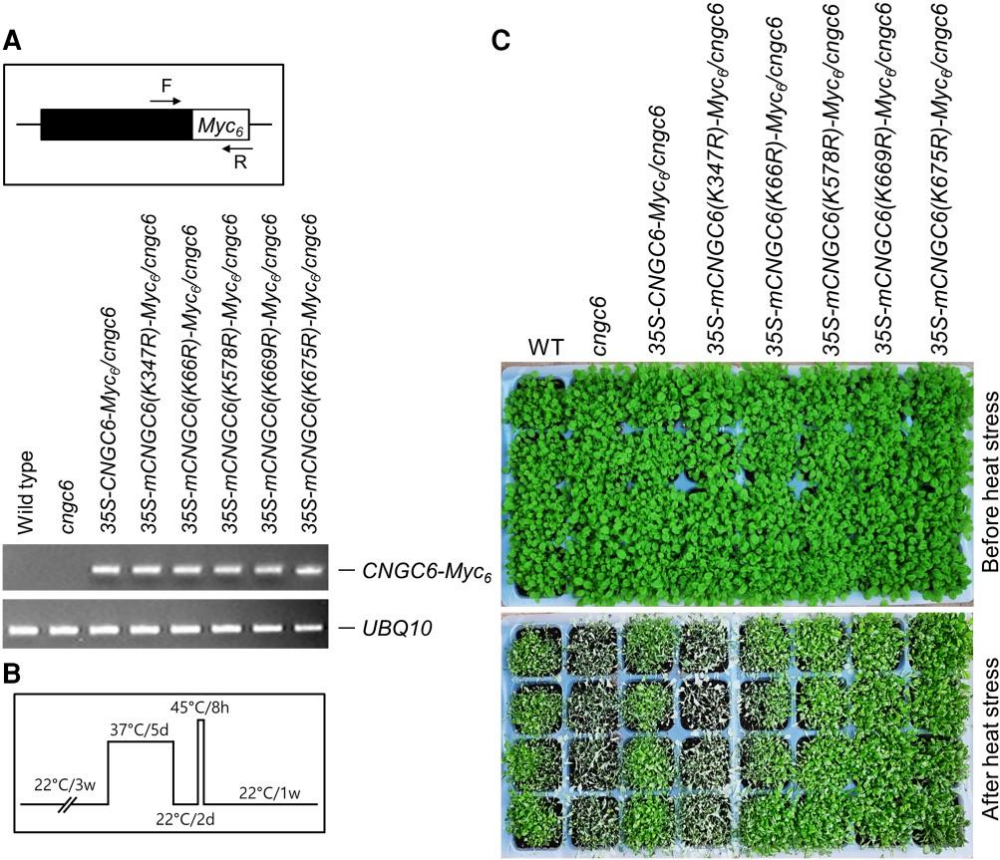
Sumoylation of CNGC6 is essential for heat tolerance. Three-week-old WT, cngc6, 35S-CNGC6-Myc_6_/cngc6, 35S-mCNGC6(K66R)-Myc_6_/cngc6, 35S-mCNGC6(K347R)-Myc_6_/cngc6, 35S-mCNGC6(K578R)-Myc_6_/cngc6, 35S-mCNGC6(K669)-Myc_6_/cngc6, and 35S-mCNGC6(675R)-Myc_6_/cngc6 were germinated and grown in soil and then subjected to heat shock. **A)** Examination of CNGC6-Myc_6_, mCNGC6(K66R)-Myc_6_, mCNGC6(K347R)-Myc_6_, mCNGC6(K578R)-Myc_6_, mCNGC6(K669R)-Myc_6_, and mCNGC6(K675R)-Myc_6_ expression in transgenic cngc6 plants. Total RNA was extracted from the leaves of 3-wk-old WT, cngc6, and transgenic cngc6 plants. CNGC6-Myc_6_, mCNGC6(K66R)-Myc_6_, mCNGC6(K347R)-Myc_6_, mCNGC6(K578R)-Myc_6_, mCNGC6(K669R)-Myc_6_, and mCNGC6(K675R)-Myc_6_ transcripts were examined by RT-PCR using CNGC6- and Myc-specific primers. F, CNGC6-specific forward primer; R, Myc-specific reverse primer. The UBQ10 gene was used as a control. **B)** Schematic representation of the heat shock treatment used in this study. d, day; w, week. **C)** Photographs of plants taken 5 d after the heat treatment.

Based on our sumoylation data, we conducted an investigation into the impact of sumoylation on the activity of CNGC6 as a mediator of heat tolerance. Transgenic *cngc6* plants overexpressing CNGC6-Myc_6_, mCNGC6(K66R)-Myc_6_, mCNGC6(K347R)-Myc_6_, mCNGC6(K578R)-Myc_6_, mCNGC6(K669R)-Myc_6_, and mCNGC6(K675R)-Myc_6_ using the corresponding constructs *35S-CNGC6-Myc_6_*, *35S-mCNGC6(K66R)-Myc_6_*, *35S-mCNGC6(K347R)-Myc_6_*, *35S-mCNGC6(K578R)-Myc_6_*, *35S-mCNGC6(K669)-Myc_6_*, and *35S-mCNGC6(675R)-Myc_6_* were generated. After the selection of homozygous lines, their expression was initially examined by RT-PCR (Fig. 12A). Subsequently, the transgenic plants were assessed for heat tolerance (Fig. 12B). Results revealed that transgenic *cngc6* plants overexpressing mCNGC6(K347R)-Myc_6_ were sensitive to heat stress, similar to the *cngc6* mutants (Fig. 12C). However, other transgenic *cngc6* plants overexpressing CNGC6-Myc_6_, mCNGC6(K66R)-Myc_6_, mCNGC6(K578R)-Myc_6_, mCNGC6(K669R)-Myc_6_, and mCNGC6(K675R)-Myc_6_ exhibited restored heat tolerance to similar to WT (Fig. 12C), indicating that sumoylation is necessary for the role of CNGC6 in heat tolerance.

### Putative AtSIZ1 orthologs in plants and animals

Splicing variants of *AtSIZ1* in Arabidopsis suggest that splicing variants may also exist for its orthologs in other eukaryotic systems. Therefore, we examined the splicing variants of AtSIZ1 orthologs in other plants and animals (Supplementary Fig. S13, A to G). First, we searched for the splicing variants of AtSIZ1 orthologs in rice, Maize (*Zea mays*), soybean (*Glycine max*), and tomato using available databases. The results showed that, unlike AtSIZ1, rice OsSIZ1 and OsSIZ2 contained two NLS motifs located in the N-terminal and C-terminal regions, and the N-terminal NLS motif was deleted in the splicing variant OsSIZ1.3. In maize, an NLS motif was located in the N-terminal part of ZmSIZ1a, ZmSIZ1b, and ZmSIZ1c, but it was not present in the splicing variant ZmSIZ1c.2. Similarly, in soybean, GmSIZa and GmSIZb also contained two NLS motifs located in the N-terminal and C-terminal regions, and the C-terminal NLS motif was absent in the splicing variant GmSIZa.2. Tomato SISIZa and SISIZb also contained two NLS motifs in the N-terminal and C-terminal regions. However, the splicing variant SISIZ1b.2 did not contain the N-terminal NLS motif.

Second, we also analyzed the splicing variants of AtSIZ1 orthologs in human (*Homo sapiens*) and mouse (*Mus musculus*). In human, there were four AtSIZ1 orthologs: HsPIAS1.1, HsPIAS2.1, HsPIAS3.1, and HsPIAS4.1, and five different splicing variants: HsPIAS1.2, HsPIAS2.2, HsPIAS2.3, HsPIAS3.2, and HsPIAS4.2. Among the splicing variants, HsPIAS2.3 did not contain the NLS motif. In mouse, there were four AtSIZ1 orthologs: MmPIAS1.1, MmPIAS2.1, MmPIAS3.1, and MmPIAS4.1, and six different splicing variants: MmPIAS1.2, MmPIAS2.2, MmPIAS2.3, MmPIAS3.2, MmPIAS3.3, and MmPIAS4.2. However, the NLS motif-deleted splicing variant was not found in the current database. An extended search of AtSIZ1 orthologs in yeast showed that there were two AtSIZ1 orthologs, ScSIZ1 and ScSIZ2, but their splicing variants were not present in the current database.

Further detailed analysis of the splicing variants revealed that the SAP domain, as well as the N-terminal NLS motif, was also absent in the splicing variants OsSIZ1.3 and SISIZ1b.2. Additionally, the SAP domain and PINIT motif, as well as the NLS motif, were not present in the splicing variant HsPIAS2.3. Moreover, the S/T-rich motif was absent in the splicing variants HsPIAS4.1, MmPIAS1.2, and MmPIAS2.2. In the case of splicing variants HsPIAS4.2 and MmPIAS4.2, both the PINIT and S/T-rich motifs were not present.

## Discussion

Alternative splicing regulates diverse physiological processes in plants and animals, including growth, environmental adaptation, and stress response (Kuang et al. 2017; Chen et al. 2019b). In plants, alternative splicing occurs at an unexpectedly high frequency (Filichkin et al. 2010; Lu et al. 2010; Marquez et al. 2012; Syed et al. 2012). Recent high-throughput RNA-seq analyses in plants show that alternative splicing is pervasive across plant species, with more than 80% and 70% of intron-containing genes producing different mRNA isoforms in Arabidopsis and rice, respectively (Zhu et al. 2017; Chen et al. 2019a).

Four alternatively spliced variants of Arabidopsis *SIZ1* (*SSV1 to SSV4*; Fig. 1A) were reported two decades ago; however, none of these variants have been characterized to date. Therefore, in this study, we analyzed these *SSVs* on the basis of data generated by computer programs. These data suggested that alternative splicing occurred at the 3'-end of the *SIZ1* transcript, causing the production of four splicing variants (Fig. 1, A and B). Then, we characterized the molecular and biochemical properties of these splicing variants in comparison with those of *SIZ1*. We first examined their subcellular localization of GFP-labeled SIZ1 and SSV1-3 using *N. benthamiana*. Interestingly, SIZ1, SSV1, and SSV3 proteins localized to the nucleus, whereas SSV2 localized mainly to the plasma membrane and to some extent to the nucleus (Figs. 2 and 11 and Supplementary Fig. S2C). Further investigation of the subcellular localization of N- and C-terminal deletion mutant proteins of SIZ1 revealed that the removal of various domains (SAP, PHD, PINIT, SP-RING, and SXS) of SIZ1, except the NLS, had no effect on its subcellular localization (Fig. 3). Considering the lack of the NLS motif, we predicted that SSV2 localizes to the plasma membrane. However, our results showed that SSV2 also localized to the nucleus to some extent (Fig. 2D, 3B, and 11C and Supplementary Fig. S2C). These results suggest that the subcellular localization of SIZ1 is affected not only by the C-terminal NLS motif but also by unknown carrier protein(s) interacting with SIZ1.

Because SIZ1 and all SSVs possess conserved motifs, except the lack of the NLS motif in SSV2, and the presence of specific C-terminal peptides in SSV1, SSV3, and SSV4 (Fig. 1, A and B), we speculated that SIZ1 and all SSVs exhibit E3 SUMO ligase activity, albeit with minor differences in activity strength. SIZ1, SSV1, and SSV3 exhibit self-sumoylation to similar extents (Fig. 4, A to D), despite the weaker interaction of SSV1 with SUMO proteins and that of SSV3 with SUMO proteins (Supplementary Fig. S3, A to E). More interestingly, SIZ1 and all SSVs were self-sumoylated in the presence of SUMO1 or SUMO2 but not in the presence of SUMO3 and SUMO5 (Fig. 4, A to D). Additionally, HPY2 also exhibited self-sumoylation in a similar manner to that of SIZ1 (Fig. 4E). Altogether, these data suggest that the C-terminal region of SIZ1 is involved in its nuclear localization but is not required for its E3 ligase activity, and that SP-RING finger-type E3 SUMO ligases such as SIZ1 and HPY2 prefer SUMO1 and SUMO2 for their E3 ligase activity.

In the animal system, one splicing variant for E3 SUMO ligase PIASy (Protein inhibitor of activated STAT Y), a member of the PIAS family, has been reported previously (Wong et al. 2004). The authors showed that both PIASy and a PINIT motif-deleted splicing variant localized to the nucleus; however, cells expressing the splicing variant showed reduced sumoylation of target proteins compared with cells expressing the full-length PIASy (Wong et al. 2004). These results also suggest that the N-terminal region of PIASy is necessary for its E3 ligase activity but not responsible for its localization. Therefore, based on the results of the current study and those of previous studies, we carefully infer that the localization of SIZ1, SSV1, SSV2, and SSV3 is determined mainly by their C-terminal regions. We also speculate that these proteins can function as E3 SUMO ligases in different subcellular organelles, although the data presented in this study show that SIZ1 and SSVs localize either to the nucleus or to the plasma membrane.

Different subcellular localization patterns of SIZ1 and SSV2 suggest that their transcript levels also can be influenced or controlled by the plant developmental stage and environmental conditions. RT-PCR analyses using two sequence-specific primer sets showed that the transcript level of *SIZ1* was much higher than those of its splicing variants *SSV1*, *SSV2*, *SSV3*, and *SSV4* under normal conditions as well as after treatment with various abiotic stresses, hormones, and nitrogen sources (Supplementary Fig. S5). In addition, transcript levels of *SIZ1*, *SSV1*, *SSV3*, and *SSV4* were not greatly affected by abiotic stresses, hormones, and nitrogen sources (Supplementary Fig. S5). However, the transcript level of *SSV2* increased substantially (∼20-fold) after the heat stress treatment (Fig. 5B). These data indicate that *SSV2* transcripts are specifically produced and accumulated under heat stress, suggesting that the *SSV2*-encoded protein mainly functions as an E3 SUMO ligase in the plasma membrane under the heat stress condition. More interestingly, a novel PCR product, not predicted by the database, was clearly detected under the heat stress condition (Fig. 6B; Supplementary Fig. S5B, lower panel). Analysis of the nucleotide sequence of this PCR product revealed that it represented a novel splicing variant of *SIZ1*, which we named as *SSV5* (Supplementary Fig. S6). However, notably, the deduced amino acid sequence of SSV5 (832 aa) was identical to that of SSV2 (Supplementary Fig. S7). Together, these results strongly suggest that SSV2 protein, which lacks the NLS peptide, is specifically accumulated heat stress, and given their E3 ligase activities, SSV2 can sumoylate plasma membrane proteins or plasma membrane-localized proteins to modulate their activity or stability under heat stress. Besides, compared with SSV transcript levels, the transcript level of *SIZ1* was the highest under heat stress (Fig. 5), suggesting that the SIZ1 protein can also regulate the stability and activity of its target proteins in the nucleus under heat stress.

Previous studies reported that a large number of proteins undergo sumoylation in response to heat stress, and the level of SUMO-conjugates is reduced in the *siz1* mutant at high temperature (Saracco et al. 2007; Miller et al. 2010; Rytz et al. 2018). These findings suggest that SIZ1 is involved in the sumoylation of its target substrates under heat stress to regulate their stability and activity. In plants, one of the responses to increased ambient temperatures is a transient influx of Ca^2+^ from the extracellular matrix into the cytoplasm (Gong et al. 1998; Liu et al. 2006). In addition, the repression of the heat shock response pathway through Ca^2+^ chelating and channel blocking indicates that rate of Ca^2+^ influx and subsequent signaling are critical for the heat stress response (Braam 1992; Larkindale and Knight 2002; Li et al. 2004; Suri and Dhindsa 2008). CNGC6 mediates the heat-activated Ca^2+^ influx and the expression of *HSP* genes in Arabidopsis during adaption to high temperature (Niu and Xiang 2018; Peng et al. 2019). In the current study, analysis of the effect of SSV2 on CNGC6 stability showed that SSV2 sumoylated and increased the level of CNGC6 through direct interaction in vitro and in vivo (Figs. 7 to 9; Supplementary Fig. S8). These data indicate that SSV2 stabilizes CNGC6 through direct interaction and its E3 SUMO ligase activity, and plays a positive role in CNGC6-mediated heat tolerance.

Alternative splicing patterns vary greatly under different environmental stresses, which allows plants to rapidly adjust the abundance and function of crucial stress-responsive proteins (Laloum et al. 2018; Rigo et al. 2019). Recently, heat stress priming-induced alternative memory was observed in Arabidopsis plants, which helps the plants survive subsequent lethal stress events by generating appropriate stress-responsive splice isoforms (Ling et al. 2018 and 2021). Alternative splicing can effectively downregulate gene expression by creating truncated protein isoforms (Kelemen et al. 2013). Alternative splicing also influences the regulatory properties of alternative translation products by modulating their subcellular localization (Teardo et al. 2015; Cecchini et al. 2022). The heat stress-specific accumulation of SSV2, stabilization of CNGC6 through the E3 SUMO ligase activity of SSV2, and plasma membrane localization of SSV2 led us to investigate the role of SSV2 in heat stress in plants. Heat tolerance analyses of transgenic *siz1-2* plants expressing *SIZ1* or *SSV2* under control of *SIZ1* or *35S* promoter showed that *SSV2*-expressing transgenic *siz1-2* plants were more tolerant to heat stress than WT plants; however, the heat tolerance of *SIZ1*-expressing transgenic *siz1-2* plants was similar to that of WT plants (Fig. 10; Supplementary Figs. S9 and S10). These results indicate that, among SIZ1 and its splicing variants, SSV2 is the main regulator of heat stress tolerance.

The production of *SSV2* and *SSV5* transcripts was induced in WT plants under heat stress conditions, which contributes to the heat tolerance observed in WT plants. In addition, SSV2 protein was substantially higher in *pSIZ1-Myc_6_-SSV2/siz1-2* or *35S-Myc_6_-SSV2/siz1-2* plants compared to WT plants, both under normal conditions and even more so under heat stress conditions. This increased production of SSV2 protein resulted in enhanced tolerance in the *pSIZ1-Myc_6_-SSV2/siz1-2* or *35S-Myc_6_-SSV2/siz1-2* plants compared to WT plants. Hence, the observed improvements in heat tolerance phenotypes in the SSV2-expressing transgenic *siz1-2* plants, regulated by either the *SIZ1* or *35S* promoter, compared to *siz1-2*, WT, and SIZ1-expressing plants, could potentially be attributed to the gain-of-function effect resulting from SSV2 overexpression.

Despite the increased heat tolerance observed in SSV2-expressing transgenic *siz1-2* plants, there was no difference in the levels of sumoylated proteins among WT and *siz1-2* plants expressing SIZ1 or SSV2 (Supplementary Fig. S11). Due to the localization of SSV2 and SIZ1, SSV2 can sumoylate its target proteins distributed in the plasma membrane, while SIZ1 cannot influence the sumoylation of membrane-anchored or localized proteins. SSV2 is also partially localized in the nucleus, suggesting that nuclear-targeted proteins can be sumoylated in SSV2-expressing *siz1-2* plants. Consequently, sumoylated proteins in SSV2-expressing *siz1-2* plants may differ from those in SIZ1-expressing *siz1-2* plants, although these differences cannot be distinguished in immunoblot data. Additionally, sumoylated proteins in the WT may also differ from those in SIZ1-expressing *siz1-2* or SSV2-expressing *siz1-2* plants due to the higher levels of SIZ1 or SSV2 in the transgenic plants, even though these differences are not apparent in the immunoblot data. Therefore, we cautiously infer that the sumoylation levels of SIZ1- or SSV2-specific target proteins may vary in specific subcellular regions, despite the similar levels of total SUMO-conjugates detected by immunoblotting in WT and SIZ1- or SSV2-expressing *siz1-2* plants.

Previous studies have reported that mutations in sumoylation sites lead to the loss of protein function. For instance, mutations in the sumoylation sites of SLY1 and FLC proteins disrupted GA signaling (Kim et al. 2015b) and abolished the flowering repression activity of FLC (Son et al. 2014), respectively. Therefore, we extended our study to investigate the impact of sumoylation on the activity of the CNGC6 protein. After identifying the sumoylation site (K347) of CNGC6 (Supplementary Fig. S12), we analyzed the heat tolerance of transgenic *cngc6* plants expressing a mutant CNGC6 protein (K347R). The results clearly showed that these plants were sensitive to heat stress, similar to the level observed in *cngc6* mutants (Fig. 12), indicating that sumoylation of CNGC6 by SSV2 is essential for CNGC6-mediated heat tolerance.

SIZ/PIAS-type E3 SUMO ligases are commonly found in eukaryotic systems, including plants and animals, indicating that the sumoylation of target proteins by their activity is an important mechanism for growth and development. Extended analysis of SIZ/PIAS-type E3 SUMO ligases in rice, maize, soybean, tomato, human, mouse, and yeast revealed the presence of various splicing variants (Supplementary Fig. S13). It is known that the SAP domain is involved in sequence- or structure-specific DNA binding (Aravind and Koonin 2000), and the NLS and PINIT motifs are involved in nuclear retention (Duval et al. 2003). The S/T-rich motif only exists in vertebrates, and its function remains to be defined. Therefore, the existence of different types of splicing variants of SIZ/PIAS-type E3 SUMO ligases in plant and animal systems suggests that their subcellular localization and role are modulated by alternative splicing. This also implies that alternative splicing is a crucial mechanism contributing to their functional diversity.

In conclusion, we found that *SIZ1* splicing variants *SSV2* and *SSV5* were highly upregulated in Arabidopsis under heat stress. Proteins encoded by *SSV2* and *SSV5* were identical and mainly localized to the plasma membrane, with some localization to the nucleus. In addition, SSV2 directly bound to and sumoylated CNGC6 through its E3 SUMO ligase activity, thus improving CNGC6 stability. Moreover, SSV2 expression induced heat stress tolerance and the sumoylation site mutation of CNGC6 caused the loss of its function in heat tolerance. This study shows that splicing variants of the E3 SUMO ligase-encoding gene *SIZ1* are specifically accumulated under certain growth conditions, and proteins encoded by these splicing variants function in different subcellular compartments. Because SIZ/PIAS-type E3 SUMO ligases are found in all eukaryotes including plants and animals, our findings will help elucidate the role of these enzymes in mediating diverse cellular events in different subcellular organelles of plants during their growth and development under normal and stress conditions.

## Materials and methods

### Plant material and growth conditions

Arabidopsis (*A. thaliana*) ecotype Columbia-0 (Col-0) was used as the WT in this study. Seeds were surface-sterilized with a solution containing 5% (w/v) sodium hypochlorite and 0.1% Triton X-100 for 10 min, and then rinsed three times with sterilized water. The sterilized seeds were sown either on Murashige and Skoog (MS) medium (pH 5.7) containing 2% (w/v) sucrose and 0.8% (w/v) agar, or directly in sterile vermiculite. All plants, including seedlings, were grown in an environmentally controlled growth chamber at 22 °C and 16 h light/8 h dark cycle. The experiments were repeated three times unless otherwise mentioned.

### Plasmid construction

To produce MBP-SIZ1, MBP-SSV1, MBP-SSV2, MBP-SSV3, MBP-SSV3-D, and MBP-HYPLODY 2 (HPY2) recombinant proteins, the cDNA sequences of *SIZ1*, *SSVs*, and *HPY2* were amplified by PCR using gene-specific primers and cloned into the pMALc2 vector (New England Biolabs). To generate His_6_-AtSUMO1-GG, His_6_-AtSUMO2-GG, His_6_-AtSUMO3-GG, and His_6_-AtSUMO5-GG, full-length cDNAs were amplified by PCR with gene-specific primers and cloned into the pET28a vector (Novagen). To produce glutathione S-transferase (GST)-CNGC6, full-length *CNGC6* cDNA was amplified by PCR with gene-specific primers and cloned into the pGEX4T-1 vector (Amersham Biosciences). In addition, to produce GST-CNGC6-HA, the cDNA encoding the full-length CNGC6 was amplified by PCR using primers tagged with HA and inserted into pGEX4T-1 plasmid. For the production of CNGC6 mutant proteins GST-mCNGC6(K65R)-HA, GST-mCNGC6(K347R)-HA, GST-mCNGC6(K578R)-HA, GST-mCNGC6(K669R)-HA, and GST-mCNGC6(K675R)-HA (the numbers indicate the positions of the lysines in CNGC6 that were mutated to arginine), site-directed mutagenesis was performed on the GST-CNGC6-HA construct using overlapping primers. All constructs were transformed into *E. coli* BL21/DE3 (pLysS) cells. The transformed *E. coli* cells were cultured in liquid LB media, and 5 mM isopropyl-β-D-thiogalactoside was added to the culture to induce protein expression. All primer sequences used in this study are described in Supplementary Table S1.

### Recombinant protein purification

All recombinant proteins produced in *E. coli* BL21 cells were purified according to the manufacturer's instructions. Briefly, to extract His_6_-AtSAE1b, His_6_-AtSAE2, His_6_-AtSCE1, His_6_-AtSUMO1-GG, His_6_-AtSUMO2-GG, His_6_-AtSUMO3-GG, and His_6_-AtSUMO5-GG recombinant proteins, bacteria were lysed in 50 mM NaH_2_PO_4_ (pH 8.0), 300 mM NaCl, 1% (v/v) Triton X-100, 1 mM imidazole, 5 mM dithiothreitol, 2 mM phenylmethylsulfonyl fluoride (PMSF), and protease inhibitor cocktail. The recombinant proteins were purified using Ni^2+^-nitrilotriacetate (NTA) resins (Qiagen).

To extract MBP, MBP-SIZ1, MBP-SSV1, MBP-SSV2, MBP-SSV3, MBP-SSV3-D, and MBP-HPY2 proteins, bacteria were lysed in 20 mM Tris (pH 7.4), 200 mM NaCl, 1 mM ethylenediaminetetraacetic acid (EDTA), 1% (v/v) Triton X-100, 2 mM PMSF, and proteinase inhibitor cocktail (Roche). The recombinant proteins were purified using amylose resins (New England BioLabs).

Bacteria expressing GST-CNGC6, GST-mCNGC6(K65R)-HA, GST-mCNGC6(K347R)-HA, GST-mCNGC6(K578R)-HA, GST-mCNGC6(K669R)-HA, and GST-mCNGC6(K675R)-HA were lysed in phosphate-buffered saline (PBS; pH 7.5) containing 1% (v/v) Triton X-100, 2 mM PMSF, and proteinase inhibitor cocktail (Roche). The recombinant protein was purified using glutathione resins (Pharmacia).

### Y2H assays

Y2H assays were performed using the Gal4-based two-hybrid system (Clontech). Full-length cDNAs of *SIZ1, SSV1, SSV2, SSV3, and SSV4* as well as the cDNA fragment encoding 1 to 858 amino acids (aa) of SSV3 were cloned into the activating domain (AD)-containing pGAD424 vector to generate *AD-SIZ1, AD-SSV1, AD-SSV2, AD-SSV3*, *AD-SSV4*, and *AD-SSV3-D* constructs, respectively. Additionally, *AtSCE1, AtSUMO1, AtSUMO2, AtSUMO3*, and *AtSUMO5* cDNAs were cloned into the binding domain (BD)-containing pGBT8 vector to generate the *BD-AtSCE1*, *BD-AtSUMO1*, *BD-AtSUMO2*, *BD-AtSUMO3*, and *BD-AtSUMO5* constructs, respectively. All constructs were transformed into yeast (*S. cerevisiae*) strain AH109 using the lithium acetate method. Yeast cells were grown on minimal medium (−Leu/–Trp). Transformants were plated on –Leu/–Trp/–His minimal medium containing 1 or 5 mM 3-amino-1,2,4-triazole (3-AT) to test the interactions of SIZ1 and SSVs with AtSCE1, AtSUMO1-3, and AtSUMO5.

### Stress treatments, and *SIZ1* and *SSV* expression analysis

The effects of various abiotic stresses, phytohormones, and nitrogen sources on *SIZ1* and *SSV* transcript levels were analyzed using 10-d-old seedlings grown on MS media. To test the effects of abiotic stresses, seedlings were treated with high temperature (37 °C) for 0, 0.5, 1 or 2 h; low temperature (4 °C) for 4 h; drought stress for 4 h; and salt stress (100 mM NaCl) for 4 h. Seedlings were also treated for 4 h with various phytohormones, including 5 *μ*M NAA, 5 *μ*M 1-N-naphthylphthalamic acid (NPA), 10 *μ*M GA_3_, 5 *μ*M ABA, and 5 *μ*M MeJA, and nitrogen sources, including 5 mM KNO_3_ and 5 mM (NH_4_)_2_SO_4_. Relative transcript levels of *SIZ1* and *SSVs* were also analyzed using 5-d-old whole seedlings, rosette leaves, and stems of 3-wk-old plants, immature siliques, and mature seeds.

Total RNA was extracted from the ground tissue using the FavorPrep Plant Total RNA Mini Kit (Favorgen). The isolated total RNA was treated with gDNA Remover (TOYOBO) and then reverse transcribed to synthesize cDNA using the ReverTra Ace qPCR RT Master Mix. Then, each cDNA template was amplified by quantitative polymerase chain reaction (qPCR) using the KAPA SYBR FAST qPCR Master Mix (2X) Kit (Kapa Biosystems) and gene-specific primers. The *TUBULIN* (*TUB*) gene was used as an internal reference. All reactions were repeated three times with three independent RNA samples.

### In vitro sumoylation assay

In vitro sumoylation assay was performed in 30 *μ*L of reaction buffer (50 mM Tris [pH 7.5], 5 mM MgCl_2_, 2 mM ATP); 500 ng of MBP-tagged proteins (SIZ1 and SSVs); 50 ng each of His_6_-AtSAE1b, His_6_-AtSAE2, and His_6_-AtSCE1; and 8 *μ*g each of His_6_-AtSUMO1-3 and His_6_-AtSUMO5, as previously reported (Park et al. 2011). After incubation for 1 h at 30 °C, the reaction mixtures were separated by sodium dodecyl sulfate–polyacrylamide gel electrophoresis (SDS–PAGE) on 8% (v/v) polyacrylamide gels. Sumoylated MBP-tagged proteins were detected by immunoblotting with anti-MBP antibody (Santa Cruz Biotechnology). To examine the effect of SSV2 on CNGC6 sumoylation, in vitro sumoylation assay was performed in 30 *μ*L of reaction buffer (same as above) with 500 ng of MBP-SSV2; 200 ng of GST-CNGC6; 50 ng each of His_6_-AtSAE1b, His_6_-AtSAE2, and His_6_-AtSCE1; and 8 *μ*g of His_6_-AtSUMO1. Sumoylated GST-CNGC6 was detected by immunoblotting with anti-GST antibody (sc-80998, Santa Cruz Biotechnology). In addition, to examine the self-sumoylation of HPY2 and its effect on CNGC6 sumoylation, in vitro sumoylation assays were performed using 500 ng of MBP-HPY2 under the reaction conditions described above. Sumoylated MBP-HPY2 and GST-CNGC6 were detected by immunoblotting with anti-MBP (sc-13564, Santa Cruz Biotechnology) and anti-GST antibodies.

### Subcellular localization analysis

*Agrobacterium tumefaciens* was transformed with various constructs (*35S-GFP*, *35S-GFP-SIZ1*, *35S-GFP-SSV1*, *35S-GFP-SSV2*, *35S-GFP-SSV3*, *35S-GFP-SIZ1-C1*, *35S-GFP-SIZ1-C2, 35S-GFP-SIZ1-N1, 35S-GFP-SIZ1-N2*, *35S-GFP-SSV2-N1*, *35S-GFP-SIZ1-CC1*, *35S-GFP-SIZ1-CC2*, *35S-GFP-CML35-NLS*, *35S-GFP-SSV2*, *35S-GFP-SIZ1-A1*, *35S-RFP-PIP2*, and *35S-RFP-SYP122*) and grown overnight at 28 °C in 50 mL of YEB liquid medium supplemented with appropriate antibiotics. Bacteria were harvested and resuspended in infiltration buffer containing 50 mM Tris (pH 7.4), 5% (w/v) sucrose, and 200 *μ*M acetosyringone. After incubation at 30 °C for 3 h, the transformed bacteria were infiltrated into the abaxial side of leaves of 6-wk-old *N. benthamiana* plants using a 1-mL needleless syringe without needle. Plants were then incubated in a growth chamber at 25 °C under 16 h light/8 h dark photoperiod for 2 d. GFP signal was detected in infiltrated leaves by confocal laser scanning microscopy (CLSM; Zeiss 710, excitation 480 nm argon laser, intensity: 50%, 505- to 530-nm band-pass filter, gain: 525).

### BiFC assay

To generate constructs for the BiFC assay, the *CNGC6* and *SSV2* cDNAs were cloned into the pDONR201 vector. Next, *SSV2* and *CNGC6* cDNAs were transferred from their respective entry clones to the gateway vector pSAT4-DEST-n(174)EYFP-C1 (ABRC stock number CD3-1089) or pSAT5-DEST-c(175-end)EYFP-C1(B) (ABRC stock number CD3-1097), which contained N-terminal 174 aa of EYFP (EYFP^N^) or C-terminal 64 aa of EYFP (YFP^C^), respectively. The fusion constructs encoding EYFP(N)-CNGC6 and EYFP(C)-SSV2 proteins were introduced into *A. tumefaciens*, and the transformed cells were coinjected into *N. benthamiana* leaves, as described above. YFP signal was detected in infiltrated leaves by CLSM (Excitation 514 nm argon laser, intensity: 50%, 520 to 550 nm band pass filter, gain: 500).

### Sumoylation assays

For in vivo sumoylation assays, *N. benthamiana* plants were infiltrated with different combinations of Agrobacterium strains transformed with *35S-Myc_6_-CNGC6*, *35S-GFP-SSV2*, and *35S-HA_3_-SUMO1-GG* constructs. After 3 d, total proteins were extracted from each sample and subjected to IP using an anti-HA antibody (ab184643, abcam) in a buffer containing 50 mM Tris (pH 8.0), 150 mM NaCl, 10% (v/v) glycerol, 1% (v/v) NP-40, 2 mM EDTA, 1 mM PMSF, and a protease inhibitor cocktail (Promega). Sumoylated CNGC6 was detected by western blotting using an anti-Myc antibody following IP.

### Qualitative and quantitative GUS assays

To generate the *XVE-SSV2* fusion, full-length *SSV2* cDNA was amplified by PCR using gene-specific primers and cloned into the pER8 plant expression vector. To generate the CNGC6-GUS fusion protein, a 3,425 bp fragment located upstream of the *CNGC6* start codon was amplified with gene-specific primers and cloned into the pEarleyGate 301 vector. Subsequently, the *CNGC6 cDNA-GUS* fusion was cloned into the recombinant plasmid, resulting in the *pCNGC6-CNGC6 cDNA-GUS* construct. Then, *XVE-SSV2* and *pCNGC6-CNGC6 cDNA-GUS* constructs were cointroduced into WT Arabidopsis by the floral dipping method (Clough and Bent 1998).

The effect of SSV2 on CNGC6 stability was examined using transgenic plants coexpressing *pCNGC-CNGC6 cDNA-GUS* and *XVE-SSV2*. Ten-day-old seedlings of two independent transgenic lines grown on MS media were incubated in β-estradiol-containing liquid medium for 15 h. Then, histochemical GUS enzyme activity was determined using two methods: staining with X-glucuronide (X-Glu, Merck) and fluorometric detection using 4-methylumbelliferyl-β-D-glucuronide (MUG, Merck) as the substrate, following the protocol outlined by Jefferson et al. (1987). In brief, for the GUS staining assay, transgenic seedlings were vacuum-infiltrated with staining buffer (50 mM Na H_2_PO_4_, pH 7.0; 0.1% (v/v) Triton X-100; 2 mM C_6_FeK_4_N_6_; 2 mM C_6_N_6_FeK_3_; and 2 mM X-Glu) for 10 min. After a 1-h incubation at 37 °C, the samples were destained by triple washing with 70% ethanol. For the quantitative GUS activity assay, the roots of transgenic seedlings were homogenized in GUS extraction buffer (50 mM NaH_2_PO_4_, pH 7.0; 10 mM EDTA; 0.1% (v/v) Triton-X100; 10 mM β-mercaptoethanol). Following quantification of the protein content in the supernatant, a portion of the supernatant was mixed with the reaction buffer containing 1 mM MUG. GUS activity was measured by monitoring the cleavage of the MUG substrate. Fluorescence was recorded using an LS50B PerkinElmer luminescence spectrometer. All measurements were conducted in triplicate.

To confirm *SSV2* and *CNGC6* expression, transgenic seedlings coexpressing *pCNGC-CNGC6 cDNA-GUS* and *XVE-SSV2* were treated with β-estradiol (as described above). Then, total RNA was isolated from these seedlings, and *CNGC6 cDNA-GUS* expression was investigated by RT-qPCR using *CNGC6*-specific primers. All reactions were repeated three times with three independent RNA samples. Additionally, total proteins were extracted from the same samples, and the level of SIZ1 protein was evaluated by immunoblot analysis with anti-AtSIZ1 antibody (The laboratory of Dr. Nam-Hai Chua, Rockefeller University). The experiments were repeated three times.

### Generation of transgenic *siz1-2* plants

To generate transgenic *siz1-2* mutants, the *pSIZ1-Myc_6_-SIZ1*, *pSIZ1-Myc_6_-SSV2*, *35S-Myc_6_-SIZ1*, and *35S-Myc_6_-SSV2* constructs were first generated. The *pSIZ1-Myc_6_-SIZ1* and *pSIZ1-Myc_6_-SSV2* constructs were generated by amplifying a 3,535 bp fragment upstream of the start codon of Arabidopsis *SIZ1* using gene-specific primers, fusing the PCR product to full-length *SIZ1* or *SSV2* cDNA, and then introducing each fusion into the pEarleygate 303 vector. The *35S-Myc_6_-SIZ1*, *35S-Myc_6_-SSV2*, and *35S-HA_3_-SSV2* constructs were generated by amplifying full-length *SIZ1* and *SSV2* cDNAs through PCR using *Myc_6_*- or *HA_3_*-tagged forward primers along with gene-specific reverse primers. The amplified cDNAs were then cloned into the plant expression vector pBA002. The resulting constructs, *35S-Myc_6_-SIZ1*, *35S-Myc_6_-SSV2*, *35S-HA_3_-SSV2*, *pSIZ1-Myc_6_-SIZ1*, and *pSIZ1-Myc_6_-SSV2*, were then separately introduced into *siz1-2* mutants by floral dipping.

To confirm the expression of *Myc_6_-SIZ1* and *Myc_6_-SSV2* in *pSIZ1-Myc_6_-SIZ1/siz1-2*, and *pSIZ1-Myc_6_-SSV2/siz1-2* plants, respectively, total RNA was extracted from the leaves of WT, *siz1-2*, *pSIZ1-Myc_6_-SIZ1/siz1-2*, and *pSIZ1-Myc_6_-SSV2/siz1-2* plants, and the levels of *Myc_6_-SIZ1* and *Myc_6_-SSV2* transcripts were estimated by reverse transcription-polymerase chain reaction (RT-PCR) using *Myc*- and *SIZ1*-specific primers. To confirm the expression of Myc_6_-SIZ1, Myc_6_-SSV2, and HA_3_-SSV2 proteins in *35S-Myc_6_-SIZ1/siz1-2*, *35S-Myc_6_-SSV2/siz1-2* and *35S-HA_3_-SSV2/siz1-2* plants, respectively, total protein was extracted from the leaves of WT, *siz1-2*, *35S-Myc_6_-SIZ1/siz1-2*, *35S-Myc_6_-SSV2/siz1-2*, and *35S-HA_3_-SSV2/siz1-2* plants. The levels of Myc_6_-SIZ1, Myc_6_-SSV2, and HA_3_-SSV2 proteins were evaluated by immunoblot analysis using anti-Myc (ab32072, Abcam) or anti-HA antibodies.

### Subcellular localization analysis of SIZ1 and SSV2 in transgenic *siz1-2* plants

To determine the subcellular localization of SIZ1 and SSV2 proteins, the *pSIZ1-GFP-SIZ1* and *pSIZ1-GFP-SSV2* constructs were generated. Briefly, a 3,535 bp promoter fragment of *SIZ1* was amplified by PCR using gene-specific primers (as described above) and fused to the *GFP-SIZ1* or *GFP-SSV2* cDNA. The resulting fusions, *pSIZ1-GFP-SIZ1* and *pSIZ1-GFP-SSV2*, were separately cloned into the pEarleygate 303 vector, and the final recombinant plasmids were separately transformed into *siz1-2* mutants via the floral dipping method. To confirm *GFP-SIZ1* and *GFP-SSV2* expression in *pSIZ1-GFP-SIZ1/siz1-2* and *pSIZ1-GFP-SSV2/siz1-2* plants, respectively, total RNA was extracted from the leaves of WT, *pSIZ1-GFP-SIZ1/siz1-2*, and *pSIZ1-GFP-SSV2/siz1-2* plants, and the levels of *GFP-SIZ1* and *GFP-SSV2* transcripts were estimated by RT-PCR using *GFP*- and *SIZ1*-specific primers. GFP signals in the roots of *pSIZ1-GFP-SIZ1/siz1-2* and *pSIZ1-GFP-SSV2/siz1-2* plants were detected by CLSM (excitation 514 nm argon laser, intensity: 50%, 520 to 550 nm band pass filter, gain: 500).

### Separation of nuclear, cytosolic, and plasma membrane fractions

Three-week-old light-grown *siz1-2* plants (16 h light/8 h dark) carrying the *35S-HA_3_-SSV2* transgene on MS medium were harvested and ground in liquid nitrogen using a mortar and pestle in the presence of 10 mL grinding buffer (0.3 M sucrose, 40 mM Tris (pH 7.5), 5 mM MgCl_2_, 1 mM PMSF, and a protease inhibitor cocktail). The homogenate was filtered through four layers of cheesecloth and then centrifuged at 1,500 × *g* for 10 min at 4 °C to pellet the nuclei. The supernatant (S1 fraction) was carefully separated from the pellet and transferred to new tubes. This S1 fraction was subjected to centrifugation at 12,000 × *g* for 20 min at 4 °C, and the resulting supernatant underwent further centrifugation at 100,000 × *g* for 1 h at 4 °C to yield the soluble cytosol fraction in the resultant supernatant. The pellet, which represented the membrane fraction, was subsequently resuspended in 0.1 mL of grinding buffer.

To detect the HA_3_-SSV2 protein, total proteins were extracted from the nuclear, cytosolic, and plasma membrane fractions and subsequently analyzed by western blotting using the anti-HA antibody. Similarly, histone 3, tubulin, and H^+^-ATPase were identified through immunoblot analysis using the anti-histone 3 (ab1791, Abcam), anti-tubulin (sc-33999, Santa Cruz Biotechnology), and anti-H^+^-ATPase (AS07260, Agrisera) antibodies, respectively.

### Effects of SSV2 overexpression on CNGC6 concentration in vivo

Fourteen-day-old light-grown plants (16 h light/8 h dark) carrying *35S-Myc_6_-CNGC6* and *XVE-HA_3_-SSV2* or *35S-Myc_6_-CNGC6* and *XVE-HA_3_-mSSV2* transgenes on MS medium were subjected to different treatments. mSSV2 represents a variant of SSV2 in which specific residues (Cysteine 379 and Tryptophan 400) within the RING motif have been mutated to serine and alanine (C379S, W400A). This variant was generated through site-directed mutagenesis using PCR with specific mutated primers. The plants were treated with or without β-estradiol in the presence of light for 15 h. Afterward, the samples were ground in liquid nitrogen, and the lysates were separated by SDS–PAGE. The levels of Myc_6_-CNGC6 were determined by immunoblot analysis using an anti-Myc antibody. The induction of SSV2 or mSSV2 was analyzed by western blotting using an anti-HA antibody. Additionally, the post-translational degradation of CNGC6 was examined using double transgenic plants carrying *35S-Myc_6_-CNGC6* and *XVE-HA_3_-SSV2* or *35S-Myc_6_-CNGC6* and *XVE-HA_3_-SSV2* constructs. These transgenic plants were treated with β-estradiol to induce SSV2 or mSSV2 expression, followed by washing and transfer to MS medium supplemented with 100 *µ*M CHX. The treated plants were incubated for 4 h, and protein samples were extracted at specific time points for analysis by immunoblot analysis using an anti-Myc antibody.

### Detection of SUMO-conjugates in SSV2-expressing transgenic *siz1-2* plants

To investigate the effect of SSV2 overexpression on the sumoylation of target proteins, the seeds of WT and transgenic *siz1-2* mutants (*35S-Myc_6_-SIZ1/siz1-2* and *35S-Myc_6_-SSV2/siz1-2*) were sown in soil. The plants were grown in a growth chamber under 16 h light/8 h dark cycle at 22 °C. After 3 wk of growth, the plants were subjected to heat stress (39 °C) for 15 min. Total proteins were extracted from the samples, and the levels of SUMO-conjugates were examined through immunoblot analysis using an anti-AtSUMO1 antibody (ab5316, abcam).

### Sumoylation assay in *E. coli*

*E. coli* BL21(DE3) cells were transformed with *pACYCDuet-AtSAE1b-AtSAE2* plasmid to prepare competent cells (Okada et al. 2009). For the in vivo sumoylation reaction, the competent cells were then transformed with two plasmids: *pGEX2T-CNGC6-HA* (or K to R mutants) and *pCDFDuet-AtSUMO1-AtSCE1a*. The transformed cells were cultured in LB medium at 37 °C until reaching an OD6_00nm_ of 0.6. Then, they were induced with 0.5 mM isopropyl β-D-1-thiogalactopyranoside (IPTG) and further incubated for 12 h at 25 °C. After that, the cells were harvested by centrifugation, and the resulting pellet was resuspended in lysis buffer containing 50 mM Tris–HCl (pH 7.5), 150 mM NaCl, 0.1% (v/v) Triton X-100, 10 mM EDTA, and 1 mM PMSF. The suspension was sonicated, followed by centrifugation. The resulting supernatants were separated by 8% SDS–PAGE, and the sumoylation of GST-CNGC6-HA was detected using an anti-HA antibody (sc-7392, Santa Cruz Biotechnology).

### Heat tolerance analysis of transgenic *siz1-2* and *cngc6* plants

To conduct heat tolerance assays, the seeds of WT, *siz1-2*, and transgenic *siz1-2* mutants (*35S-Myc_6_-SIZ1/siz1-2*, *35S-Myc_6_-SSV2/siz1-2*, *pSIZ1-Myc_6_-SIZ1/siz1-2*, and *pSIZ1-Myc_6_-SSV2/siz1-2*) were sown in soil, and the plants were grown under 16 h light/8 h dark cycle at 22 °C in a growth chamber. After 3 wk, all plants were subjected to a heat stress treatment (37 °C for 5 d; 22 °C for 2 d; 45 °C for 8 h; 22 °C for 7 d) and photographed.

To assess the role of sumoylation on the function of CNGC6 regarding heat tolerance, we constructed recombinant plasmids: *35S-CNGC6-Myc_6_*, *35S-mCNGC6(K66R)-Myc_6_*, *35S-mCNGC6(K347R)-Myc_6_*, *35S-mCNGC6(K578R)-Myc_6_*, *35S-mCNGC6(K669)-Myc_6_*, and *35S-mCNGC6(675R)-Myc_6_*. These plasmids were then introduced into *cngc6* mutants through floral dipping. After selecting homozygous lines, the expression of CNGC6-Myc_6_, mCNGC6(K66R)-Myc_6_, mCNGC6(K347R)-Myc_6_, mCNGC6(K578R)-Myc_6_, mCNGC6(K669R)-Myc_6_, and mCNGC6(K675R)-Myc_6_ proteins was examined by western blotting using an anti-Myc antibody. To conduct heat tolerance assays, we sowed seeds of WT, *cngc6*, and transgenic *cngc6* mutants (*35S-CNGC6-Myc_6_/cngc6*, *35S-mCNGC6(K66R)-Myc_6_/cngc6*, *35S-mCNGC6(K347R)-Myc_6_/cngc6*, *35S-mCNGC6(K578R)-Myc_6_/cngc6*, *35S-mCNGC6(K669)-Myc_6_/cngc6*, and *35S-mCNGC6(675R)-Myc_6_/cngc6*) were sown in soil. These plants were then grown in a growth chamber under 16 h light/8 h dark cycle at 22 °C. After 3 wk, all plants were subjected to a heat stress treatment as described above.

### Source of the databases for splicing variants

We obtained data on the splicing variants of Arabidopsis *SIZ1* orthologues from seven currently available databases in plant, animal, and yeast systems. The databases used were as follows: Rice (*O. sativa*) Genome Annotation Project (RGAP) (Reference: Nipponbare) for rice, maize (*Z. mays*) genome database (ZmGBD) (Reference: B73) for Maize, soybean (*G. max*) genome database (GmGBD) (Reference: Williams 82) for soybean, National Center for Biotechnology Information (NCBI) (Reference: Heinz 1706) for tomato, University of California Santa Cruz (UCSC) Genome Browser (Reference: GRCh38/hg38) for human, University of California Santa Cruz (UCSC) Genome Browser (Reference: GRCm38/mm38) for mouse, and Saccharomyces Genome Database (SGD) (Reference: S288C) for yeast.

### Accession numbers

Sequence data from this article can be found in the GenBank/EMBL data libraries under accession numbers: SIZ1 (At5g60410), SUMO1 (At4g26840), SUMO2 (At5g55160), SUMO3 (At5g55170), SUMO5 (At2g32765), and CNGC6 (At2g23980).

## Supplementary Material

kiae108_Supplementary_Data

## Data Availability

All data are included in the manuscript and supplemental files.

## Supplementary data

The following materials are available in the online version of this article.

**Supplementary Figure S1.** Effect of the NLS and SSV2-specific peptides on the subcellular localization of SIZ1.

**Supplementary Figure S2.** Subcellular distribution analysis of SSV2.

**Supplementary Figure S3.** Analysis of the direct physical interaction of SIZ1 and SSVs with SUMO proteins and SUMO-conjugating enzyme E2 by Y2H assays.

**Supplementary Figure S4.** Genomic DNA sequence of Arabidopsis *SIZ1* gene.

**Supplementary Figure S5.** Examination of the effects of abiotic stresses, hormones, and nitrogen sources on the expression of *SIZ1* and *SSVs*.

**Supplementary Figure S6.** Nucleotide sequences of the 3′ regions of *SSV2* and *SSV5*.

**Supplementary Figure S7.** Nucleotide sequences of the 3′ end of *SIZ1*, *SSV2*, and *SSV5*, and their deduced amino acid sequences.

**Supplementary Figure S8.** CNGC6 is stabilized by SSV2 in vivo.

**Supplementary Figure S9.** Transgenic *siz1-2* mutant plants overexpressing *SSV2* under the control of the *35S* promoter are tolerant to heat stress.

**Supplementary Figure S10.** Analysis of the heat tress tolerance of *pSIZ1-GFP-SSV2*/*siz1-2* plants.

**Supplementary Figure S11.** SUMO-conjugates were examined in SSV2-expressing transgenic *siz1-2* plants under heat stress.

**Supplementary Figure S12.** Identification of the sumoylation site on CNGC6.

**Supplementary Figure S13.** Splicing variants of putative *Arabidopsis thaliana* SIZ1 orthologs in rice, corn, soybean, tomato, human, and mouse and yeast.

**Supplementary Table S1.** List of primers used for this study.

## Dive Curated Terms

The following phenotypic, genotypic, and functional terms are of significance to the work described in this paper:

SCE1 Gramene: AT3G57870
SCE1 Araport: AT3G57870

SLY1 Gramene: AT4G24210
SLY1 Araport: AT4G24210

MYB30 Gramene: At3g28910
MYB30 Araport: At3g28910

NIA1 Gramene: AT1G77760
NIA1 Araport: AT1G77760

NIA2 Gramene: AT1G37130
NIA2 Araport: AT1G37130

SYP122 Gramene: AT3G52400
SYP122 Araport: AT3G52400

ABI5 Gramene: At2g36270
ABI5 Araport: At2g36270

CMT3 Gramene: AT1G69770
CMT3 Araport: AT1G69770

FLD Gramene: AT3G10390
FLD Araport: AT3G10390

SIZ1 Gramene: AT5G60410
SIZ1 Araport: AT5G60410

FLC Gramene: AT5G10140
FLC Araport: AT5G10140

PHR1 Gramene: AT4G28610
PHR1 Araport: AT4G28610

COP1 Gramene: AT2G32950
COP1 Araport: AT2G32950

ICE1 Gramene: Solyc03g118310
ICE1 Araport: Solyc03g118310
