# Supplementary material for: E3 SUMO ligase SIZ1 splicing variants localize and function according to external conditions
Source: Plant Physiol. 2024 Mar 18;195(2):1601–23. doi: 10.1093/plphys/kiae108 (PMC11142376; doi:10.1093/plphys/kiae108)
Supplement: kiae108_Supplementary_Data [file kiae108_supplementary_data.zip › kiae108_Supplementary_Data.pdf]

Supplemental Data

Title: E3 SUMO ligase SIZ1 splicing variants localize and function according to external conditions

Short title: Production and function of SIZ1 Splicing Variant 2

Jun Soo Kwak[1], Jong Tae Song[2], Hak Soo Seo[1,3,*]

[1]Department of Agriculture, Forestry and Bioresources, Research Institute of Agriculture and Life Sciences, Seoul National University, Seoul 08826, Korea

[2]Department of Applied Biosciences, Kyungpook National University, Daegu 41566, Korea

[3]Bio-MAX Institute, Seoul National University, Seoul 08826, Korea

[*]**Author for contact:**
Hak Soo Seo
Tel.: +82-2-880-4548
Fax: +82-2-873-2056
E-mail: seohs@snu.ac.kr

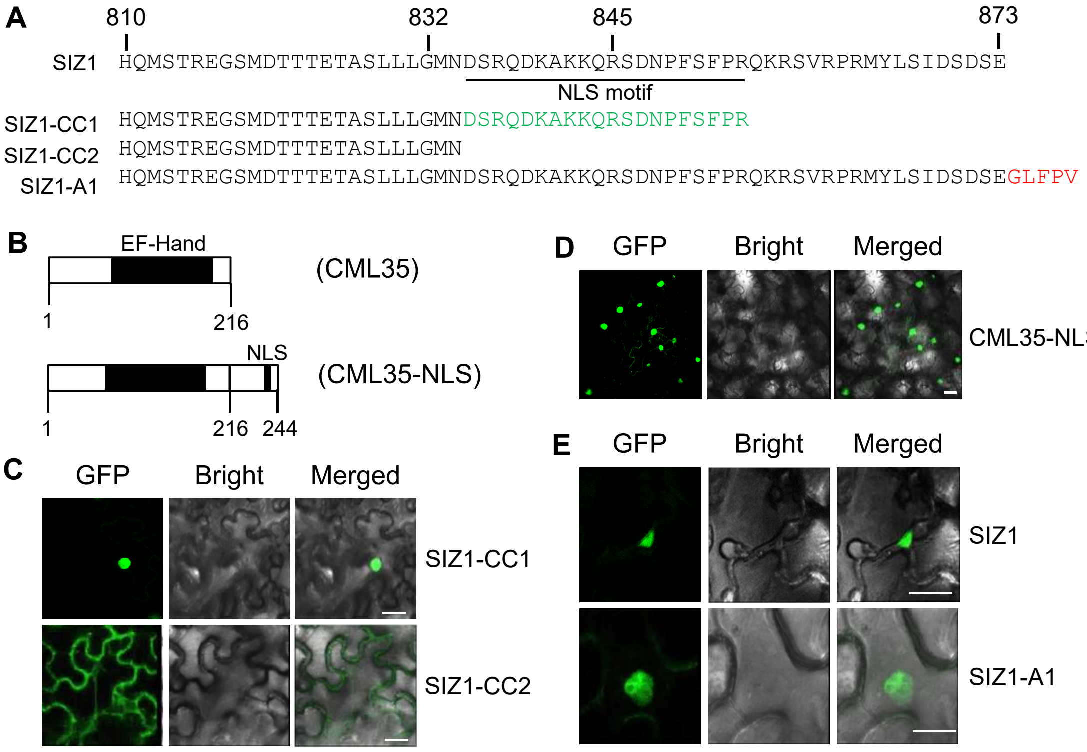

Supplemental Figure S1. Effect of the nuclear localization signal (NLS) and SSV2-specific peptides on the subcellular localization of SIZ1. (**A**) Amino acid sequences of full-length SIZ1 protein, SIZ1 C-terminal deletion mutant proteins, and recombinant SIZ1 protein. The NLS motif is indicated in green, and the amino acid sequence of the SSV2-specific peptide is indicated in red. (**B**) Schematic representation of the CML35-NLS recombinant protein. (**C**) Subcellular location analysis of the C-terminal deletion mutant proteins of SIZ1. The *35S-GFP-SIZ1-CC1* and *35S-GFP-SIZ1-CC2* constructs were individually introduced into *N. benthamiana* leaves by agroinfiltration. After incubation at 25°C for 2 d, the GFP signal was detected by CLSM. Scale bar, 20 μm. (**D**) Effect of the NLS motif on the subcellular localization of CML35. The *35S-GFP-CML35-NLS* construct was introduced into *N. benthamiana* leaves by agroinfiltration, and GFP signal was analyzed by CLSM. Scale bar, 20 μm. (**E**) Subcellular location analysis. The *35S-GFP-SIZ1* and *35S-GFP-SIZ1-A1* constructs were individually introduced into *N. benthamiana* leaves by agroinfiltration. After incubation at 25°C for 2 d, the GFP signal was detected by CLSM. Scale bar, 10 μm.

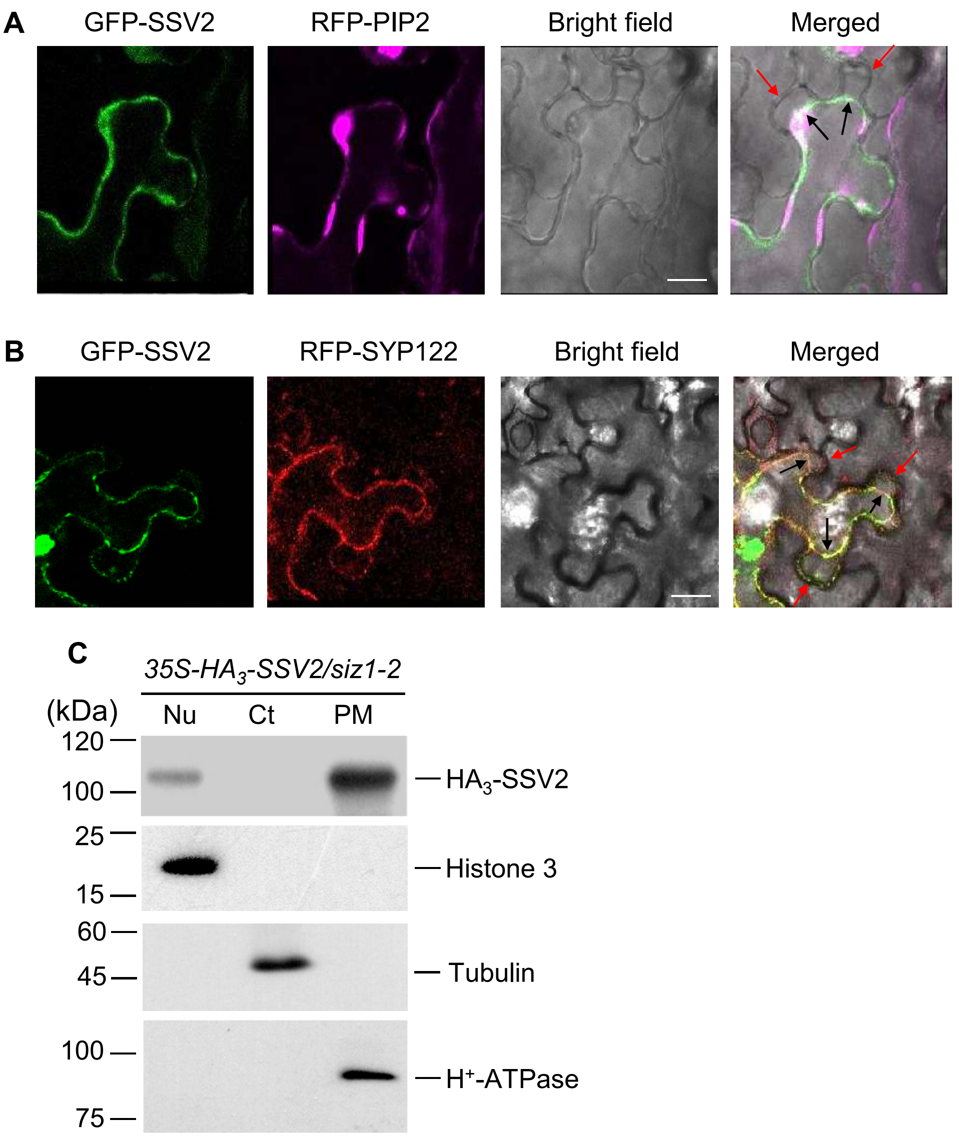

Supplemental Figure S2. Subcellular distribution analysis of SSV2. *N. benthamiana* leaves were coinfiltrated either with *35S-GFP-SSV2* and *35S-RFP-PIP2* (**A**) or with *35S-GFP-SSV2* and *35S-RFP-SYP122* (**B**). The agroinfiltrated leaves were incubated at 25°C for 2 d, and then floated on 1 M NaCl for 30 min. Subsequently, GFP and RFP signals were detected by CLSM. Red arrows indicate the cell wall, and black arrows indicate the cytoplasmic membrane. Scale bar, 10 µm. (**C**) Nuclear, cytosolic, and plasma membrane fractions were isolated from transgenic *siz1-2* plants expressing *35S-HA$_3$-SSV2* and then HA$_3$-SSV2 was examined by western blot analysis with an anti-HA antibody. Histone 3, tubulin, and H^+-ATPase were used as nucleus-, cytosol-, and plasma membrane-specific loading controls and detected by anti-histone 3, anti-tubulin, and anti-H^+-ATPase antibodies, respectively. Nu, nucleus; Ct, cytosol; PM, plasma membrane.

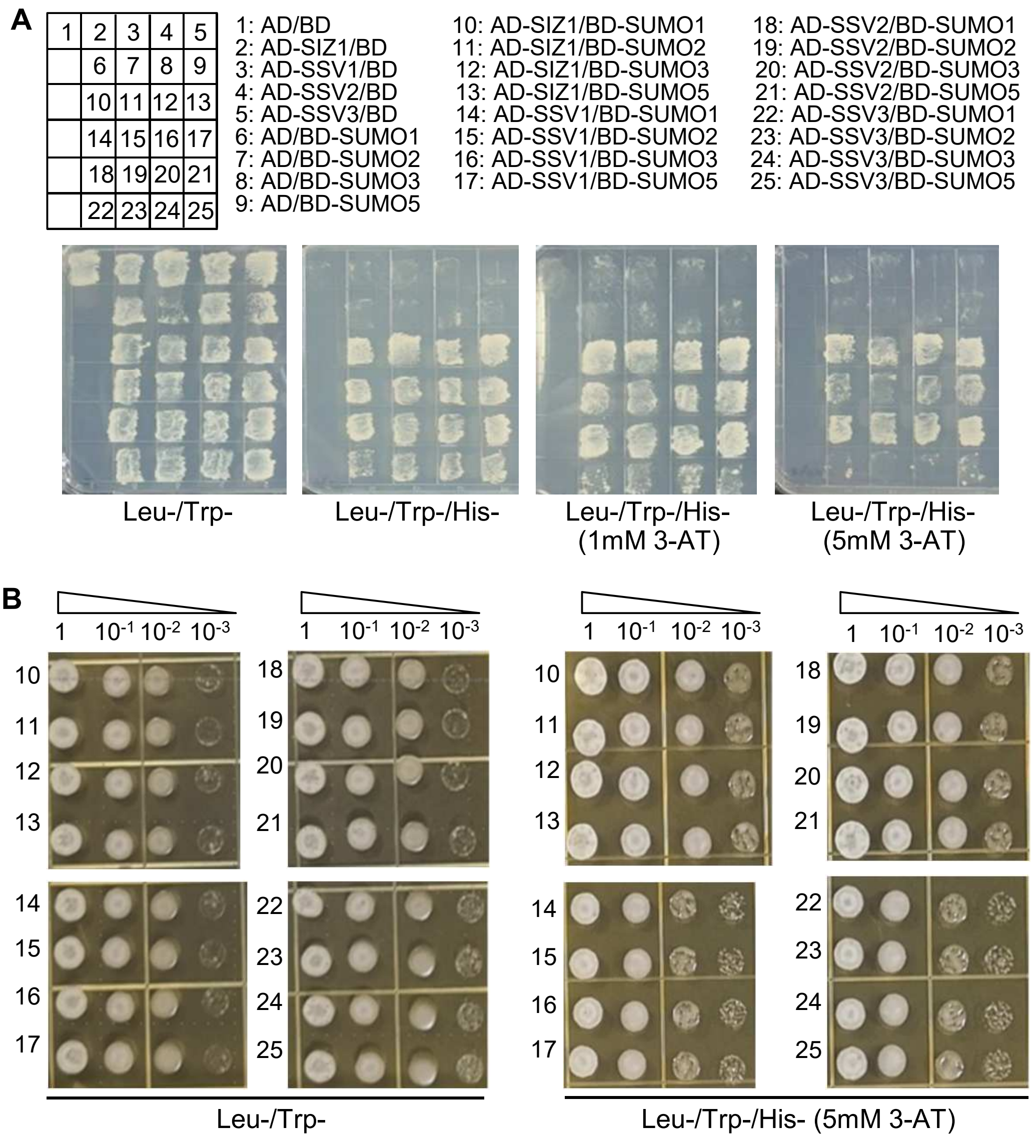

Supplemental Figure S3. Continued

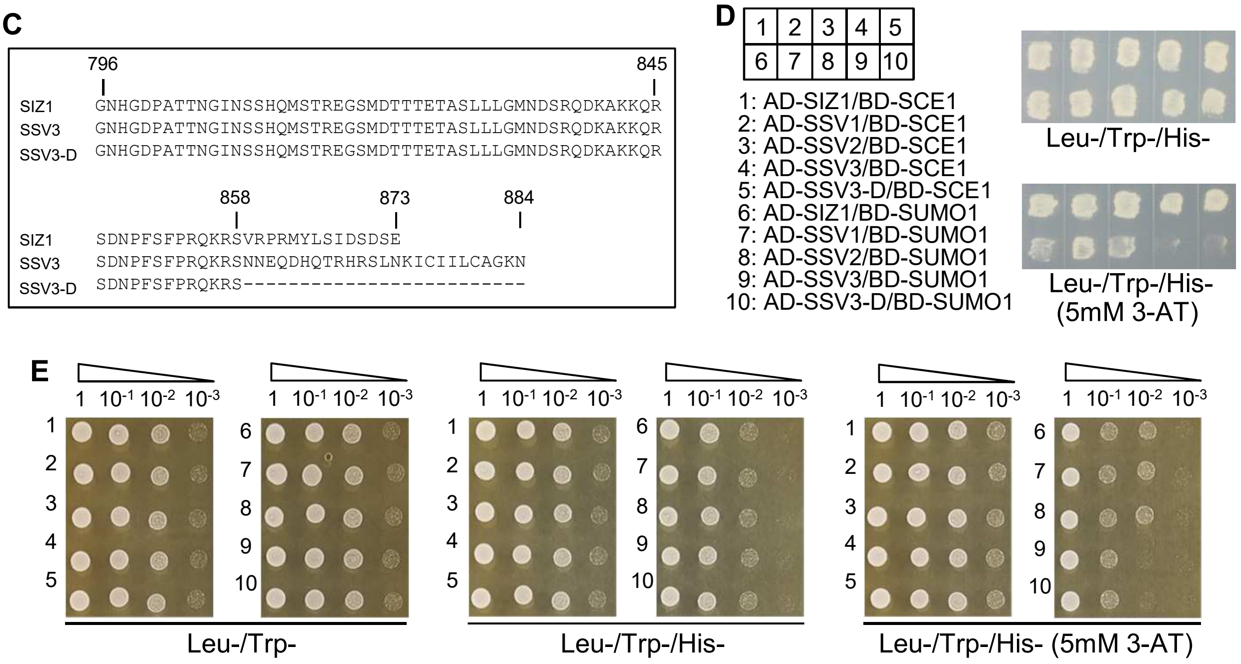

Supplemental Figure S3. Analysis of the direct physical interaction of SIZ1 and SSVs with SUMO proteins and SUMO-conjugating enzyme E2 by yeast two-hybrid (Y2H) assays. The cDNAs of *SIZ1*, *SSV1*, *SSV2*, *SSV3*, and *SSV3-D* (a deletion mutant of *SSV3*) were fused to the sequence encoding the GAL4 activation domain (AD) in pGAD424. Arabidopsis *SUMO1*, *SUMO2*, *SUMO3*, *SUMO5*, and *SCE1* cDNAs were fused to the sequence encoding the GAL4 DNA-binding domain (BD) in pGBT8. The AD and BD constructs were cotransformed into the yeast strain AH109. (**A**) Analysis of the interaction of SIZ1 and SSVs with SUMO proteins. Each number indicates a specific combination of pGAD424 and pGBT8 vectors introduced into yeast cells. The combinations of vectors used for yeast transformation are also described in the box. Transformants were plated on different types of minimal media (-Leu/-Trp, -Leu/-Trp/-His, -Leu/-Trp/-His/+1 mM 3-amino-1,2,4-triazole (3-AT), and -Leu/-Trp/-His/+5 mM 3-AT), incubated for 4 d, and then photographed. (**B**) Serial dilutions of the corresponding yeast cells spotted on two different types of minimal media (-Leu/-Trp and -Leu/-Trp/-His/+5 mM 3-AT). Photographs were taken after cultivation for 4 d. (**C**) Amino acid sequences of full-length SSV3 and its deletion mutant SSV3-D. (**D**) Analysis of the interaction of SIZ1 and SSVs with the E2 enzyme SCE1, and that of SSV3-D with SUMO1. Each number indicates a specific combination of pGAD424 and pGBT8 vectors introduced into yeast cells. The combinations of vectors used for yeast transformation are also described in the box. Transformants were plated onto different minimal media (-Leu/-Trp and -Leu/-Trp/-His/+5 mM 3-AT), incubated for 4 d, and then photographed. (**E**) Serial dilutions of the corresponding yeast cells spotted on three different types of minimal media (-Leu/-Trp, -Leu/-Trp/-His and -Leu/-Trp/-His/+5 mM 3-AT). Photographs were taken after cultivation for 4 d.

A

TCTGTTCCTGTGGTTCCTGGCTCTACTGGCCGATCTGAAGCAAACGATGGCCTAGTTGACAATCCTCTTGCATTTGGTAG
AGACGATCCCTC**ACTTCAAATATTTTTGCCAACA**AAACCAGATGCTTCAGCTCAGTCGGGTTTTAAAAACCAAGCTGATAT
2500F
GTCAAATGGTCTCCGTAGTGAAGACTGGATCTCGCTTAGGCTAGGCGATAGCGCCTCTGGGAATCATGGAGATCCTGCAA
CTACAAACGGGATTAACTCAAGCCATCAGATGTCTACGAGGGAAGGTTCTA**TGGATACTACAACAGAGAC**TGgtttgttccctg
2700F
Tctgagactaatgctttcctgaaaacatcaaagggacctaaaaatgtggaccatgttcttgatgttgcattaccttttcatcactttactaagctgctagctaggtcttataaacaactga
agaagaaatatctgttttagtttctatgtggcgtattttgtctttgaatatgctactttatatatgtccatagtccatgtggtatacttactgactattactgcgtatttgcagCGTCGTTG
CTTCTGGGTATGAAT**GACAGTAGACAAGACAAGGCAA**AGAAGCAAAGATCAGATAATCATTTTCATTTCCTCGCCAGAA
3013R
GCGTTCTGTAAGACCTCGGATGTACCTCTCCATTGACTCGGATTCTGAGTAAtattcttcctgatttgtctgttctgttgtgtctctcttgggatgattcg
gtgttccttttgattcttaacgctatgcacagaacaatgaacaggatcatcagacaagacaccggagtttaaacaagatttgcataattctctgtgcaggcaagaattgaaccggtatt
gatattttcacttgtatgatgttgttgactctctcttcaatatcggttcagaatcttggccttgtctgctacactgcaggatgtaatttgcaaagcgaagcactggctgatttagtttctctgata
gaaaagaaaagtgggcacagtggttccgatttaattagtagtttgtatactcgaaataggttttttttgtgtggacgatgataaaattacttcgaagccaggag**ctatagagagatata**
gcaatgtaaattatgggctccaaattttatttattaattagaaaaattcagctcttctcctccattgcaaatt
3280R

B

```
2500F: 5′-ACTTCAAATATTTTTGCCAACA-3′
2700F: 5′-TGGATACTACAACAGAGACT-3′
3013R: 5′-TTGCCTTGTCTTGTCTACTGTC-3′
3280R: 5′-ACATTGCTATATCTCTCTATAG-3′
```

Supplemental Figure S4. Genomic DNA sequence of Arabidopsis *SIZ1* gene. (**A**) Nucleotide sequence of the 3'-end of the *SIZ1* gene. The underlined nucleotide sequences in bold font indicate the positions of forward primers (2500F and 2700F) and reverse primers (3013R and 3280R). Exons and introns are indicated with uppercase and lowercase letters, respectively. (**B**) Nucleotide sequences of 2500F, 2700F, 3013R, and 3280R primers.

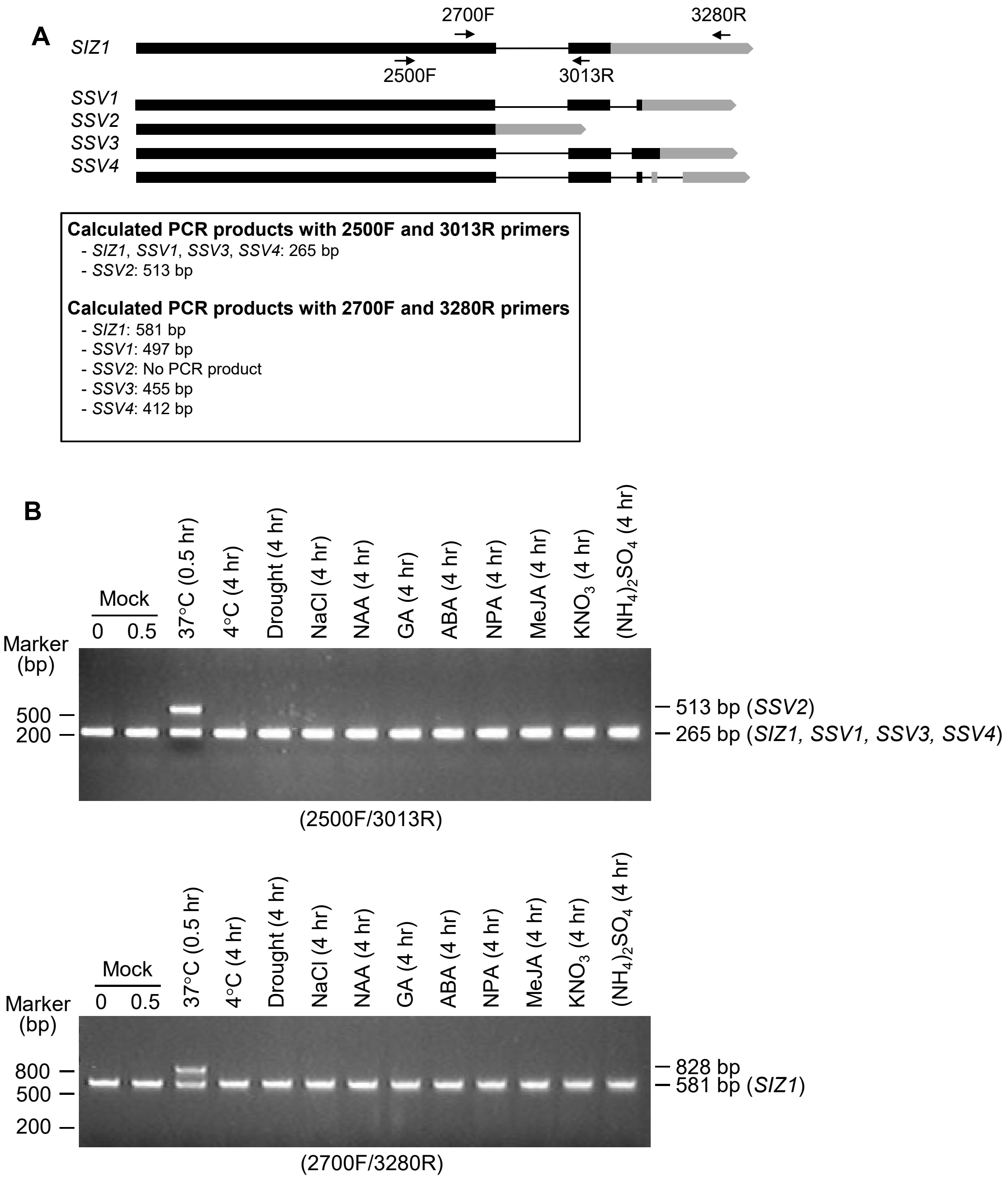

Supplemental Figure S5. Examination of the effects of abiotic stresses, hormones, and nitrogen sources on the expression of *SIZ1* and *SSVs*. (**A**) Structures of the 3' regions of *SIZ1*, *SSV1*, *SSV2*, *SSV3*, and *SSV4*. Arrows indicate the positions of forward primers (2500F and 2700F) and reverse primers (3013R and 3280R). The sizes of PCR products are shown below. (**B**) Expression analysis of *SIZ1* and *SSVs* under different conditions. Total RNAs were isolated from Arabidopsis plants treated with various abiotic stresses, hormones, and nitrogen sources for the indicated time points. RT-PCR was performed with two primer sets, 2500F/3013R (upper) or 2700F/3280R (lower), and PCR products were analyzed by agarose gel electrophoresis. Asterisk means a new splicing variant of SIZ1.

SSV2

ttcctggctc tactggccga tctgaagcaa acgatggcct agttgacaat cctcttgcat ttggtagaga cgatccctca cttcaaatat ttttgccaac
aaaaccagat gcttcagctc agtcgggttt taaaaaccaa gctgatatgt caaatggtct ccgtagtgaa gactggatct cgcttaggct aggcgatagc
gcctctggga atcatggaga tcctgcaact acaaacggga ttaactcaag ccatcagatg tctacgaggg aaggttctat ggatactaca acagagactg
gtttgttccc tgtc**tga**act aatgctttcc tgaaaacatc aaagggacct aaaaatgtgg accatgttct tgatgttgca ttacctttt catcacttta
ctaagctgct agctaggtct tataaacaac tgaagaagaa atatctgttt tagtttctat gtggcgtatt ttgtctttga atatgctact ttatatatgt
ccatagtcca tgtggtatac ttactgacta ttactgcgta tttgcagcgt cgttgcttct gggtatgaat gacagtagac aagacaaggc aa

PCR product (2500F/3013R): 513 bp, PCR product (2700F and 3280R): No product

SSV5

ttcctggctc tactggccga tctgaagcaa acgatggcct agttgacaat cctcttgcat ttggtagaga cgatccctca cttcaaatat ttttgccaac
aaaaccagat gcttcagctc agtcgggttt taaaaaccaa gctgatatgt caaatggtct ccgtagtgaa gactggatct cgcttaggct aggcgatagc
gcctctggga atcatggaga tcctgcaact acaaacggga ttaactcaag ccatcagatg tctacgaggg aaggttctat ggatactaca acagagactg
gtttgttccc tgtc**tga**act aatgctttcc tgaaaacatc aaagggacct aaaaatgtgg accatgttct tgatgttgca ttacctttt catcacttta
ctaagctgct agctaggtct tataaacaac tgaagaagaa atatctgttt tagtttctat gtggcgtatt ttgtctttga atatgctact ttatatatgt
ccatagtcca tgtggtatac ttactgacta ttactgcgta tttgcagcgt cgttgcttct gggtatgaat gacagtagac aagacaaggc aaagaagcaa
agatcagata atcattttca tttcctcgcc agaagcgttc tgtaagacct cggatgtacc tctccattga ctcggattct gagtaatatt cttcctgatt
tgtctgttct gttgtgtctc tcttgggatg attcggtgtt ccttttgatt cttaacgctat gcacagaaca atgaacagga tcatcagaca agacaccgga
gtttaaacaa gatttgcata attctctgtg caggcaagaa ttgaaccggt attgatattt tcacttgtat gatgttgttg actctctctt caatatcggt
tcagaatctt ggccttgtct gctacactgc aggatgtaat ctgcaaagcg aagcactggc tgatttagtt tctctgatag aaaagaaaag tgggcacagt
ggttccgatt taattagtag tttgtatact cgaaataggt tttttttgtg tggacgatga taaaattact tcgaagccag gagctataga gagatatagc
aatgt

PCR product (2500F/3013R): 513 bp, PCR product (2700F and 3280R): 828 bp

Supplemental Figure S6. Nucleotide sequences of the 3' regions of *SSV2* and *SSV5*. Primer sequences used for RT-PCR are underlined, and translation termination codons are indicated in bold font. Blue font indicates the nucleotide sequence present in *SSV5* but absent in *SSV2*.

SIZ1

```
TG GAT ACT ACA ACA GAG ACT GCG TCG TTG CTT CTG GGT ATG AAT GAC AGT AGA CAA GAC AAG GCA AAG AAG CAA AGA TCA GAT
    D   T   T   T   E   T   A   S   L   L   L   G   M   N   D   S   R   Q   D   K   A   K   K   Q   R   S   D
AAT CCA TTT TCA TTT CCT CGC CAG AAG CGT TCT GTA AGA CCT CGG ATG TAC CTC TCC ATT GAC TCG GAT TCT GAG TAA
 N   P   F   S   F   P   R   Q   K   R   S   V   R   P   R   M   Y   L   S   I   D   S   D   S   E   *
tattcttcctgatttgtctgttctgttgtgtctctcttgggatgattcggtgttccttttgattcttaacgctatgcacagaacaatgaacaggatcatcagacaagacacc
ggagtttaaacaagatttgcataattctctgtgcaggcaagaattgaaccggtattgatattttcacttgtatgatgttgttgactctctcttcaatatcggttcagaatct
tggccttgtctgctacactgcaggatgtaatttgcaaagcgaagcactggctgatttagtttctctgatagaaaagaaaagtgggcacagtggttccgatttaattagtagt
ttgtatactcgaaataggttttttttgtgtggacgatgataaaattacttcgaagccaggagctatagagagatatagcaatgtaaattatgggctccaaattttatttatt
aattagaaaaattcagctcttctcctcc
```

SSV2

```
TG GAT ACT ACA ACA GAG ACT Ggt ttg ttc cct gtc tga actaatgctttcctgaaaacatcaaagggacctaaaaatgtggaccatgttcttgatgttgc
    D   T   T   T   E   T   G   L   F   P   V   *
attaccttttcatcactttactaagctgctagctaggtcttataaacaactgaagaagaaatatctgttttagtttctatgtggcgtattttgtctttgaatatgctacttt
atatatgtccatagtccatgtggtatacttactgactattactgcgtatttgcagCGTCGTTGCTTCTGGGTATGAATGACAGTAGACAAGACAAGGCAA
```

SSV5

```
TG GAT ACT ACA ACA GAG ACT Ggt ttg ttc cct gtc tga actaatgctttcctgaaaacatcaaagggacctaaaaatgtggaccatgttcttgatgttg
    D   T   T   T   E   T   G   L   F   P   V   *
cattaccttttcatcactttactaagctgctagctaggtcttataaacaactgaagaagaaatatctgttttagtttctatgtggcgtattttgtctttgaatatgctacttt
atatatgtccatagtccatgtggtatacttactgactattactgcgtatttgcagCGTCGTTGCTTCTGGGTATGAATGACAGTAGACAAGACAAGGCAAAGAAGCAAAGATCA
GATAATCATTTTCATTTCCTCGCCAGAAGCGTTCTGTAAGACCTCGGATGTACCTCTCCATTGACTCGGATTCTGAGTAAtattcttcctgatttgtctgttctgttgtgtctc
tcttgggatgattcggtgttccttttgattcttaacgctatgcacagaacaatgaacaggatcatcagacaagacaccggagtttaaacaagatttgcataattctctgtgcag
gcaagaattgaaccggtattgatattttcacttgtatgatgttgttgactctctcttcaatatcggttcagaatcttggccttgtctgctacactgcaggatgtaatctgcaaa
gcgaagcactggctgatttagtttctctgatagaaaagaaaagtgggcacagtggttccgatttaattagtagtttgtatactcgaaataggttttttttgtgtggacgatgat
aaaattacttcgaagccaggagctatagagagatatagcaatgt
```

Supplemental Figure S7. Nucleotide sequences of the 3' end of *SIZ1*, *SSV2*, and *SSV5*, and their deduced amino acid sequences. SSV2- and SSV5-specific amino acid sequences are indicated in bold red font.

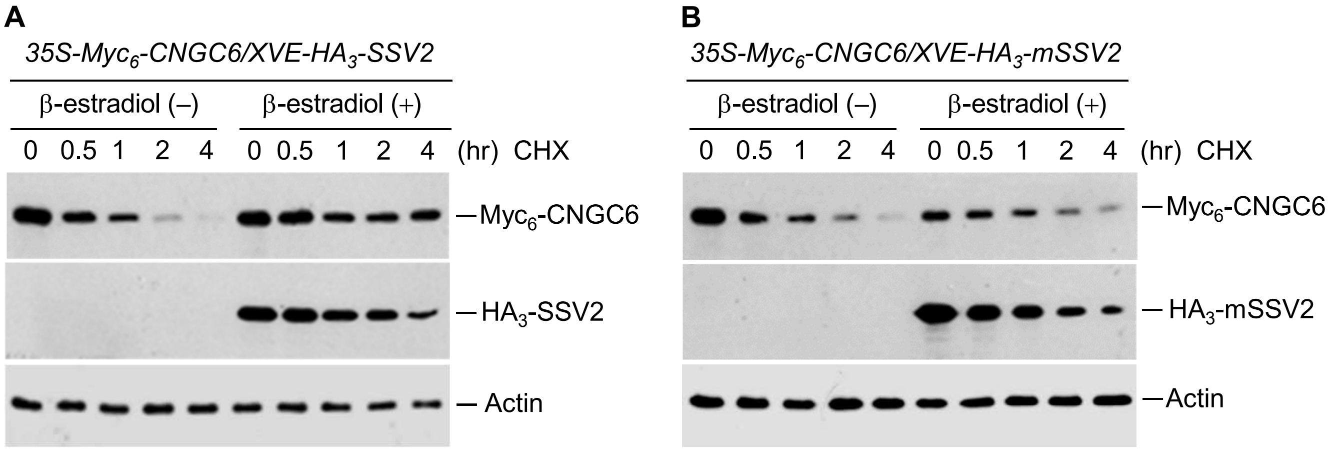

Supplemental Figure S8. CNGC6 is stabilized by SSV2 *in vivo*. Double transgenic plants expressing *35S-Myc$_6$-CNGC6* and *XVE-HA$_3$-SSV2* (**A**) or *35S-Myc$_6$-CNGC6* and *XVE-HA$_3$-mSSV2* (**B**) were incubated in a liquid medium with β-estradiol to induce SSV2 or mSSV2 expression. After incubation, the plants were washed and transferred to MS medium containing 100 μM cycloheximide (CHX). At specified time points, protein extraction was performed, and the samples were subjected to immunoblot analysis using anti-Myc and anti-HA antibodies. Actin was used as a loading control.

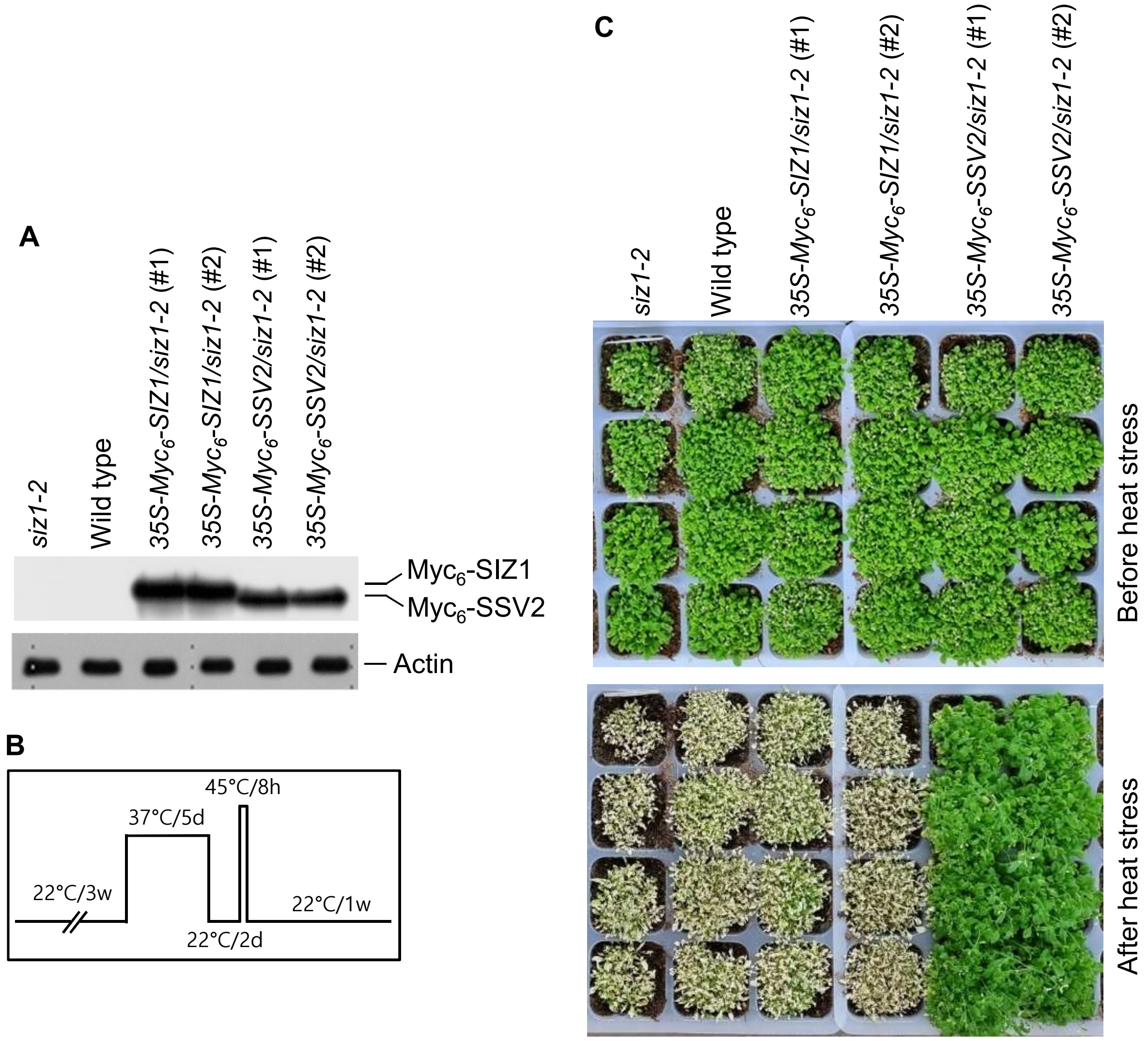

Supplemental Figure S9. Transgenic *siz1-2* mutant plants overexpressing *SSV2* under the control of the *35S* promoter are tolerant to heat stress. Three-week-old WT, *siz1-2*, and *35S-Myc$_6$-SIZ1/siz1-2* and *35S-Myc$_6$-SSV2/siz1-2* plants germinated and grown in soil were subjected to heat shock. (**A**) Examination of Myc$_6$-SIZ1 and Myc$_6$-SSV2 expression in *35S-Myc$_6$-SIZ1/siz1*-2 and *35S-Myc$_6$-SSV2/siz1-2* plants. Total protein was extracted from the leaves of 3-week-old WT, *siz1-2*, *35S-Myc$_6$-SIZ1/siz1-2*, and *35S-Myc$_6$-SSV2/siz1-2* plants. Following 10% SDS-PAGE, the Myc$_6$-SIZ1 and Myc$_6$-SSV2 proteins were detected by immunoblot analysis with anti-Myc antibody. Actin was used as a loading control. (**B**) Schematic representation of the heat shock treatment used in this study. d, day; w, week. (**C**) Photographs of plants taken 7 d after the heat treatment.

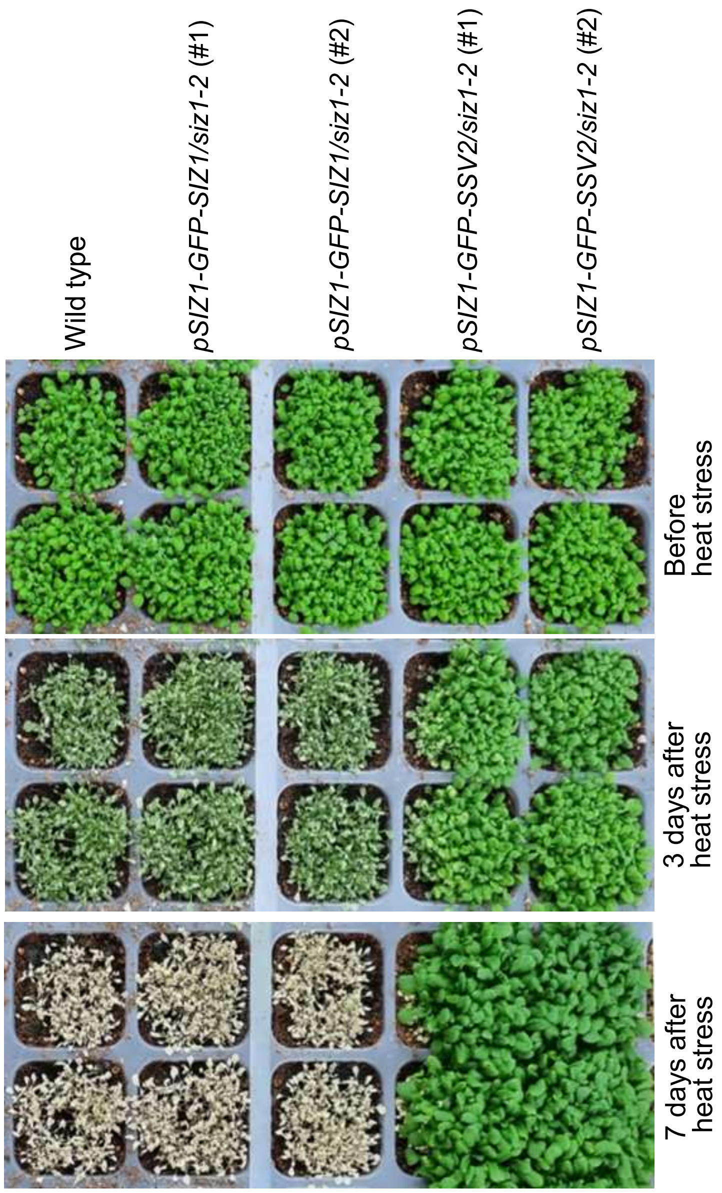

Supplemental Figure S10. Analysis of the heat tress tolerance of *pSIZ1-GFP-SSV2*/*siz1-2* plants. The WT, *pSIZ1-GFP-SIZ1*/*siz1-2*, and *pSIZ1-GFP-SSV2*/*siz1-2* seeds were germinated in soil, and plants were grown for 3 weeks. Phenotypes of WT and transgenic *siz1-2* plants were examined after treatment with different heat shock regimes. Plants were photographed 3 and 7 d after heat treatment.

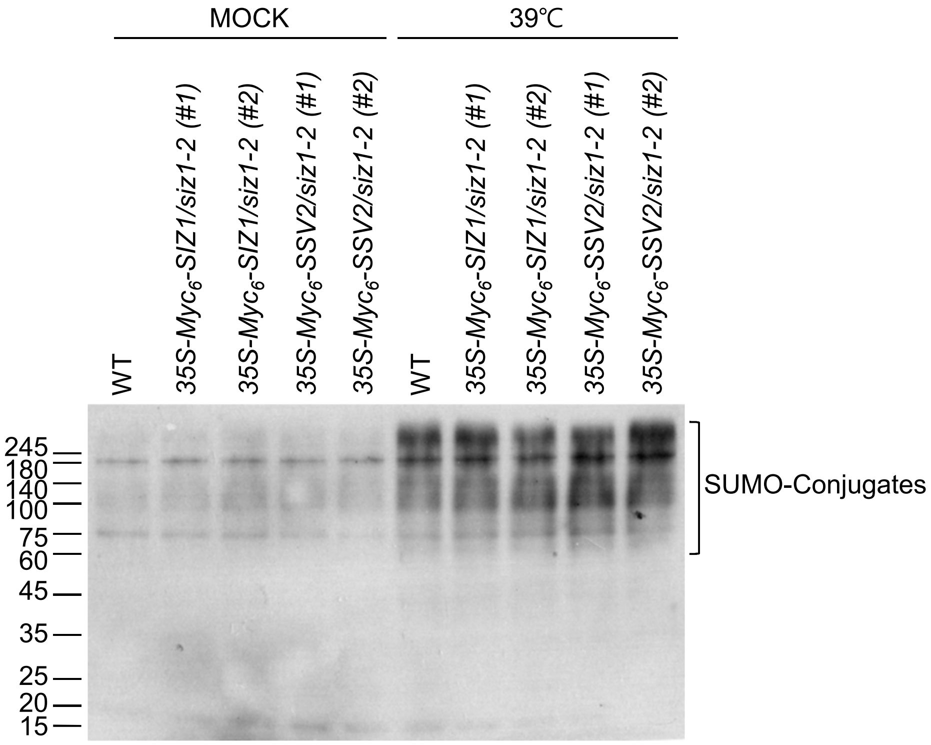

Supplemental Figure S11. SUMO-conjugates were examined in SSV2-expressing transgenic *siz1-2* plants under heat stress. WT, *35S-Myc$_6$-SIZ1/siz1-2*, and *35S-Myc$_6$-SSV2/siz1-2* plants were exposed to heat stress (39°C) for 15 min. SUMO-conjugates were detected through immunoblot analysis using an anti-AtSUMO1 antibody. Two independent transgenic *siz1-2* plants expressing Myc$_6$-SIZ1 or Myc$_6$-SSV2 were used for the assay.

A

MFDTCGPKGV KSQVISGQRE NFVRLDSMDS RYSQSSETGL NKCTLNIQGG PKRFAQGSKA SSGS**FKKG**FR 70
KGSEGLWSIG RSIGLGVSRA VFPEDLEVSE KKIFDPQDKF LLLCNKLFVA SCILAVSVDP LFLYLPFIND 140
KAKCVGIDRK LAIIVTTIRT VIDSFYLFHM ALRFRTAYVA PSSRVFGRGE LVIDPAQIAK RYLQQYFIID 210
LLSVLPVPQI IVWRFLYTSR GANVLATKQA LRYIVLVQYI PRFLRMYPLS SELKRTAGVF AETAWAGAAY 280
YLLLYMLASH IVGALWYLLA LERNNDCWSK ACHNNQNCTR NFLFCGNQNM KGYAAWDNIK VSYLQ**LKCP**V 350
NVPEDEEPPF DFGIYLRALS SGIVSSKNFV SKYFFCLWWG LQNLSTLGQG LETSTYPGEV IFSITLAIAG 420
LLLFALLIGN MQTYLQSLTI RLEEMRVKRR DSEQWMHHRM LPPELRERVR RYDQYKWLET RGVDEENLVQ 490
NLPKDLRRDI KRHLCLALVR RVPLFENMDE RLLDAICERL KPCLFTEKSY LVREGDPVNE MLFIIRGRLE 560
SVTTDGGRSG FYNRSL**LKEG** DFCGDELLTW ALDPKSGSNL PSSTRTVKAL TEVEAFALIA DELKFVASQF 630
RRLHSRQVQH TFRFYSQQWR TWAACFMQAA WRRYIKR**KKL** **E**QL**RKEE**EEE EAAAASVIAG GSPYSIRATF 700
LASKFAANAL RSVHKNRTAK STLLLSSTKE LVKFQKPPEP DFSAEDH 747

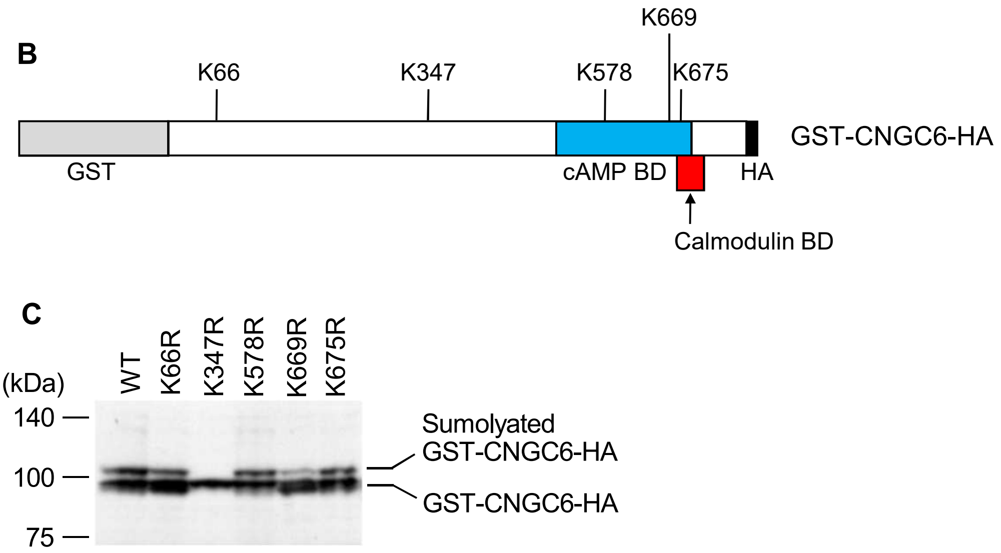

Supplemental Figure S12. Identification of the sumoylation site on CNGC6. (**A**) Deduced amino acid sequences of the CNGC6 protein. Five putative sumoylation sites (ψKXE) identified using the SUMOplot™ Analysis Program are indicated in bold type and underline. (**B**) Schematic diagram of the recombinant GST-CNGC6-HA protein. The cAMP binding domain (BD) and calmodulin BD, and putative sumoylation sites (K66, K347, K578, K669 and K675) are indicated. (**C**) Sumoylation assay for CNGC6 in *E. coli*. Sumoylation of GST-CNGC6-HA and K665R, K347R, K578R, K669R and K675R mutants was examined by immunoblot analysis with anti-HA antibody.

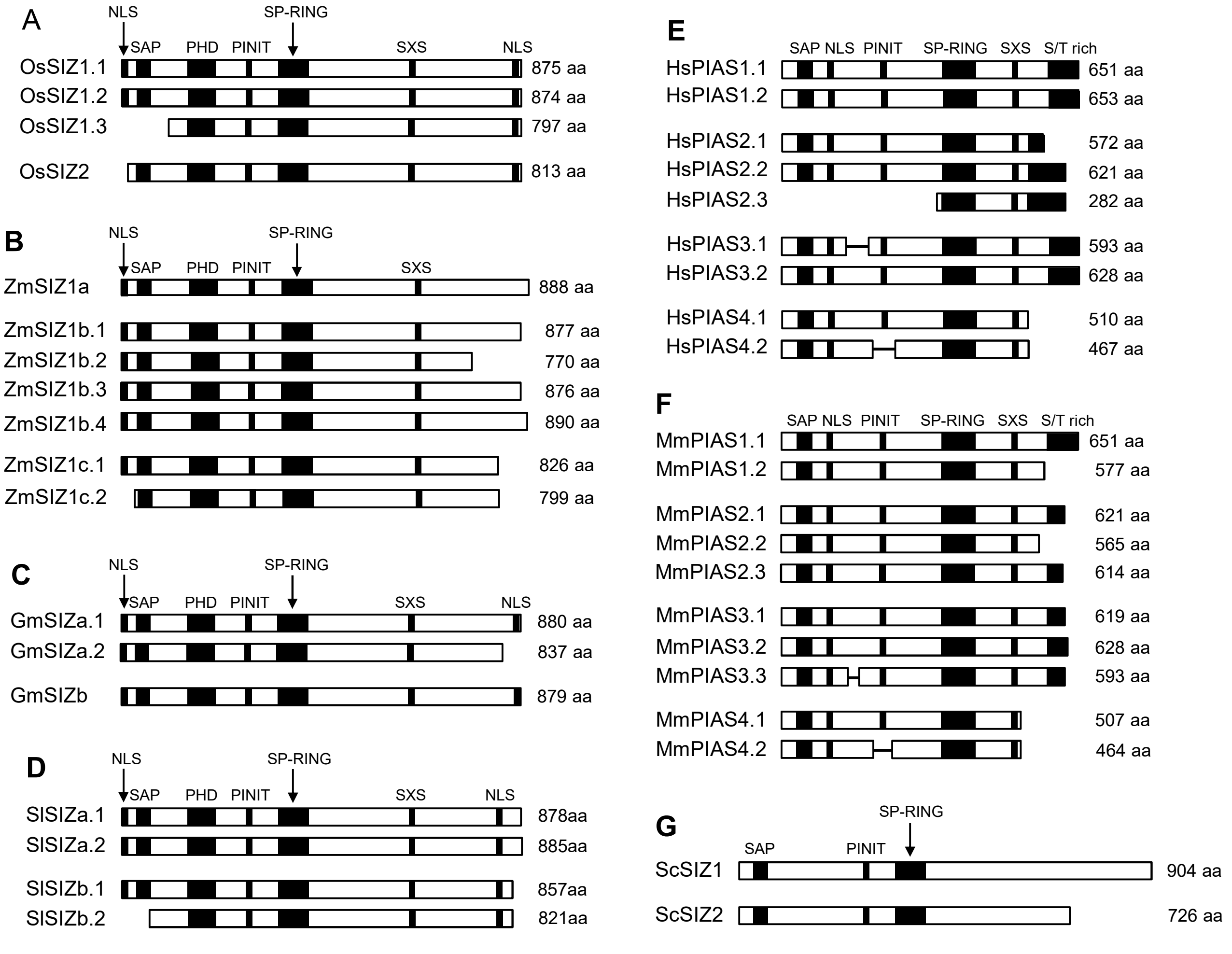

Supplemental Figure S13. Splicing variants of *Arabidopsis thaliana* SIZ1 orthologues in rice, corn, soybean, tomato, human, and mouse and yeast. Arabidopsis AtSIZ1 orthologues were analyzed by using currently available databases. **(A)** Two AtSIZ1 orthologues, OsSIZ1.1 and OsSIZ2, in rice (*Oryza sativa*). There are two OsSIZ1.1 splicing variants, OsSIZ1.2 and OsSIZ1.3, in rice. (**B**) Three AtSIZ1 orthologues, ZmSIZ1a, ZmSIZ1b and ZmSIZ1c.1, in corn (*Zea mays*). There is a ZmSIZ1c.1 splicing variant ZmSIZ1c.2 in corn. (**C**) Two AtSIZ1 orthologues, GmSIZa.1 and GmSIZb.1, in soybean (*Glycine max*). There is a GmSIZa.1 splicing variant GmSIZa.2 in soybean. (**D**) Two AtSIZ1 orthologues, SlSIZa.1 and SlSIZb.1, in tomato (*Solanum lycopersicum*. There is a SlSIZa.1 splicing variant SlSIZa.2 and a SlSIZb.1 splicing variant SlSIZb.2 in tomato. (**E**) Four AtSIZ1 orthologues, HsPIAS1.1, HsPIAS2.1, HsPIAS3.1 and HsPIAS4.1, in human (*Homo sapiens*). There are a HsPIAS1.1 splicing variant HsPIAS1.2, two HsPIAS2.1 splicing variants HsPIAS2.2 and HsPIAS2.2, a HsPIAS3.1 splicing variant HsPIAS3.2, and a HsPIAS4.1 splicing variant HsPIAS4.2 in human. (**F**) Four AtSIZ1 orthologues, MmPIAS1.1, MmPIAS2.1, MmPIAS3.1 and MmPIAS4.1, in mouse (*Mus musculus*). There a MmPIAS1.1 splicing variant MmPIAS1.2, two MmPIAS2.1 splicing variants MmPIAS2.2 and MmPIAS2.2, a MmPIAS3.1 splicing variant MmPIAS3.2, and a MmPIAS4.1 splicing variant MmPIAS4.2 in mouse. (**G**) Two AtSIZ1 orthologues, ScSIZ1 and ScSIZ2, in yeast (*Saccharomyces cerevisiae*). Splicing variant of ScSIZ1 and ScSIZ2 was not identified yet in yeast.

NLS, nuclear localization sequence; SAP, scaffold attachment factor A/B/acinus/PIAS; PHD, plant homeodomain; PINIT, Pro-Ile-Asn-Ile-Thr; SP-RING, Siz/PIAS Real interesting new gene; SXS, Ser-any amino acid-Ser; S/T rich, Ser/Thr rich.

Supplemental Table S1. List of primers used for this study.

Purpose	Gene	Vector	Forward primer	Reverse primer
**Real time qRT-PCR**	SIZ1	-	5’-CGGGATTAACTCAAGCCATC-3’	5’-TTACTCAGAATCCGAGTCAA-3’
	SSV2		5’-GGACCATGTTCTTGATGTTGC-3’	5’-AGCAACGACGCTGCAAATAC-3’
	CNGC6		5’-TCGCGTTAGTCCGAAGAGTT3’	5’-TACCGCCATCAGTGGTTACA-3’
	TUB		5’-CGAAAACGCTGACGAGTGTA-3’	5’-CCTTGGGAATGGGATAAGGT-3’
**RT-PCR**	pSIZ1-GFP-SIZ1 pSIZ1-GFP-SSV2	pEarleygate303	5’-ATGGTGAGCAAGGGCGAGGA-3’	5’-TCACTTAACTTTCAGATTACTGGTATCT-3’
	pSIZ1-Myc_6-SIZ1 pSIZ1-Myc_6-SSV2	pEarleygate303	5’-GTACAGCTCGTCCATGCCGTG-3’	5’-TCACTTAACTTTCAGATTACTGGTATCT-3’
***In vitro* sumoylation Assay**	SIZ1	pMAL-C2X	5’-atgcaggaattcatggatttggaagctaattgtaagg-3’	5’-atgcaggcggccgcaTTACTCAGAATCCGAGTCAA-3’
	SSV1		5’-atgcaggaattcatggatttggaagctaattgtaagg-3’	5’-atgcaggcggccgcaTTAAACTCCGGTGTCTTGTCTGA-3’
**Subcellular localization**	SIZ1	pBA002-GFP	5’-atgcaggaattcatggatttggaagctaattgtaagg-3’	5’-atgcaggcggccgcaTCAGACAGGGAACAAACCAG-3
	SSV2		5’-atgcaggaattcatggatttggaagctaattgtaagg-3	5’-atgcaggcggccgcaTTAAACTCCGGTGTCTTGTCTGA-3’
	SSV3		5’-atgcaggaattcatggatttggaagctaattgtaagg-3’	5’-atgcaggcggccgcaTCAATTCTTGCCTGCACAGAGAA-3’
	CNGC6		5’-atgcaggaattcATGTTCGATACTTGTGGCCC-3’	5’-atgcaggcggccgcatGTGATCTTCAGCAGAGAAATC-3’
	SIZ1-C1		5’-atgcaggaattcatggatttggaagctaattgtaagg-3’	5’-atgcagactagtTCATCCATGATTCCCAGAGGCG-3’
	SIZ1-C2		5’-atgcaggaattcatggatttggaagctaattgtaagg-3’	5’-atgcagctgcagTCAGACTTCATTATTTCCACCCTCG-3’
	SIZ1-CC1		5’-atgcaggaattcatggatttggaagctaattgtaagg-3’	5’-atgcagctgcagTCAGCGAGGAAATGAAAATGGATTATC-3’
	SIZ1-CC2		5’-atgcaggaattcatggatttggaagctaattgtaagg-3’	5’-atgcaggcggccgcTCACAGAAGCAACGACGCAGTC-3’
	SIZ1-A1		5’-atgcaggaattcatggatttggaagctaattgtaagg-3’	5’-atgcaggcggccgctcagacagggaacaaaccCTCAGAATCCGAGTCAA-3’
	SIZ1-N1		5’-atgcaggaattcGCTGATTTCTTCGGTGTCA-3’	5’-atgcaggcggccgcaTTACTCAGAATCCGAGTCAA-3’
	SIZ1-N2		5’- atgcagacgcgtaGAGGGTGGAAATAATGAA-3’	5’-atgcaggcggccgcaTTACTCAGAATCCGAGTCAA-3’
	SSV2-N1		5’- atgcaggaattcGCTGATTTCTTCGGTGTCA-3’	5’-atgcaggcggccgcaTCAGACAGGGAACAAACCAG-3’
	AtCML35		5’-aatgcagacgcgtaATGAAGCTCGCCGCTAGC-3’	5’-atgcaggcggccgcaCTAATGATGATGATCATTCATCGC-3’
	AtCML35-NLS		5’-aatgcagacgcgtaATGAAGCTCGCCGCTAGC-3’	5’-ATGAATGATCATCATCATGACAGTAGACAAGACAAGGCA-3’
	AtSYP122	pGWB554	5’-aatgcagGAATTCATGAACGATCTTCTCTCCGG-3’	5’-ATGAATgcggccgcatGCGTAGTAGCCGCCGAT-3

Supplemental Table S1. Continued.

**Yeast two hybrid assay**	SIZ1	pGAD424	5’-atgcaggaattcatggatttggaagctaattgtaagg-3’	5’-atgcagcccgggTTACTCAGAATCCGAGTCAA-3’
	SSV1		5’-atgcaggaattcatggatttggaagctaattgtaagg-3’	5’-atgcagcccgggTTAAACTCCGGTGTCTTGTCTGA-3’
	SSV2		5’-atgcaggaattcatggatttggaagctaattgtaagg-3’	5’-atgcagcccgggTCAGACAGGGAACAAACCAG-3’
	SSV3		5’-atgcaggaattcatggatttggaagctaattgtaagg-3’	5’-atgcagcccgggTCAATTCTTGCCTGCACAGAGAA-3’
	SSV3-D		5’-atgcaggaattcatggatttggaagctaattgtaagg-3’	5’-atgcagcccgggTTAAACGCTTCTGGCGAGGAAAT-3’
	AtSUMO1	pGBT8	5’-atgcaggaattcATGTCTGCAAACCAGGAGGA-3’	5’-atgcagctgcagTTAGCCACCAGTCTGATGGAGC-3’
	AtSUMO2		5’-atgcaggaattcATGTCTGCTACTCCGGAAGAA-3’	5’-atgcagctgcagTTAACCACCAGTCTGATGAAGCA-3’
	AtSUMO3		5’-atgcaggaattcATGTCTAACCCTCAAGATGACAA-3’	5’-atgcagctgcagTTAACCACCACTCATCGCCC-3’
	AtSUMO5		5’-atgcaggaattcATGGTGAGTTCCACAGACACA-3’	5’-atgcagctgcagTTAGCCACCACCAAGTTCCAT-3’
	AtSCE1		5’-atgcaggaattcATGGCTAGTGGAATCGCTCG-3’	5’-atgcaggtcgacGACAAGAGCAGGATACTGCTTG3’
**Production of transgenic plants**	XVE-SSV1	XVE	5’-GTCGACTCTAGCctcgagatggatttggaagctaattgtaagg-3’	5’-GTCGACTCTAGCctcgagatggatttggaagctaattgtaagg-3’
	XVE-HA_3-SSV2		5’-GTCGACTCTAGCctcgagatggatttggaagctaattgtaagg-3’	5’-AGGCCTGGATCGACTAGTTCAGACAGGGAACAAACCAG-3’
	XVE-SSV2		5’-GTCGACTCTAGCctcgagatggatttggaagctaattgtaagg-3’	5’-AGGCCTGGATCGACTAGTTCAGACAGGGAACAAACCAG-3’
	pCNGC6-CNGC6-GUS	pEarleyGate301	5’-aatcttttagcatgttaataaaatttaaca-3’	5’- atgcaggcggccgcatGTGATCTTCAGCAGAGAAATC -3’
	35S-Myc_6-SIZ1	pBA002-Myc_6	5’-atgcaggaattcatggatttggaagctaattgtaagg-3’	5’- atgcaggcggccgcaTTACTCAGAATCCGAGTCAA -3’
	35S-Myc_6-CNGC6		5’-atgcaggaattcATGTTCGATACTTGTGGCCC-3’	5’- atgcaggcggccgcatGTGATCTTCAGCAGAGAAATC -3’
	35S-Myc_6-SSV2		5’-atgcaggaattcatggatttggaagctaattgtaagg-3’	5’- atgcaggcggccgcaTCAGACAGGGAACAAACCAG -3’
	35S- HA_3-SSV2	pBA002-HA_3	5’-atgcaggaattcatggatttggaagctaattgtaagg-3’	5’- atgcaggcggccgcaTCAGACAGGGAACAAACCAG -3’
	pSIZ1-Myc_6-SIZ1	pEarleygate303	5’-CACCgcagacccttaaaaacagtgatg-3’	5’- atgcaggcggccgcaTTACTCAGAATCCGAGTCAA -3’
	pSIZ1-Myc_6-SSV2		5’-CACCgcagacccttaaaaacagtgatg-3’	5’- atgcaggcggccgcaTCAGACAGGGAACAAACCAG -3’
	pSIZ1-GFP-SIZ1	pEarleygate303	5’-atgcagggtaccATGGTGAGCAAGGGCGAGGA-3’	5’- atgcaggcggccgcaTTACTCAGAATCCGAGTCAA -3’
	pSIZ1-GFP-SSV2		5’-atgcagggtaccATGGTGAGCAAGGGCGAGGA-3’	5’- atgcaggcggccgcaTCAGACAGGGAACAAACCAG -3’
	35S-GFP-SSV2	pEarleygate104	5’-atgcaggaattcaatggatttggaagctaattgtaagg-3’	5’- atgcaggcggccgcaTCAGACAGGGAACAAACCAG -3’
**Site-Directed Mutagenesis**	CNGC6(K66R)		5’-aggAAAGGGTTTAGAAAAGGATCAGAGG-3’	5’- AAAAGATCCAGATGATGCTTTGCTT -3’
	CNGC6(K347R)		5’-aggTGCCCTGTCAATGTCCCC-3’	5’- TAGTTGAAGATACGAAACTTTAATATTGTCCC -3’
	CNGC6 (K578R)		5’-aggGAAGGAGATTTCTGCGGTGACGA-3’	5’- AAGTAAACTCCGGTTGTAGAATCCGCT -3’
	CNGC6 (K669R)		5’-aggTTGGAGCAACTTAGAAAAGAAGAGGA-3’	5’- CTTCCTCTTTATGTACCGTCGC -3’
	CNGC6 (K675R)		5’-aggGAAGAGGAAGAAGAAGAAGCGG-3’	5’- TCTAAGTTGCTCCAATTTCTTCCTC -3’
	SSV2(C379S)		5’-TCTGTGCACATGGGCTGTTTTGAC-3’	5’- GGGTAAAAATCTCCCAGCAACT -3’
	SSV2(W400A)		5’-GCTCAGTGCCCTATTTGTCTGAAGAAC-3’	5’- CTTTCTGGAACGTTGATTCAACTC-3’
**BiFC**	CNGC6	pBA3130	5’-atgcaggaattcATGTTCGATACTTGTGGCCC-3’	5’-atgcaggcggccgcatGTGATCTTCAGCAGAGAAATC-3’

		pBA3132		
		pBA3134		
		pBA3136		
	SSV2	pBA3130	5’-atgcaggaattcatggatttggaagctaattgtaagg-3’	5’- atgcagcccgggTCAATTCTTGCCTGCACAGAGAAC-3’
		pBA3132		
		pBA3134		
		pBA3136		

* Restriction endonuclease sites were indicated by underlines.
