# Supplementary material for: E3 SUMO ligase SIZ1 splicing variants localize and function according to external conditions
Source: Plant Physiol. 2024 Mar 18;195(2):1601–23. doi: 10.1093/plphys/kiae108 (PMC11142376; doi:10.1093/plphys/kiae108)
Supplement: kiae108_Supplementary_Data [file pp.01987.2023-s03.pdf › pp.01987.2023-s03.pdf]

Plant Physiology®

Role of Arabidopsis E3 SUMO ligase SIZ1 splicing variants: From localization to function

Jun Soo Kwak, Jong Tae Song, and Hak Soo Seo

Review Timeline:

Submission Date:	12-Oct-2022
Editorial Decision:	21-Nov-2022
Resubmission Received:	13-Dec-2023
Editorial Decision:	02-Jan-2024
Revision Received:	08-Jan-2024
Accepted:	08-Jan-2024

21-Nov-2022

Prof. Dr. Hak Soo Seo
Seoul National University
Plant Science
Seoul 08826
Korea (South), Republic of

RE: Role of Arabidopsis E3 SUMO ligase SIZ1 splicing variants: From localization to function

Dear Dr. Hak Soo Seo:

Thank you for giving us the opportunity to review your manuscript for publication in the Plant Physiology. The reviewers have found the manuscript of interest, but have raised a number of concerns that will require your attention. As these concerns are of some gravity and will entail substantial changes to the manuscript, you are likely to need additional time to address them. I must therefore reject the manuscript in its present form. Nonetheless, if you are able to address the points raised in review, I would welcome your resubmission of a manuscript that incorporates your responses.

In particular, I direct your attention to the following comments for consideration in a resubmitted manuscript.

I agree that the use of SSV2 mutants that decrease SUMO E3 ligase activity and further address their capacity to increase CNGC6 stability and thermotolerance should very interesting to strengthen the message of the work.

Similarly, the study of siz1-2 mutant plants complemented with SSV2 or SIZ1 would be very valuable.
Discuss the phenotype of transgenic plants over expressing SSV2.
Explain the GUS assay more clearly.
Please, also answer all the points raised by the 2 referees.

Please note that any resubmission will be treated as a new entry to the system and is likely to go out for review.

If you do have any queries or concerns, you are welcome to contact me in the first instance.

Thank you again for allowing Plant Physiology to review your work. We look forward to hearing from you soon.

Sincerely,

Plant Physiology Board of Editors

-- Reviewer comments:
Reviewer #1 (Comments for the Author):

The manuscript with the title "Role of Arabidopsis E3 SUMO ligase SIT1 splicing variants: From localization to function" presents a set of data with wide implications for stress response mechanisms. It is a significant step forward in deciphering mechanisms that adapt plants to higher temperatures, and the role SUMO plays in this process.

The manuscript is well written and uses standard methods with high reproducibility, so that judgement on the quality is straightforward.

Fig. 8: A western blot using anti GUS antibodies would be more convincing than tissue staining. Furthermore, the experiments as described in the methods part use the vectors "pET8" (should be pER8, presumably) and pMDC163 to bring two DNA pieces into Arabidopsis. However, both vectors have the same marker Hyg resistance. How did that work ? What was the percentage of plants containing both inserts versus only one of the two ? Generally, the claim that CNGC6 protein levels are higher upon SSV2 increase needs better documentation.

Minor:

Please mention in the first paragraph that SSV5 will be introduced later in the text. It is irritating that this splice variant is part of the abstract, but then not mentioned when the other, previously known splice variants are introduced.

Fig. S2 shows that, upon osmotic shock and subsequent detachment of the plasma membrane from the cell wall, the GFP-SSV2

protein signal does not stain the cell wall. More relevant would be an answer to the question whether SSV2 is cytoplasmic vs. membrane-associated. Do the authors have images from cells with small vacuoles (e.g., from transgenic Arabidopsis plants), so that cytoplasmic localization of the protein can be assessed ?

Fig. 9 and 10 are very similar. Please put one of the two (Fig. 9 ?) into the supplement.

Source of the anti SIZ1 antibody is not revealed.

Reviewer #2 (Comments for the Author):

There is currently a growing interest towards the study of plant post-translational modifications and their effects on plant development and response to biotic or abiotic stresses. In Arabidopsis, protein SUMOylation relies on only a handful of SUMO E3 ligases including SIZ1 which is the main SUMO E3 responsible for the SUMOylation wave that follows heat stress exposure.

In this context, the manuscript by Kwak et al. reports the cellular localization of the splice isoforms of SIZ1. They show that while most of the splice variants of SIZ1 localize predominantly to the nucleus, two splice variants coding for identical protein sequences, SSV2 and SSV5, also localize to the cell membrane. Their localization data supports the existence of a NLS in the C-terminal part of SIZ1 but not in SSV2/5. They further show using in vitro SUMOylation assays that both SIZ1 and all its splice variants are competent for SUMO conjugation, which is expected as the domains responsible for this activity are present in all proteins. They used bimolecular fluorescence complementation assays to identify an interaction between SSV2 and CNGC6, an ion channel implicated in the stress response. They finally show that plants expressing SSV2 either under the control of its native promoter or a 35S promoter are more thermotolerant than wild-type, SIZ1-expressing or siz1-2 mutant plants. They propose a model where SSV2 interacts and SUMOylate CNGC6 to regulate plant heat stress response. Overall, the discovery of stress-induced variants of SIZ1 that SUMOylate a membrane protein to increase thermotolerance is both intriguing and novel. The manuscript is well written and well presented. However, I have a few technical and conceptual concerns.

Major concerns:

1. Although the authors suggest a model where SSV2 SUMOylates CNGC6 to increase its stability and plant thermotolerance, there is a real possibility that those processes occur in a SUMOylation-independent manner. Indeed, most of the SUMOylation occurs in the nucleus. For example, some of the other components of the SUMOylation pathways are mostly nuclear (e.g. Castaño-Miquel et al. Molecular Plant, 2013 for the localization of E1 in Arabidopsis). Mass spectrometry data also confirms that most of SUMOylation targets are nuclear in plants and in other organisms. Furthermore, CNGC6 does not appear in a recent proteomic study that detected ~1000 SUMOylated proteins in Arabidopsis. In my opinion, the authors should determine whether the stabilization of CNGC6 by SSV2 and thermotolerance are SUMOylation-dependent or SUMOylation-independent. This could be done by using SSV2 mutants that decrease its SUMO E3 ligase activity and assess the capacity of those mutants to increase CNGC6 stability and plant thermotolerance. Such mutants have been abundantly used in other systems (for example, the mutants W372A and/or C351S of PIAS1, a SIZ1 homolog).

2. SIZ1 is currently viewed as the main SUMO E3 ligase responsible for rapid SUMOylation of multiple proteins following heat-stress exposure (Saracco et al. Plant Phys, 2007 and Rytz et al. Plant Cell, 2018). Given the proposed role of SSV2 in such heat-stress response, it would be important to compare the general heat-stress-induced protein SUMOylation response in siz1-2 mutant plants complemented with SSV2 or SIZ1.

3. The authors should discuss whether the existence of SIZ1 homologs lacking NLS is unique to Arabidopsis or if it is conserved in other plants or in other species. For example, is there RNA-Seq data or NLS prediction in other plants that support the existence of SIZ1 variants located to the plasma membrane?

4. I am surprised that the transgenic plants expressing SSV2 (under the control of native or 35S protomoter) display such a clear phenotype while there seems to be little difference in the thermotolerance of siz1-2, wild-type and SIZ1 expressing plants. This looks more like a gain-of-function of the SSV2-expressing plants than a rescue experiment. Could the authors discuss the causes of such differences?

Minor concerns:

1. Please indicate the number of replicates made for each experiment.

2. Please provide the catalogue number for all the antibodies used.

3. Why is the size of MBP-SSV2 about 150 kDa in Figure 7C while it is around 110 kDa in Figure 4B? Please check the position of the protein ladder.

4. Page 12: "SSZ1 and all SSVs exhibit E3 ubiquitin ligase activity": replace "ubiquitin" by "SUMO"

5. In the legend of Figure 6, replace "qRT-PCR" by "RT-PCR".

6. In the legend of Figure 7, replace "HYP2" by "HPY2"

7. In the legend of Figure 8, there is a work lacking between "were" by "with β-estradiol".

--- Reviewer comments:

Reviewer #1 (Comments for the Author):

The manuscript with the title "Role of Arabidopsis E3 SUMO ligase SIT1 splicing variants: From localization to function" presents a set of data with wide implications for stress response mechanisms. It is a significant step forward in deciphering mechanisms that adapt plants to higher temperatures, and the role SUMO plays in this process.

The manuscript is well written and uses standard methods with high reproducibility, so that judgement on the quality is straightforward.

Fig. 8: A western blot using anti GUS antibodies would be more convincing than tissue staining. Furthermore, the experiments as described in the methods part use the vectors “pET8” (should be pER8, presumably) and pMDC163 to bring two DNA pieces into Arabidopsis. However, both vectors have the same marker Hyg resistance. How did that work? What was the percentage of plants containing both inserts versus only one of the two?

Generally, the claim that CNGC6 protein levels are higher upon SSV2 increase needs better documentation.

(Response to reviewer)

Thank you for your comment. According to your suggestion, we attempted to detect the recombinant protein CNGC6-GUS multiple times by western blotting using anti-GUS antibody. However, under our experimental conditions, we were unable to detect the CNGC6-GUS recombinant protein using the anti-GUS antibody. Unfortunately, we did not obtain clear results, and we are uncertain about the reason for this. We cautiously speculate that it may be due to the low expression level of the *CNGC6* gene under the *CNGC6* promoter and the relatively large size of the recombinant protein CNGC6-GUS (theoretically calculated size of CNGC6-GUS is approximately 165.2 kDa). Therefore, we evaluated GUS enzyme activity by using total soluble proteins extracted from the roots of transgenic plants treated with β-estradiol. We have included the corresponding data as Fig. 9C. The results have been described in the “Results” section and discussed in the “Discussion” section.

We apologize for the mistake and have corrected “pET8” to “pER8” in the “Plant transformation and GUS staining assay” section of the Materials and Methods. We are also sorry for the error related to “pMDC163”. It was mislabeled. We used the “pEarleyGate 301” vector for the cloning of the CNGC6 promoter. Therefore, we have corrected “pMDC163” to “pEarleyGate 301” in the “Plant transformation and GUS staining assay” section of the Materials and Methods.

We further investigated the impact of SSV2 on CNGC6 decay by treating transgenic plants harboring *35S-*Myc_6*-CNGC6* and *XVE-*HA_3*-SSV2*, or *35S-*Myc_6*-CNGC6* and *XVE-*HA_3*-mSSV2*. After inducing SSV2 or mSSV2 expression, we assessed the decay rate of Myc_6-CNGC6 following cycloheximide treatment. mSSV2 represents a variant of SSV2 in which specific residues (Cysteine 379 and Tryptophan 400) within the RING motif have been mutated to serine and alanine (C379S, W400A). Our findings revealed a delay in the degradation of Myc_6-CNGC6 in SSV-expressing plants compared to non-treated plants (Supplemental Fig. S8A). However, the degradation rate of Myc_6-CNGC6 remained similar in both non-treated and mSSV2-expressing plants (Supplemental Fig. S8B), indicating that CNGC6 is stabilized by SSV2. Nevertheless, in accordance with your suggestion, we modified the sentence to avoid expressions such as “CNGC6 protein levels are higher upon SSV2 increase”. For instance, we changed the sentence “CNGC6 stabilization by SSV2” to “CNGC6 stabilization in the presence of SSV2”.

Minor:

Please mention in the first paragraph that SSV5 will be introduced later in the text. It is irritating that this splice variant is part of the abstract, but then not mentioned when the other, previously known splice variants are introduced.

(Response to reviewer)

Thank you for your comment. Following your advice, we have excluded the term "SSV5" from the "Abstract" section to prevent confusion among readers. Additionally, whenever we referred to the SSV2 protein in the manuscript, we replaced "SSV2/SSV5" with "SSV2". We appreciate your input once again.

Fig. S2 shows that, upon osmotic shock and subsequent detachment of the plasma membrane from the cell wall, the GFP-SSV2 protein signal does not stain the cell wall. More relevant would be an answer to the question whether SSV2 is cytoplasmic vs. membrane-associated. Do the authors have images from cells with small vacuoles (e.g., from transgenic Arabidopsis plants), so that cytoplasmic localization of the protein can be assessed?

(Response to reviewer)

Thank you for your comment. Following your suggestion, we conducted further investigations into the localization of SSV2 in the cell membrane. To carry out this experiment, we initially generated transgenic *siz1-2* plants containing the *35S-HA$_3$-SSV2* construct. Subsequently, we isolated nuclear, cytosolic, and plasma membrane fractions from the transgenic plants and extracted total proteins from each fraction. Finally, we conducted immunoblotting to determine the presence of HA$_3$-SSV2 in the plasma membrane. Our findings revealed that HA$_3$-SSV2 was primarily detected in the plasma membrane fraction and, to a lesser extent, in the nuclear fraction, while it was not present in the cytosolic fraction. These results are consistent with the observations depicted in Fig. 2D. Histone 3 (a nucleus-specific protein), tubulin (a cytosol-specific protein), and H+-ATPase (a plasma membrane-specific protein) were specifically identified in the nuclear, cytosolic, and plasma membrane fractions, respectively. Therefore, these findings further confirm that SSV2 localizes within the cell membrane and not in the cytosol or cell wall.

We included the corresponding data as Supplemental Fig. S2C.

Additionally, in the "Results" section under "SSV2 associates with the cell membrane but not with the cell wall", we detailed our investigation into SSV2 localization as follows. "We further sought to confirm the localization of SSV2 in the cell membrane. For this purpose, we isolated nuclear, cytosolic and plasma membrane fractions from transgenic *siz1-2* plants containing the *35S-HA$_3$-SSV2* construct and extracted total proteins from these fractions. HA$_3$-SSV2 was assessed through immunoblotting using an anti-HA antibody. The results demonstrated that HA$_3$-SSV2 was primarily detected in the plasma membrane fraction and, to a lesser extent, in the nuclear fraction, but it was not present in the cytosolic fraction, consistent with the findings depicted in Fig. 2D (Supplemental Fig. S2C). Histone 3, a nuclear marker protein, was exclusively detected in the nuclear fraction, whereas tubulin, a cytosol marker protein, was solely detected in the cytosolic fraction. Additionally, H^+-ATPase, a plasma membrane marker protein, was exclusively detected in the plasma membrane fraction. Therefore, these data (Supplemental Fig. S2A-S2C) indicate that SSV2 mainly localizes in the cell membrane but not in the cytosol and cell wall".

Fig. 9 and 10 are very similar. Please put one of the two (Fig. 9?) into the supplement. (Response to reviewer)

(Response to reviewer)

Thank you for your comment. According to your suggestion, we have moved Figure 9 to the Supplemental Data, and it is now designated as Supplemental Fig. S9.

Source of the anti SIZ1 antibody is not revealed.

(Response to reviewer)

We are sorry for this mistake. We obtained the anti-AtSIZ1 antibody from Dr. Nam-Hai Chua laboratory. We thus described the source of the antibody in the "Acknowledgements" section as follows.

"Anti-AtSIZ1 antibody was kindly provided by Dr. Nam-Hai Chua at Rockefeller University".

Reviewer #2 (Comments for the Author):

There is currently a growing interest towards the study of plant post-translational modifications and their effects on plant development and response to biotic or abiotic stresses. In Arabidopsis, protein SUMOylation relies on only a handful of SUMO E3 ligases including SIZ1 which is the main SUMO E3 responsible for the SUMOylation wave that follows heat stress exposure.

In this context, the manuscript by Kwak et al. reports the cellular localization of the splice isoforms of SIZ1. They show that while most of the splice variants of SIZ1 localize predominantly to the nucleus, two splice variants coding for identical protein sequences, SSV2 and SSV5, also localize to the cell membrane. Their localization data supports the existence of a NLS in the C-terminal part of SIZ1 but not in SSV2/5. They further show using in vitro SUMOylation assays that both SIZ1 and all its splice variants are competent for SUMO conjugation, which is expected as the domains responsible for this activity are present in all proteins. They used bimolecular fluorescence complementation assays to identify an interaction between SSV2 and CNGC6, an ion channel implicated in the stress response. They finally show that plants expressing SSV2 either under the control of its native promoter or a 35S promoter are more thermotolerant than wild-type, SIZ1-expressing or siz1-2 mutant plants. They propose a model where SSV2 interacts and SUMOylate CNGC6 to regulate plant heat stress response. Overall, the discovery of stress-induced variants of SIZ1 that SUMOylate a membrane protein to increase thermotolerance is both intriguing and novel. The manuscript is well written and well presented. However, I have a few technical and conceptual concerns.

Major concerns:

Although the authors suggest a model where SSV2 SUMOylates CNGC6 to increase its stability and plant thermotolerance, there is a real possibility that those processes occur in a SUMOylation-independent manner. Indeed, most of the SUMOylation occurs in the nucleus. For example, some of the other components of the SUMOylation pathways are mostly nuclear (e.g. Castaño-Miquel et al. Molecular Plant, 2013 for the localization of E1 in Arabidopsis). Mass spectrometry data also confirms that most of SUMOylation targets are nuclear in plants and in other organisms. Furthermore, CNGC6 does not appear in a recent proteomic study that detected ~1000 SUMOylated proteins in Arabidopsis. In my opinion, the authors should determine whether the stabilization of CNGC6 by SSV2 and thermotolerance are SUMOylation-dependent or SUMOylation-independent. This could be done by using SSV2 mutants that decrease its SUMO E3 ligase activity and assess the capacity of those mutants to increase CNGC6 stability and plant thermotolerance. Such mutants have been abundantly used in other systems (for example, the mutants W372A and/or C351S of PIAS1, a SIZ1 homolog).

(Response to reviewer)

Thank you for your comment. We agree with you. Until now, it has been known that the sumoylation machinery primarily localizes in the nucleus, where most sumoylation processes take place. By the way, it has been reported that the SUMO-conjugating enzyme is present in the plasma membrane. Furthermore, Ubc-9 has been observed to sumoylate K2P1, a potassium channel protein, specifically on an intracellular lysine at position 274 (Rajan et al., 2005, Sumoylation silences the plasma membrane leak K+ Channel K2P1. *Cell*, 121: 37-47), demonstrating the operation of the SUMO pathway at the plasma membrane and its role in regulating ion channel function.

In *S. cerevisiae*, where SUMO-activating, -conjugating, and cleavage enzymes were initially characterized, substrates include septins, proteins crucial for cell separation (Johnson and Blobel, 1999, Cell cycle-regulated attachment of the ubiquitin-related protein SUMO to the yeast septins. *J. Cell Biol.* 147: 981-994). These septins localize near the plasma membrane at the neck between mother and daughter cells, coinciding with the presence of Siz1, an E3 factor that associates with Ubc-9 to confer substrate specificity (Johnson and Gupta, 2001, An E3-like factor that promotes SUMO conjugation to the yeast septins. *Cell*, 106: 735-744). Additionally, suggestive findings include the identification of Ubc-9 as a binding partner in yeast two-hybrid screens involving the epithelial sodium channel ENaC (Malbert-Colas et al., 2003, Identification of new partners of the epithelial sodium channel alpha subunit. *C. R. Biol*. 326: 615-624) and the intracellular glucose transporter GLUT4 (Giorgino et al., 2000, The sentrin-conjugating enzyme mUbc9 interacts with GLUT4 and GLUT1 glucose transporters and regulates transporter levels in skeletal muscle cells. *Proc. Natl. Acad. Sci. USA*. 97: 1125-1130).

More recently, it has been reported that ion channels undergo SUMO modification, thereby regulating their functions (Benson et al., 2017, SUMO modification of ion channels. *Adv Exp Med Biol*. 963:127-141). These findings suggest that sumoylation can occur not only in the nucleus but also in the cytosol and plasma membrane within the plant system.

Anyhow, based on your suggestion, we examined the effect of SSV2 on the stability of CNGC6 and the role of sumoylation of CNGC6 on heat tolerance.

We first examined whether CNGC6 is modified with SUMO by SSV2. To do this, we co-infiltrated *35S-Myc$_6$-CNGC6*, *35S-GFP-SSV2*, and *35S-HA$_3$-AtSUMO1-GG* constructs into the leaves of *Nicotiana benthamiana*. After confirming of the expression of the recombinant proteins, Myc$_6$-CNGC6 and GFP-SSV2, the sumoylation of CNGC6 was examined by western blotting with anti-Myc antibody following immunoprecipitation (IP) with anti-HA antibody. We found that sumoylated CNGC6 was only observed in the sample infiltrated with *35S-GFP-SSV2*, indicating that CNGC6 is sumoylated by E3 ligase activity of SSV2. We included these data as Fig. 8. The results have been described in the "Results" section and discussed in the "Discussion" section.

Next, we identified the sumoylation site of CNGC6. CNGC6 contains five potential sumoylation sites (ΨKXE) located at lysine 66 (K66), lysine 347 (K347), lysine 578 (K578), lysine 669 (K669), and lysine 675 (K675; Supplemental Fig. S12A and B). To determine the specific sumoylation sites on the CNGC6 protein, we generated single mutant derivatives with the mutations K66R, K347R, K578R, K669R, and K675R. These mutant proteins were overexpressed in *E. coli*, purified using glutathione affinity columns, and utilized for *in vitro* sumoylation assays. The results demonstrated that GST-mCNGC6-HA (K66R), GST-mCNGC6-HA (K578R), GST-mCNGC6-HA (K669R), and GST-mCNGC6-HA (K675R) underwent sumoylation, while GST-mCNGC6-HA (K347R) did not, indicating that K347 is the principal site of SUMO conjugation on CNGC6 (Supplemental Fig. S12C). We included these data as Supplemental Fig. S12, with the results detailed in the "Results" section and further discussed in the "Discussion" section.

After identification of sumoylation site of CNGC6, we further examined heat tolerance of transgenic *cngc6* mutants overexpressing mutant CNGC6 proteins. For the experiment, we generated transgenic *cngc6* plants overexpressing CNGC6-Myc$_6$, mCNGC6(K66R)-Myc$_6$, mCNGC6(K347R)-Myc$_6$,

mCNGC6(K578R)-Myc$_6$, mCNGC6(K669R)-Myc$_6$, and mCNGC6(K675R)-Myc$_6$ using the corresponding constructs*35S-CNGC6-Myc$_6$*, *35S-mCNGC6(K66R)-Myc$_6$*, *35S-mCNGC6(K347R)-Myc$_6$*, *35S-mCNGC6(K578R)-Myc$_6$*, *35S-mCNGC6(K669)-Myc$_6$*, and *35S-mCNGC6(675R)-Myc$_6$* and then investigated heat tolerance of them. We found that transgenic *cngc6* plants overexpressing mCNGC6(K347R)-Myc$_6$ were sensitive to heat stress, similar to the *cngc6* mutants. However, other transgenic *cngc6* plants overexpressing CNGC6-Myc$_6$, mCNGC6(K66R)-Myc$_6$, mCNGC6(K578R)-Myc$_6$, mCNGC6(K669R)-Myc$_6$, and mCNGC6(K675R)-Myc$_6$ exhibited restored heat tolerance to similar to wild type, indicating that sumoylation is necessary for the role of CNGC6 in heat tolerance. We included these data as Fig. 12. Additionally, the results have been described in the "Results" section and discussed in the "Discussion" section.

In addition, to further validate the role of SSV2 in stabilizing CNGC6, we conducted an experiment to examine the impact of SSV2 on the decay of CNGC6. To do this, we generated double transgenic plants expressing *35S-Myc$_6$-CNGC6* along with *XVE-HA$_3$-SSV2 or 35S-Myc$_6$-CNGC6* along with *XVE-HA$_3$-mSSV2*. Subsequently, we treated these transgenic plants with β-estradiol to induce the expression of SSV2 or mSSV2. Following induction, we exposed the transgenic plants to cycloheximide (CHX) to inhibit new protein synthesis, and then we assessed the decay rate of Myc$_6$-CNGC6. Our results revealed that the degradation of Myc$_6$-CNGC6 was delayed in plants co-expressing SSV2 (Supplementary Fig. S8A), while the degradation rate of Myc$_6$-CNGC6 remained similar in both untreated plants and those expressing mSSV (Supplementary Fig. S8B). These findings indicate that CNGC6 is stabilized in the presence of SSV2. We have included these data as Supplemental Fig. S8, and the results have been detailed in the "Results" section and further discussed in the "Discussion" section.

2. SIZ1 is currently viewed as the main SUMO E3 ligase responsible for rapid SUMOylation of multiple proteins following heat-stress exposure (Saracco et al. Plant Phys, 2007 and Rytz et al. Plant Cell, 2018). Given the proposed role of SSV2 in such heat-stress response, it would be important to compare the general heat-stress-induced protein SUMOylation response in siz1-2 mutant plants complemented with SSV2 or SIZ1.

(Response to reviewer)

Thank you for your comment.

According to your suggestion, we assessed the levels of SUMO-conjugates in WT, *35S-Myc$_6$-SIZ1/siz1-2*, and *35S-Myc$_6$-SSV2/siz1-2* transgenic plants under heat stress conditions. Interestingly, we observed that the levels of SUMO-conjugates remained similar in WT, *35S-Myc$_6$-SIZ1/siz1-2*, and *35S-Myc$_6$-SSV2/siz1-2* transgenic plants, despite an overall increase after the heat stress treatment.

Based on these results, we cautiously hypothesize that the levels of sumoylated forms of specific target proteins regulated by SIZ1 or SSV2 may vary in WT, *35S-Myc$_6$-SIZ1/siz1-2*, and *35S-Myc$_6$-SSV2/siz1-2* transgenic plants. SSV2 can sumoylate its target proteins located in the cytoplasmic membrane, whereas SIZ1 does not influence the sumoylation of membrane-anchored or localized proteins. Additionally, since SSV2 is also present in the nucleus and can partially compensate for the functions of SIZ1, nuclear-localized target proteins might be sumoylated in both *35S-Myc$_6$-SIZ1/siz1-2* and *35S-Myc$_6$-SSV2/siz1-2* transgenic plants. Therefore, the sumoylated proteins in *35S-Myc$_6$-SSV2/siz1-2* transgenic plants might differ from those in *35S-Myc$_6$-SIZ1/siz1-2* transgenic plants, even though they cannot be distinguished in immunoblot data. It is also possible that sumoylated proteins in WT plants could differ from those in *35S-Myc$_6$-SIZ1/siz1-2* or *35S-Myc$_6$-SSV2/siz1-2* transgenic plants due to higher SIZ1 or SSV2 levels in the transgenic plants, although these distinctions cannot be observed in immunoblot data. In conclusion, we cautiously infer that the sumoylation levels of SIZ1- or SSV2-specific target proteins might vary in specific subcellular regions, despite the total SUMO-conjugate levels detected by immunoblotting appearing similar in WT, *35S-Myc$_6$-SIZ1/siz1-2*, and *35S-Myc$_6$-SSV2/siz1-2* transgenic plants.

In any case, we discussed the possible reasons in "Discussion" section as follows.

"Despite the increased heat tolerance observed in SSV2-expressing transgenic *siz1-2* plants, there was no difference in the levels of sumoylated proteins among WT and *siz1-2* plants expressing SIZ1 or SSV2 (Supplemental Fig. S11). Due to the localization of SSV2 and SIZ1, SSV2 can sumoylate its target proteins distributed in the cytoplasmic membrane, while SIZ1 cannot influence the sumoylation of membrane-anchored or localized proteins. SSV2 is also partially localized in the nucleus, suggesting that nuclear-targeted proteins can be sumoylated in SSV2-expressing *siz1-2* plants. Consequently, sumoylated proteins in SSV2-expressing *siz1-2* plants may differ from those in SIZ1-expressing *siz1-2* plants, although these differences cannot be distinguished in immunoblot data. Additionally, sumoylated proteins in the WT may also differ from those in SIZ1-expressing *siz1-2* or SSV2-expressing *siz1-2* plants due to the higher levels of SIZ1 or SSV2 in the transgenic plants, even though these differences are not apparent in the immunoblot data. Therefore, we cautiously infer that the sumoylation levels of SIZ1- or SSV2-specific target proteins may vary in specific subcellular regions, despite the similar levels of total SUMO-conjugates detected by immunoblotting in WT and SIZ1- or SSV2-expressing *siz1-2* plants."

3. The authors should discuss whether the existence of SIZ1 homologs lacking NLS is unique to Arabidopsis or if it is conserved in other plants or in other species. For example, is there RNA-Seq data or NLS prediction in other plants that support the existence of SIZ1 variants located to the plasma membrane?

(Response to reviewer)

Thank you for your comment. Based on your suggestion, we conducted an analysis of the splicing variants of *Arabidopsis thaliana* SIZ1 orthologues in rice, corn, soybean, tomato, human, mouse, and yeast. We observed that the NLS motif was spliced out in certain splicing variants. Additionally, we found various splicing variants where the SAP domain or PINIT motif was spliced out. The results and discussions regarding this analysis have been included in the "Results" and "Discussion" sections, respectively. The corresponding data has been provided as Supplemental Fig. S13.

4. I am surprised that the transgenic plants expressing SSV2 (under the control of native or 35S promoter) display such a clear phenotype while there seems to be little difference in the thermotolerance of siz1-2, wild-type and SIZ1 expressing plants. This looks more like a gain-of-function of the SSV2-expressing plants than a rescue experiment. Could the authors discuss the causes of such differences?

(Response to reviewer)

Thank you for your comment. We agree with you.

Under normal growth conditions, the transcript level of *SSV2* is relatively very low compared to the transcript level of *SIZ1*. However, under heat stress conditions, the transcript level of SIZ1 increases about 2-fold, while that of *SSV2* is dramatically increased up to 10-fold. Even under heat stress conditions, the transcript level of *SIZ1* remains approximately 2-fold higher than that of *SSV*2. Thus, we propose that both SIZ1 and SSV2 contribute to heat tolerance. However, due to their subcellular localization, they may function in different cellular compartments under heat stress conditions. SIZ1 primarily acts as an E3 SUMO ligase in the nucleus and cytoplasm, while SSV2 functions as an E3 SUMO ligase in the cell membrane.

In our experiments, we initially utilized the *SIZ1* promoter to express *SIZ1* or *SSV2*. In this case, the transcript level of *SSV2* in transgenic *siz1-2* mutants harboring *pSIZ1-Myc*$_6$*-SSV2* is comparable to that of *SIZ1* in transgenic *siz1-2* mutants harboring *pSIZ1-Myc*$_6$*-SIZ1*. This indicates that the transcript

level of *SSV2* in transgenic *siz1-2* mutants harboring *pSIZ1-Myc$_6$-SSV2* is significantly higher than that of *SSV2* induced in the wild type under heat stress conditions. Next, we employed the *35S* promoter to express these genes. As expected, the transcript level of *SSV2* in transgenic *siz1-2* mutants harboring *35S-Myc$_6$-SSV2* is higher than that of *SSV2* in transgenic *siz1-2* mutants harboring *pSIZ1-Myc$_6$-SSV2* or that of *SSV2* induced in the wild type under heat stress conditions, resulting in high heat tolerance in transgenic *siz1-2* mutants harboring *35S-Myc$_6$-SSV2* compared to the wild type.

Furthermore, we found that transgenic *siz1-2* mutants harboring *pSIZ1-Myc$_6$-SSV2* or *35S-Myc$_6$-SSV2* exhibited greater heat tolerance than transgenic *siz1-2* mutants harboring *pSIZ1-Myc$_6$-SIZ1* or *35S-Myc$_6$-SIZ1*, indicating that the sumoylation of target proteins by SSV2 as an E3 SUMO ligase, or its other functions in the plasma membrane, are important for heat tolerance.

Again, the production of *SSV2* and *SSV5* transcripts is induced under heat stress conditions, which is sufficient for heat tolerance. Additionally, the SSV2 protein level is much higher in transgenic *siz1-2* mutants harboring *pSIZ1-Myc$_6$-SSV2* or *35S-Myc$_6$-SSV2* than in the wild type, both under normal and heat stress conditions, despite the induction of SSV2 protein under heat stress conditions. This results in increased heat tolerance in *SSV2*-expressing transgenic *siz1-2* mutants compared to the wild type.

Therefore, we cannot rule out the possibility that the heat tolerance observed in transgenic plants expressing *SSV2* under the control of the native or *35S* promoter is a result of gain-of-function in the SSV2-expressing plants.

We have described the possibility of gain-of-function in the *SSV2*-expressing transgenic plants in the "Discussion" section as follows:

"The production of *SSV2* and *SSV5* transcripts was induced in WT plants under heat stress conditions, which contributes to the heat tolerance observed in WT plants. By the way, SSV2 protein was significantly higher in *pSIZ1-Myc$_6$-SSV2/siz1-2 or 35S-Myc$_6$-SSV2/siz1-2* plants compared to WT plants, both under normal conditions and even more so under heat stress conditions. This increased production of SSV2 protein resulted in enhanced tolerance in the *pSIZ1-Myc$_6$-SSV2/siz1-2* or *35S-Myc$_6$-SSV2/siz1-2* plants compared to WT plants. Hence, the observed improvements in heat tolerance phenotypes in the *SSV2*-expressing transgenic *siz1-2* plants, regulated by either the *SIZ1* or *35S* promoter, compared to *siz1-2*, wild-type, and *SIZ1*-expressing plants, could potentially be attributed to the gain-of-function effect resulting from *SSV2* overexpression".

Minor concerns:

1. Please indicate the number of replicates made for each experiment.

(Response to reviewer)

Thank you for your comment. Following your suggestion, we have provided information on the number of times each experiment was performed in the appropriate sections of the "Materials and Methods" section.

2. Please provide the catalogue number for all the antibodies used.

(Response to reviewer)

Thank you for your comment. Following your suggestion, we have included the sources and catalogue numbers of all the antibodies used for detecting the fusion proteins.

3. Why is the size of MBP-SSV2 about 150 kDa in Figure 7C while it is around 110 kDa in Figure 4B? Please check the position of the protein ladder.

(Response to reviewer)

We apologize for the mistake. There were errors in Figure 4. The corrected size of the recombinant protein MBP-SSV2 is approximately 155 kDa. We have now updated the size of MBP-SSV2 in Figure 4.

4. Page 12: “SSZ1 and all SSVs exhibit E3 ubiquitin ligase activity”: replace “ubiquitin’ by “SUMO”

(Response to reviewer)

We apologize for the mistake. We have now corrected the term “E3 ubiquitin ligase” to “E3 SUMO ligase”.

5. In the legend of Figure 6, replace “qRT-PCR” by “RT-PCR”.

(Response to reviewer)

We apologize for the mistake. We have now corrected “qRT-PCR” to “RT-PCR”.

6. In the legend of Figure 7, replace “HYP2” by “HPY2”

(Response to reviewer)

We apologize for the mistake. We have now corrected “HYP2” to “HPY2”.

7. In the legend of Figure 8, there is a work lacking between “were” by “with β-estradiol”.
(Response to reviewer)

We apologize for the mistake. We have now added the word “treated” in the sentence as requested: “Transgenic plants harboring *pCNGC6-CNGC6-GUS*/*XVE-SSV2* were treated with β-estradiol for the induction of *SSV2* expression.”

02-Jan-2024

Prof. Dr. Hak Soo Seo
Seoul National University
Plant Science
Seoul 08826
Korea (South), Republic of

RE: Role of Arabidopsis E3 SUMO ligase SIZ1 splicing variants: From localization to function

Dear Dr. Hak Soo Seo:

We are pleased to accept your manuscript for publication in the Plant Physiology. This acceptance is contingent on revision based on the comments of our reviewers. In particular, please consider the following:

Correct the figure 12, thanks

Please highlight all changes and include a detailed annotation to changes to the text, with line numbers, and noting your responses to the comments.

To submit your revised manuscript, click:

Link Not Available

If you cannot return the revised manuscript within 8 weeks of receipt, please let us know. Otherwise, we will assume that you have elected not to revise the manuscript and are withdrawing it.

Thank you for allowing us to review your work. We look forward to hearing from you soon.

Sincerely,

Plant Physiology Board of Editors

-- Reviewer comments:
Reviewer #1 (Comments for the Author):

The revised manuscript "Roile of Arabidopsis E3 SUMO ligase SIZ1 splicing variants: From localization to function" by Kwak et al. ist significantly improved. The changes go beyond a standard revision.

The changes are a significant advantage for the readers who have now a fairly complete story that goes from splicing variant, its localization to a substrate with impact on stress response.

Minor: Fig. 12A, images of PCR fragment gel are missing.

RE: Role of Arabidopsis E3 SUMO ligase SIZ1 splicing variants: From localization to function

Dear Dr. Hak Soo Seo:

We are pleased to accept your manuscript for publication in the Plant Physiology. This acceptance is contingent on revision based on the comments of our reviewers. In particular, please consider the following:

Correct the figure 12, thanks

Please highlight all changes and include a detailed annotation to changes to the text, with line numbers, and noting your responses to the comments.

To submit your revised manuscript, click:

https://pphys.msubmit.net/cgi-bin/main.plex?el=A7It1Nsm7A4DkZ3I2A9ftdIvetiLB8phoN7PE5iSiMwZ

If you cannot return the revised manuscript within 8 weeks of receipt, please let us know. Otherwise, we will assume that you have elected not to revise the manuscript and are withdrawing it.

Thank you for allowing us to review your work. We look forward to hearing from you soon.

Sincerely,

Plant Physiology Board of Editors

Reviewer #1 (Comments for the Author):

The revised manuscript "Roile of Arabidopsis E3 SUMO ligase SIZ1 splicing variants: From localization to function" by Kwak et al. is significantly improved. The changes go beyond a standard revision.

The changes are a significant advantage for the readers who have now a fairly complete story that goes from splicing variant, its localization to a substrate with impact on stress response.

Minor: Fig. 12A, images of PCR fragment gel are missing.

(Response to reviewer)

We apologize for the mistake. We have corrected Figure 12. The images of the PCR fragment gel have now been included in Figure 12A.

08-Jan-2024
Prof. Dr. Hak Soo Seo
Seoul National University
Plant Science
Seoul 08826
Korea (South), Republic of

MSID: PP2023-RA-01987DR1
MS TITLE: Role of Arabidopsis E3 SUMO ligase SIZ1 splicing variants: From localization to function

Dear Dr. Hak Soo Seo:

I am pleased to inform you that your revised manuscript "Role of Arabidopsis E3 SUMO ligase SIZ1 splicing variants: From localization to function" has been accepted for publication in Plant Physiology. Your manuscript will be checked for consistency with journal formatting and image standards by one of our Science Editors, who will contact you shortly. They may have a number of queries and suggestions for you. Please keep in mind that they work to increase the online visibility of your article and its attraction for readers from the widest of backgrounds. You will have the opportunity to review any revisions, and I hope you will work constructively with them to get the most out of your research. You will also have the opportunity to review all changes when you receive the author proofs for your article.

Once validated for publication, your article will be sent to a Science Editor for a final review of your manuscript and source files. After your final files are uploaded, your article will appear in the next available issue and the online Preview version of your article will be posted after your signed Copyright and Checklist forms and final source files are received.

Please be sure to include the following in your manuscript file:
Harvard style citations
List of all author contributions and funding information at the beginning of manuscript after the titles and author list.
List all supplemental materials associated with the article, with titles, at the end of the ms, before the references and acknowledgments section.
Complete Figure Legends for all figures appearing in the article listed before the Literature Cited.

Thank you and congratulations on your Plant Physiology paper!

Sincerely,
Plant Physiology Board of Editors

=====
FIRST AUTHOR PROFILES
=====

We recognize the hard work that goes into being First Author of a Plant Physiology paper by publishing a "First Author Feature" for each First Author alongside your paper.

If the first author(s) would like to submit information for a feature on Facebook and Twitter, fill out the form by clicking on the link below:

Plant Physiology First Author Feature Form: https://forms.office.com/r/ZBCGZfhm8F
If you cannot access the form through the link above, please contact Rachel at rbelsky@aspb.org.

To upload an image of yourself to accompany your profile, please put your photo in the correct journal folder at the link below. You must label your image: Author First Name Last Name Article Title.

First Author Profile Image Dropbox: https://www.dropbox.com/scl/fo/xz029p7qjr2jml2rp2kmq/h?dl=0&rlkey=6fkxcaqmxx35ap152b2osy1fz

=====

IMPORTANT REMINDER: PEER REVIEW REPORTS
=====

If you opted to publish a peer review report along with your article during the original submission process, it will be prepared by the editorial staff and publicly posted with your manuscript, inside the zip file that contains any other supplemental material. As a reminder, the peer review report is a public record of all comments from editors and reviewers, as well as your prior responses, as you received them in the decision letters for each draft of your manuscript. If you agreed to publish this report and have changed your mind, or are not sure if you selected this option, please contact the editorial office as soon as possible before signing the license agreement from our publisher.

-- Reviewer comments:
